# Whole transcriptome RNA-Seq analysis reveals extensive cell type-specific compartmentalization in *Volvox carteri*

**DOI:** 10.1186/s12915-017-0450-y

**Published:** 2017-11-28

**Authors:** Benjamin Klein, Daniel Wibberg, Armin Hallmann

**Affiliations:** 1 Department of Cellular and Developmental Biology of Plants, University of Bielefeld, Universitätsstr. 25, 33615 Bielefeld, Germany; 2 Center for Biotechnology (CeBiTec), University of Bielefeld, Bielefeld, Germany

**Keywords:** Cellular differentiation, Cell types, Gene expression, Green algae, RNA sequencing, Transcript level, Whole transcriptome sequencing, Volvocales, Volvocine algae, *Volvox carteri*

## Abstract

**Background:**

One of evolution’s most important achievements is the development and radiation of multicellular organisms with different types of cells. Complex multicellularity has evolved several times in eukaryotes; yet, in most lineages, an investigation of its molecular background is considerably challenging since the transition occurred too far in the past and, in addition, these lineages evolved a large number of cell types. However, for volvocine green algae, such as *Volvox carteri*, multicellularity is a relatively recent innovation. Furthermore, *V. carteri* shows a complete division of labor between only two cell types – small, flagellated somatic cells and large, immotile reproductive cells. Thus, *V. carteri* provides a unique opportunity to study multicellularity and cellular differentiation at the molecular level.

**Results:**

This study provides a whole transcriptome RNA-Seq analysis of separated cell types of the multicellular green alga *V. carteri* f. *nagariensis* to reveal cell type-specific components and functions. To this end, 246 million quality filtered reads were mapped to the genome and valid expression data were obtained for 93% of the 14,247 gene loci. In the subsequent search for protein domains with assigned molecular function, we identified 9435 previously classified domains in 44% of all gene loci. Furthermore, in 43% of all gene loci we identified 15,254 domains that are involved in biological processes. All identified domains were investigated regarding cell type-specific expression. Moreover, we provide further insight into the expression pattern of previously described gene families (e.g., pherophorin, extracellular matrix metalloprotease, and *VARL* families). Our results demonstrate an extensive compartmentalization of the transcriptome between cell types: More than half of all genes show a clear difference in expression between somatic and reproductive cells.

**Conclusions:**

This study constitutes the first transcriptome-wide RNA-Seq analysis of separated cell types of *V. carteri* focusing on gene expression. The high degree of differential expression indicates a strong differentiation of cell types despite the fact that *V. carteri* diverged relatively recently from its unicellular relatives. Our expression dataset and the bioinformatic analyses provide the opportunity to further investigate and understand the mechanisms of cell type-specific expression and its transcriptional regulation.

**Electronic supplementary material:**

The online version of this article (doi:10.1186/s12915-017-0450-y) contains supplementary material, which is available to authorized users.

## Background

The development and radiation of clonally developing multicellular organisms with different types of cells is one of evolution’s most important achievements [1–5]. Among the eukaryotes, simple multicellularity has evolved at least 25 times from unicellular ancestors, making such a development step less rare than might have been expected [1–3, 6–16]. Complex multicellularity with cell-cell adhesion, intercellular communication, and cellular differentiation has evolved ten times in eukaryotes – once in Animalia, three times in Fungi (chytrids, ascomycetes, and basidiomycetes), and six times in the three major photosynthetic clades [5], namely Phaeophyta (brown algae), Rhodophyta (red algae), and Viridiplantae (green algae and land plants). Evolution of cellular differentiation is a milestone through which two or more cell types with clear-cut identities arise from one embryonic cell accompanied by the loss of reproductive capacity in somatic cells. Prima facie, it is hard to understand how the waiving of reproductive capacity of many cells of an organism can be beneficial for the whole organism and, therefore, different theories about the evolution of cellular differentiation have emerged [1, 3, 4, 8, 9, 17–24].

In most lineages, the investigation of aspects of multicellularity and cellular differentiation at the molecular level are challenging since the transitions occurred too long ago and organisms have evolved numerous different cell types [25]. In contrast, multicellular members of the volvocine green algae group, such as *Volvox carteri*, diverged relatively recently from their unicellular relatives [23, 25, 26], thus representing a unique opportunity to study multicellularity and cellular differentiation at the molecular level. Furthermore, *V. carteri* exhibits a complete division of labor between mortal somatic cells and immortal germ cells. Given the above and further unique properties, *V. carteri* remains one of the simplest multicellular model organisms in developmental biology [8, 27–35].

**Fig. 1:**
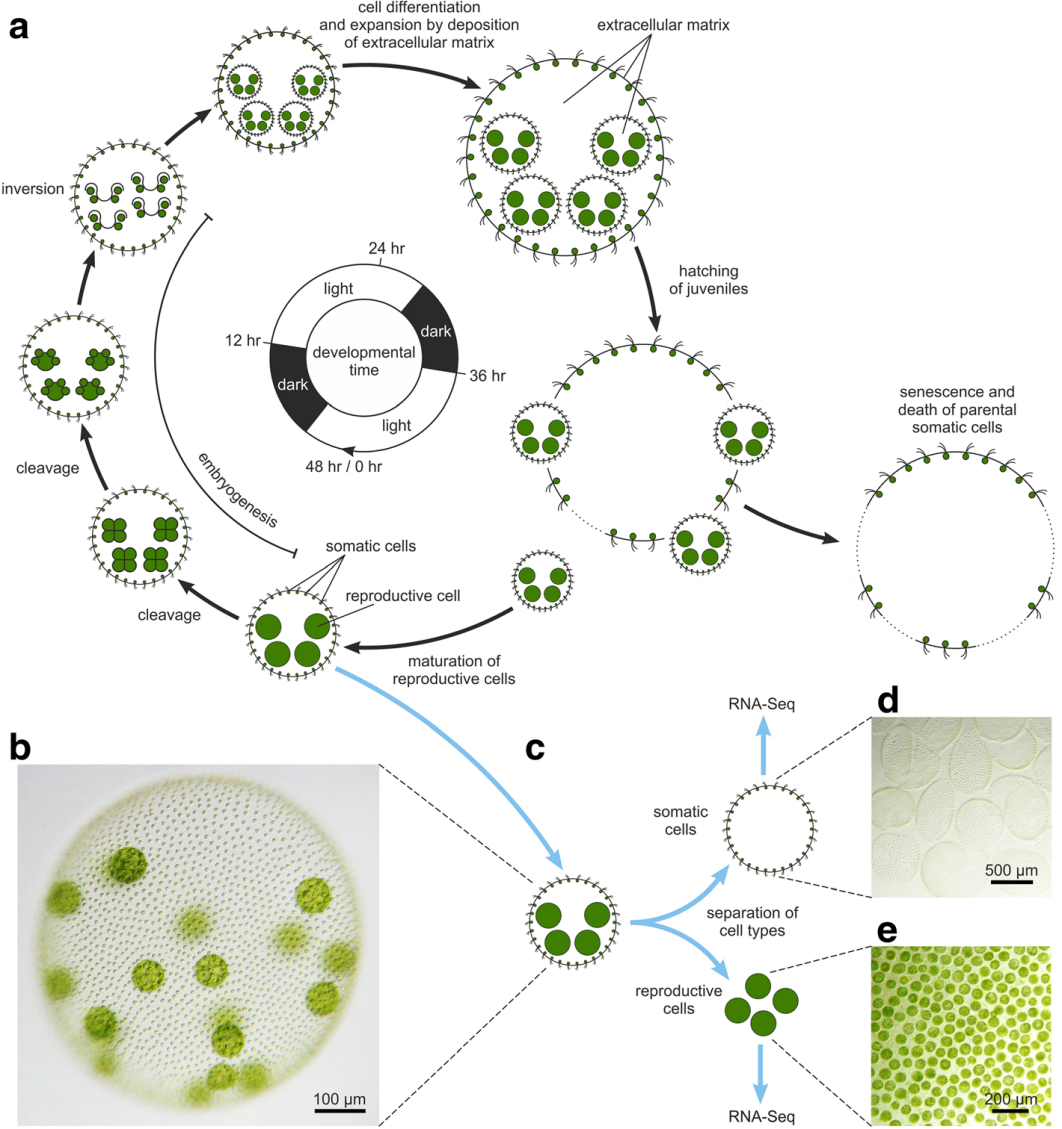
Asexual development of *Volvox carteri*, wild-type phenotype and separation of cell types. **a** Asexual development of *V. carteri* [8, 35]. *Volvox* algae exist as distinct males and females. However, during asexual development the males look just like the females (for sexual development see Additional file 1: Figure S1). During embryogenesis, mature asexual reproductive cells (gonidia) undergo a rapid series of 11–12 cleavage divisions, some of which are asymmetric. The fully cleaved embryo contains all of the cells of both types that will be present in an adult but it is inside out with respect to the adult configuration. This awkward condition is quickly corrected by a gastrulation-like inversion process [144]. Then, both the adult spheroid and the juvenile spheroids within it expand by the deposition of the extracellular matrix (ECM). The juveniles eventually hatch from their parent spheroid and the somatic cells of the parent undergo senescence and die, while the reproductive cells of the juvenile spheroids mature. Under standard conditions [117, 133, 134], the asexual life-cycle takes 48 h. For clarity, each parent spheroid in this figure contains only 4 of the ~16 reproductive cells, embryos, or descendant spheroids. **b** Wild-type phenotype of an asexual female of *V. carteri* containing approximately 2000 small, terminally differentiated, biflagellate somatic cells at the surface and approximately 16 large reproductive cells in the interior. The reproductive cells are at the developmental stage just before the beginning of embryogenesis. More than 95% of the volume of such a spheroid consists of a complex but transparent ECM. **c** Mechanical separation of the cell types of three biological replicates was performed at the developmental stage just before the onset of cell cleavage of reproductive cells. The separated cell types were then used for the RNA-Seq analysis. **d** Isolated somatic cell sheets. **e** Isolated reproductive cells

*V. carteri* is a spherically organized, mobile, obligate photoautotrophic alga of 0.5 to 2 mm in diameter, with a distinct male-female sexual dimorphism [8, 35]. In nature, it lives in freshwater ponds, puddles, and ditches, where it reproduces asexually as long as the conditions are favorable. An asexual cycle begins when each mature reproductive cell of an adult spheroid initiates a rapid series of cleavage divisions, some of which are asymmetric and produce large reproductive initials and small somatic initials (Fig. 1a). After completion of cleavage and cellular differentiation, the embryo needs to turn itself right-side out in a morphogenetic process called inversion. Following inversion, both the adult spheroid and the juvenile spheroids within it increase in size by depositing large quantities of extracellular matrix (ECM) (Fig. 1a). Finally, the juveniles hatch out of the parenteral spheroid and the asexual cycle starts again. However, when the habitat of an asexually reproducing *Volvox* population begins to dry out, e.g., in the heat of late summer, the algae switch to sexual reproduction and produce dormant zygotes with hard cell walls that survive the drought (Additional file 1: Figure S1). As soon as favorable conditions return, the zygotes undergo meiosis, germinate, and develop into asexually reproducing males or females. In the asexual mode of reproduction, both male and female algae contain approximately 2000 small, terminally differentiated, biflagellate somatic cells embedded in the surface of a transparent sphere of glycoprotein-rich ECM. Furthermore, approximately 16 large reproductive germ cells (called gonidia) are positioned slightly below the surface of the spheroid (Fig. 1b). Each cell has a single, large cup-shaped chloroplast to conduct photosynthesis [8]. The somatic cells are specialized for motility and phototaxis, incapable of dividing, and programmed to die when only a few days old, whereas reproductive cells are immotile, specialized for growth and reproduction, and potentially immortal [8, 27–35].

**Fig. 2:**
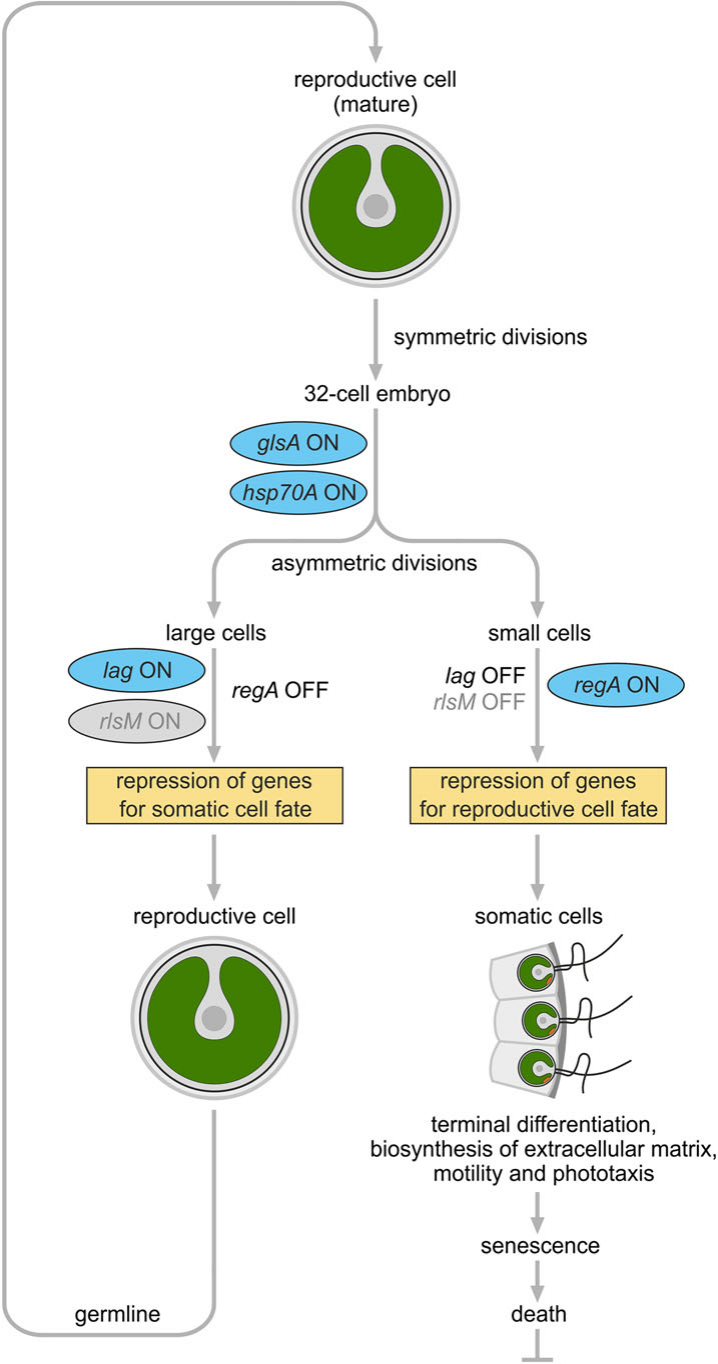
Minimal model for the genetic program of cellular differentiation in *V. carteri*. Four master regulatory genes are involved in programming differentiation, namely *glsA*, *hsp70A*, *lag*, and *regA* [8, 27, 30, 32, 33, 35–37, 39, 40, 42]. At the 32-cell stage, expression of *glsA* and *hsp70A* genes is required to promote the asymmetric divisions that produce large-small sister-cell pairs. Then, the *lag* gene acts only in the large cells to repress the development of somatic characteristics, while the *regA* gene acts only in the small cells to repress reproductive development. In contrast to the *glsA*, *hsp70A*, and *regA* genes, which have been cloned and sequenced [38, 40, 46], the *lag* gene is actually unknown. The role for *lag* in the model is based on previously existing phenotypic mutants [8, 27], but the phenotype-gene relationship is missing and, therefore, the *lag* gene itself is out of reach. However, if the counterpart of *regA* in large cells evolved from an ancient *regA* gene, then *rlsM* could be a candidate for the missing *lag* gene. As shown here, the *regA*-related *rlsM* gene is only expressed in large, reproductive cells

Based on molecular studies, a minimal model for the genetic program of cellular differentiation into somatic and germline cells in *V. carteri* has been established [8, 27, 30, 32, 33, 35–42] (Fig. 2). The model includes four master regulatory genes, namely *glsA*, *hsp70A*, *lag*, and *regA.* After several symmetric cell divisions, *glsA* and *hsp70A* genes act to shift cell-division planes in one half of the embryo, resulting in the asymmetric divisions that set apart large-small sister-cell pairs. After cleavage divisions, cell specialization results from cell size-specific expression of the regulatory genes *lag* and *regA*, which are supposed to code for transcriptional repressors. The *lag* gene acts only in the large cells to repress the development of somatic characteristics, while the *regA* gene acts only in the small cells to repress reproductive development. After activation of either a somatic or germline program, small cells develop into biflagellate somatic cells and large cells develop into non-motile germline cells.

Although this minimal model is very helpful, it is only an interim outcome towards complete understanding of cellular differentiation in *V. carteri*. It remains unclear which other components are involved and how the identified master regulatory genes fit into a larger regulatory network that governs cell type-specific gene expression levels. Over 30 years ago, David and Marilyn Kirk [43] recognized that it is also necessary to identify the genes or proteins that are expressed differentially in the two cell types in order to better understand cellular differentiation. At that time, they showed that somatic and reproductive cells of *V. carteri* display substantially different patterns of both newly synthesized and accumulated proteins [43]. However, it was not possible to obtain amino acid sequences of these proteins, so their identity remained unknown. The first cell type-specific expressed mRNAs of *V. carteri* were identified by northern blots using radiolabeled restriction-digested DNA as probes [44]. However, the investigators identified only approximately 30 different mRNA species and they did not obtain the sequence of these mRNAs. Without a sequence, the molecular functions of these mRNAs remained unresolved. A few years later, 18 mRNAs with cell type-specific expression in reproductive cells were sequenced and functionally classified [45]. Remarkably, these mRNAs turned out to be expressed both in reproductive cells and *regA* ^–^ mutant somatic cells, but not in *regA* ^+^ wild-type somatic cells. Moreover, many of these mRNAs encoded chloroplast proteins. These findings contributed to the current model for somatic cell differentiation (Fig. 2) involving repression of genes for reproductive development, whereby several of these genes are required for chloroplast biogenesis [45]. The *regA* gene and its gene product, which acts as key regulator in small cells (later somatic cells) to suppress reproductive development, have been identified by analyzing mutants and by Mendelian analysis [46] (Fig. 2). In a similar way, another key regulator, the lag protein, which acts in large cells (later reproductive cells) to repress somatic development, has been characterized [8, 47, 48] (Fig. 2).

In 2006, approximately 40 genes with quite different functions were characterized by quantitative real-time RT-PCR with respect to cell type-specific expression [49]. Even if the number of investigated genes is low, it is the largest analysis on mRNA expression of separated cell types in *Volvox* so far. Beyond that, only an additional 12 genes of *Volvox* have been analyzed in the same way [50].

Although large-scale transcriptome analyses have already been performed in *V. carteri*, they did not deal with cell type-specific mRNAs but had their own different objectives. Large-scale transcriptome analyses using expressed sequence tags were utilized to develop and confirm gene models [16] and to explore alternative splicing in *Volvox* [50]. However, these large-scale analyses could not provide any information about cell type-specific expression because the mRNA came from whole organisms. Even large-scale transcriptome analyses using RNA sequencing data and small RNA sequencing data have been generated in *Volvox* but only Argonaute 3-associated microRNAs have been analyzed for cell type-specific expression [51].

Here, we show a whole transcriptome RNA-Seq analysis of separated cell types of the multicellular alga *V. carteri* f. *nagariensis* to reveal cell type-specific mRNAs and their functions. We provide valid expression data for 93% of the 14,247 gene loci in *V. carteri*. Furthermore, all expressed genes were searched for known protein domain encoding sequences and we present which identified domains show cell type-specific expression. Since the scientific literature contains information on or at least a brief mention of approximately 400 *Volvox* genes, we look at the expression of those genes in more detail. In this connection, we also provide further insight into the expression pattern of previously described gene families, such as pherophorin, ECM metalloprotease, and *VARL* (volvocine algal *reg*A like) families. Overall, our results demonstrate an extensive compartmentalization of the transcriptome between cell types, since more than half of all genes show a clear difference in expression between somatic and reproductive cells.

## Results

### RNA isolation and high throughput sequencing

The objective of our study was the generation of global gene expression profiles of somatic cells and reproductive cells of *V. carteri* separately from each other. Mechanical separation of the cell types of three biological replicates was performed at the developmental stage just before the onset of cell cleavage of reproductive cells (Fig. 1a, c; procedure see Methods); only this stage allows for separation of somatic and reproductive cells (Fig. 1d, e).

Total RNA was extracted separately from both isolated cell types of each of the three biological replicates. All of these six samples passed the subsequent RNA quality controls and RNA-Seq libraries were prepared. Massively parallel sequencing of the six independent samples was performed on an Illumina HiSeq2500 system and the sequenced reads were quality filtered (see Methods). The RNA-Seq read filtering statistics are shown in Table 1. In total, 284 million reads passed the quality control. Of this total number of reads, 137 million reads came from somatic cells and 147 million reads from reproductive cells.

**Table 1:** RNA-Seq read filtering and mapping statistics

	Reproductive cells (three biological replicates)			Somatic cells (three biological replicates)			Reproductive cells	Somatic cells	In total
	Replicate A	Replicate B	Replicate C	Replicate A	Replicate B	Replicate C	(Combined)	(Combined)	
Total reads	40,833,921	40,714,201	57,614,694	36,648,572	65,483,065	46,370,011	139,162,816	148,501,648	287,664,464
Discarded reads	501,235	483,274	919,875	444,799	996,545	553,422	1,904,384	1,994,766	3,899,150
% Discarded reads	1.2%	1.2%	1.6%	1.2%	1.5%	1.2%	1.4%	1.4%	1.4%
QC passed reads	40,332,686	40,230,927	56,694,819	36,203,773	64,486,520	45,816,589	137,258,432	146,506,882	283,765,314
% QC passed reads	98.77%	98.81%	98.40%	98.79%	98.48%	98.81%	98.63%	98.66%	98.64%
Mapped reads	36,161,648	36,226,084	50,293,801	32,963,913	48,795,947	41,687,864	122,681,533	123,447,724	246,129,257
% Mapped reads (vs. QC passed)	89.66%	90.05%	88.71%	91.05%	75.67%	90.99%	89.38%	84.26%	86.74%
% Mapped reads (vs. total reads)	88.56%	88.98%	87.29%	89.95%	74.52%	89.90%	88.16%	83.13%	85.56%

*QC* quality control

### Mapping and analysis of expression data

The obtained quality filtered 284 million reads of both cell types were attempted to be mapped onto the *V. carteri* f. *nagariensis* genome assembly v2 [16]. The RNA-Seq mapping statistics are shown in Table 1. In total, 246 million reads were successfully mapped to the *Volvox* genome, which corresponds to 87% of the reads that passed the quality control. Of this total number of mapped reads, 123 million reads came from somatic cells and 123 million reads from reproductive cells. Thus, both cell types contributed the same number of mapped reads.

Expression analysis and visualization was performed by using the short-read mapping analysis platform ReadXplorer 2.2.3 [52]. The mapped reads hit 14,203 out of the 14,247 predicted genes of the *V. carteri* genome (annotation v2.1) on the Phytozome V12 platform [53], which corresponds to 99.7% of all predicted genes. For each of the 14,203 genes with expression data, the absolute intensity of expression was determined using the mean of normalized counts of both cell types with three biological replicates each.

To allow for a robust expression analysis, the expression level had to exceed a certain minimum expression threshold corresponding to a baseMean value of 12.5 as computed by the R package DESeq [54–56]. The baseMean describes the mean normalized expression level of a given transcript, averaged over all replicates from both cell types. Applying this minimum expression threshold, 13,204 out of the 14,247 predicted genes showed adequate coverage for quantitative analysis of expression, which corresponds to 92.7% of all predicted genes.

In addition to the absolute intensities of expression, the fold differences in expression between somatic cells and reproductive cells were calculated. More precisely, we identified the genes that showed both a fold difference in expression of 2 or more and an adjusted significance value (*P* value) of 0.05 or less. This requirement was fulfilled by 7820 out of 14,247 predicted genes (55%). After applying the baseMean minimum expression threshold of 12.5 (see above), 7691 genes remained or, in other words, at least 54% of all genes showed a clear difference in expression between somatic cells and reproductive cells.

**Fig. 3:**
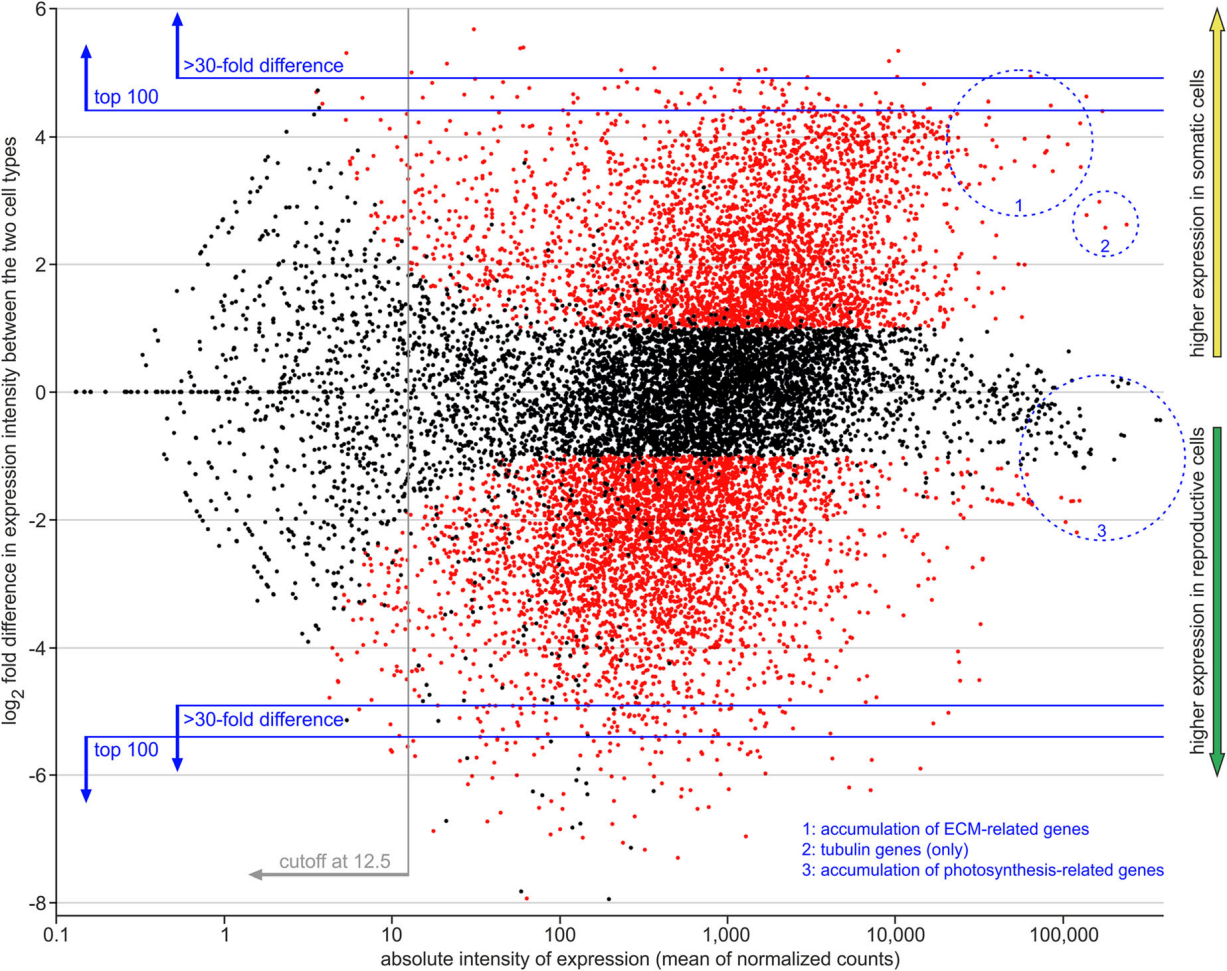
MA-plot of genome-wide gene expression data. MA-plot (Bland-Altman plot) for visual representation of both absolute expression intensity of each gene and differences in expression of each gene between somatic and reproductive cells. Each point in this two-dimensional plot shows the relationship between two sets of data: M-values (Y-axis) represent the log_2_ fold difference in expression intensity of a given gene between the two cell types (somatic versus reproductive cells), and A-values (X-axis) represent the absolute intensity of expression (mean of normalized counts) of the same gene in logarithmic scale. Genes with positive M-values show higher expression in somatic cells compared to the other cell type (yellow arrow) and genes with negative M-values show higher expression in reproductive cells compared to the other cell type (green arrow). The test for differential expression was based on DEseq calculations [54] and Benjamini–Hochberg multiple testing adjustment [145]. The false discovery rate value was set to q = 0.1. Points in red color refer to genes with significant differential expression (fold difference in expression ≥ 2 and *P*_adjusted_ ≤ 0.05), whereas black points refer to genes without significance regarding differential expression. An average baseMean expression value greater than 12.5 was sufficient for robust expression analysis (cutoff at 12.5). Blue lines indicate, for each cell type, both the top 100 most overexpressed genes compared to the other cell type and genes with a more than 30-fold difference in expression intensity compared to the other cell type. Blue, dotted circles indicate accumulations of functionally related genes

To provide an overview of the entire expression analysis, the expression data of all 14,203 genes with mapped RNA-Seq reads were visualized in a plot of log-intensity ratios (M-values) versus log-intensity averages (A-values) (MA-plot) (Fig. 3). The MA-plot shows both absolute expression intensity of each gene and differences in expression of each gene between somatic and reproductive cells. Genes with similar expression levels in both cell types (i.e., more precisely, without significance regarding differential expression) appear as black points around the horizontal zero line, whereas genes with significant differential expression are shown as red points (Fig. 3); functionally linked genes occasionally cluster in the same area of the MA-plot. Here, we identified accumulations of ECM-related genes, tubulin genes, and photosynthesis-related genes (Fig. 3).

### Investigation of gene structures

The mapped reads of the RNA-Seq analysis also offer information about exon-intron gene structures. However, the determination of gene structures is limited to genes with good coverage by mapped reads and, therefore, the expression needs to exceed a certain expression threshold corresponding to a baseMean value of 450 as computed by the R package DESeq [54–56].

To gain an overview of how well our mapped RNA-Seq data, which produce the expression profiles, match the exons of the predicted *Volvox* genes of annotation v2.1 on the Phytozome V12 platform, we used the software suite BEDTools *intersect* [57] for a genome-wide examination of overlaps. The analysis showed that 87% of our mapped data had an overlap with a predicted exon. However, 13% mapped outside predicted exons, but most of these mappings were localized to the UTRs (e.g., due to a 3’-UTR that is actually longer than predicted).

**Fig. 4:**
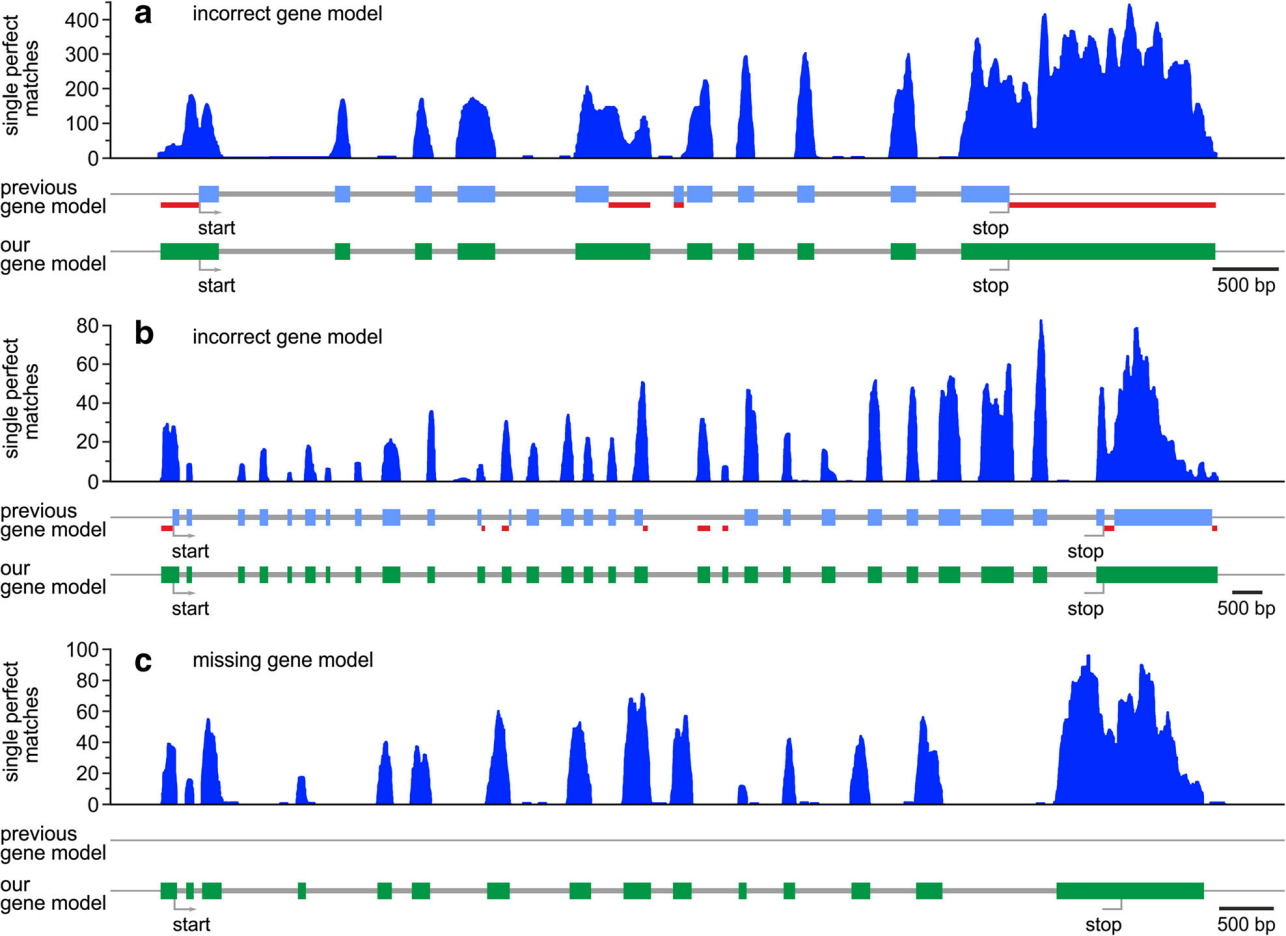
Examples of expression profiles of genes with previously absent or incorrect gene models. The previous gene models originate from gene annotation v2.1 of the *Volvox carteri* genome available on the *Volvox* pages of the Phytozome V12 platform. **a** Example of a gene with an incorrectly predicted gene model: the gene model of gene Vocar.0007s0316 is not in accordance with the expression profiles at exon 5 (prediction too short), exon 6 (non-existent in reality), and the 5’- and 3’-UTRs (no prediction). **b** Example of another gene with an incorrectly predicted gene model: the gene model of gene Vocar.0001s0415 is not in accordance with the expression profiles at exons 11, 12, and 17 (prediction too short). There are also inconsistencies on the 5’-UTR (no prediction) and on the 3’-UTR (prediction somewhat too short). Moreover, there are in fact two additional exons between exons 17 and 18 (no prediction), and there is definitely no intron within the 3’-UTR. **c** Example of a gene with previously absent gene model: the gene is located on scaffold 4 at position 1,393,429 to 1,402,937. It codes for a protein with 837 amino acid residues. In all panels (a–c), the previous exon-intron predictions for these genes according to annotation v2.1 of the *V. carteri* genome are given directly below the expression profiles; exons are shown as blue bars and introns as thick gray lines. Thick red lines indicate differences between the previous prediction according to annotation v2.1 and our prediction. Our own exon-intron predictions for these genes are shown at the bottom; exons are illustrated as green bars and introns as thick gray lines. Our predictions are supported by the obtained expression profiles (single perfect matches) shown in dark blue

For a more detailed picture of the nature of these discrepancies, we individually and very closely checked all generated expression profiles found on the first 1 million base pairs of the randomly chosen scaffold 9 (Additional file 2: Table S1) (see Methods). This section of the genome covers approximately 100 gene loci. Moreover, we performed the same analysis for 100 randomly chosen gene loci from the complete list of previously known gene loci (Additional file 3: Table S2). Both examinations on a random basis revealed that approximately one fifth (21% and 17%, respectively) of the sufficiently expressed genes showed discrepancies within the coding sequence. Examples of previous incorrect gene predictions that affect the coding sequence and, thus, have strong impact on the deduced amino acid sequences, are shown in Fig. 4a, b. In addition, more than two-fifths (45% and 42%, respectively) of the critically inspected gene loci showed discrepancies within the UTRs, which in reality are frequently longer than predicted. We also identified genes with clear expression profiles that were not predicted at the corresponding genomic position according to gene annotation v2.1 of the *V. carteri* genome available on the *Volvox* pages of the Phytozome V12 platform (Fig. 4c).

We cannot extrapolate the results of these sample analyses to the whole genome, but we can say that, apparently, the prediction algorithms used in the previous computer-based genome-wide analysis did not produce perfect results. Our RNA-Seq data can serve as a reliable basis for manual verification of gene predictions.

### Gene expression of previously investigated *Volvox* genes

**Fig. 5:**
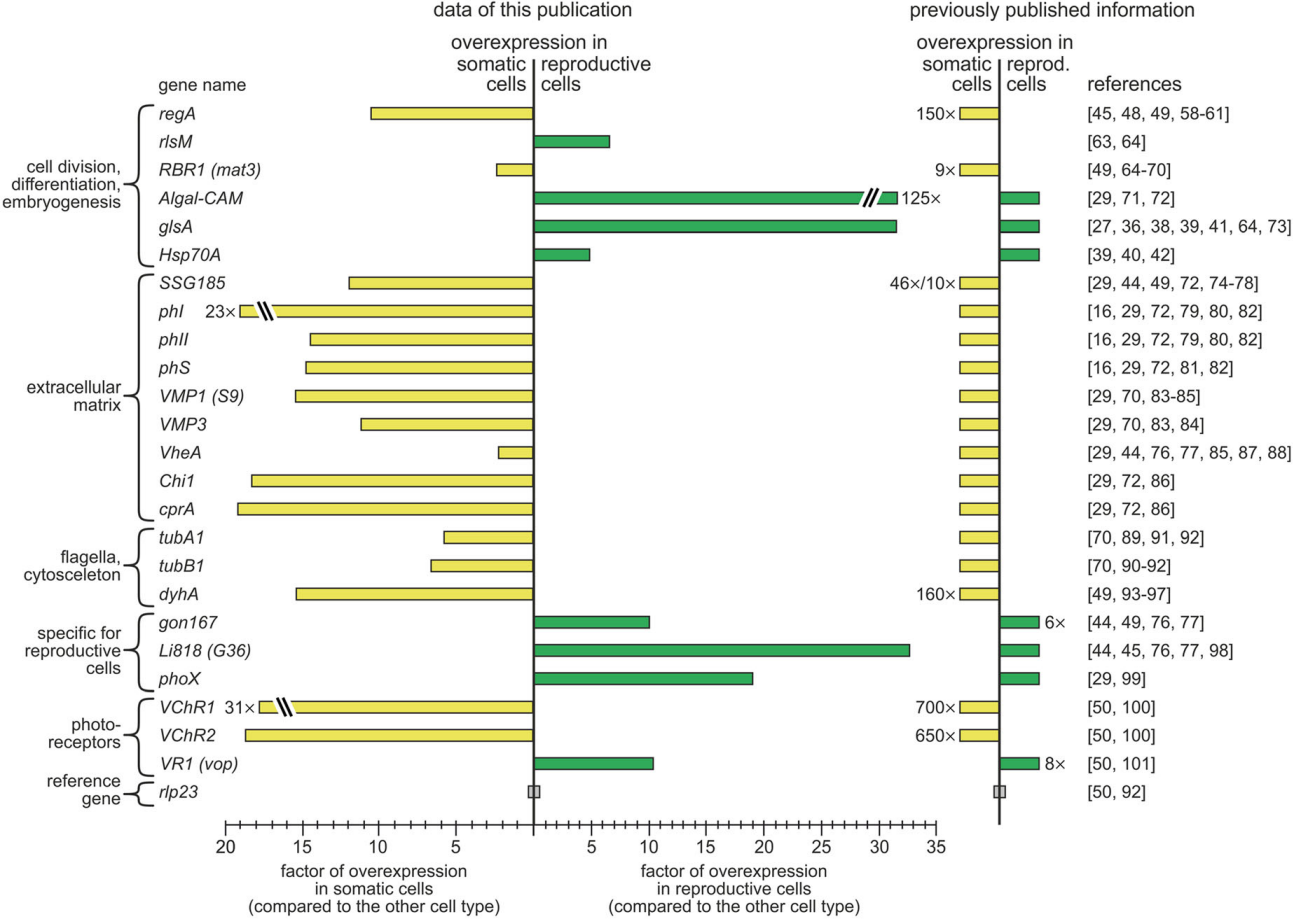
Cell type-specific gene expression of previously investigated *Volvox* genes. The figure shows expression data and references of the best-researched *Volvox* genes, which are arranged by gene function. These data were extracted from Additional file 4: Table S3, which contains a much more extensive table with expression data, further information, and references of 376 *Volvox* genes that are at least briefly mentioned in the literature. The length of the expression bar illustrates the fold higher expression of a given gene within the given cell type with respect to the other cell type. Yellow: higher expression in somatic cells; green: higher expression in reproductive cells. The previously published information about expression of these genes is presented in a qualitative, ‘digital’ format because they were either obtained qualitatively anyway or were quantified with a different experimental approach and/or developmental stage, which make comparisons rather difficult. Nonetheless, the numerical value of the previously determined fold difference in expression is indicated, if available

To provide new information about the expression of previously investigated *Volvox* genes and to validate our RNA-Seq dataset, we composed a list of all available *Volvox* genes that were at least briefly mentioned in the literature. All 376 *Volvox* genes of this list were investigated regarding cell type-specific gene expression and the results were compared to previous expression data if available (Additional file 4: Table S3). For clarity reasons, only the best-researched genes were collated and arranged by gene function in Fig. 5. The comparison illustrates that our results were qualitatively well fitted to previous results. It should be noted that the comparison needs to be performed qualitatively, because previous expression data were either obtained qualitatively anyway or were quantified with a different experimental approach and/or developmental stage. It is noticeable that especially genes that previously had a very high fold difference in expression, showed a more moderate fold difference in expression in our analysis.

Particularly, we checked several key genes in cell division, differentiation, and embryogenesis. The genes *regA* [42, 45, 46, 49, 58–64], *RBR1* (*mat3*) [49, 64–70], *Algal-CAM* [29, 71, 72], *glsA* [27, 36, 38, 39, 41, 64, 73], and *Hsp70A* [39, 40, 42] of this group showed the expected cell type-specific expression (Additional file 4: Table S3, Fig. 5). The *regA* gene is one of the best-studied genes in *Volvox*, coding for a transcriptional repressor [42, 45, 46, 49, 58–64]. It is important to mention that the expression analysis of the counterpart of *regA*, the *lag* gene (Fig. 2), is missing because it actually has never been sequenced or assigned to a specific gene locus, although it is frequently referenced [8, 27, 30, 32, 33, 35–37, 42]. The *regA* gene is a member of the *VARL* (volvocine algal *reg*A like) gene family due to a common VARL domain that includes a DNA-binding SAND domain [63]. The *VARL* gene family includes 14 members in *V. carteri*, which have not been previously subject to an expression analysis [63, 64]. Here, we show that 10 *VARL*-genes including *regA* are overexpressed in somatic cells, three *VARL*-genes show no clear cell type-specific expression, and a single gene, *rlsM*, is overexpressed in reproductive cells (Additional file 4: Table S3, Fig. 5). Due to its overexpression in reproductive cells, the *rlsM* gene is of particular interest because it could correspond to the missing *lag* gene. Like *regA*, *lag* was suggested to be a transcriptional repressor with a DNA-binding domain in the opposite cell type and both may have descended from the same ancestral gene [8, 27, 30, 32, 33, 35–37, 42]; *rlsM* fits these conditions.

Several genes of ECM (glyco)proteins have been previously investigated, including the pherophorins (e.g., *SSG185*, *phI*, *phII*, *phS*) [16, 29, 44, 49, 72, 74–82], ECM metalloproteases (e.g., *VMP1*, *VMP3*) [29, 70, 83–85], and other ECM enzymes (e.g., *VheA*, *Chi1*, *cprA*) [29, 44, 72, 76, 77, 85–88]. Due to previous experimental results and since ECM biosynthesis has been previously attributed to somatic cells only [29, 72, 79–82], all these genes were expected to be overexpressed in somatic cells [29], as indeed shown herein (Additional file 4: Table S3, Fig. 5). Regarding the pherophorins, it should be noted that we not only investigated the expression pattern of previously characterized pherophorins but also that of many others. Among the latter, we surprisingly identified pherophorins that are clearly overexpressed in reproductive cells (see below).

Tubulins (e.g., *tubA1*, *tubB1*) [70, 89–92] and dyneins (e.g., *dyhA*) [49, 93–97] are important components of flagella. Because only somatic cells have flagella, the genes of these proteins are expected to be expressed predominantly in somatic cells, as shown herein (Additional file 4: Table S3, Fig. 5). Likewise, we confirmed that genes that code for proteins known to be specific for reproductive cells (e.g., *gon167*, *Li818*, *phoX*) [29, 44, 45, 49, 76, 77, 98, 99] show overexpression in reproductive cells.

Photoreceptors (e.g., *VChR1*, *VChR2*) [50, 100] are known to be expressed predominantly in somatic cells, except for two weakly expressed photoreceptors that showed no cell type-specific expression and one, *VR1* [50, 101], which is overexpressed in reproductive cells. Our expression analysis is in accordance with these expectations, except for a lack of confirmation of the previous extremely high fold difference in expression of *VChR1* and *VChR2* (Additional file 4: Table S3, Fig. 5).

The gene *rlp23* [50, 92], which codes for a structural component of the ribosome, was suggested as a reference gene in expression analysis of different cell types (e.g., with real time qRT-PCR). Here, we show that *rlp23* is uniformly expressed in both cell types (Additional file 4: Table S3, Fig. 5), which confirms its suitability as a reference gene when target genes are examined for cell type-specific expression.

### Identification of the most highly expressed genes

**Fig. 6:**
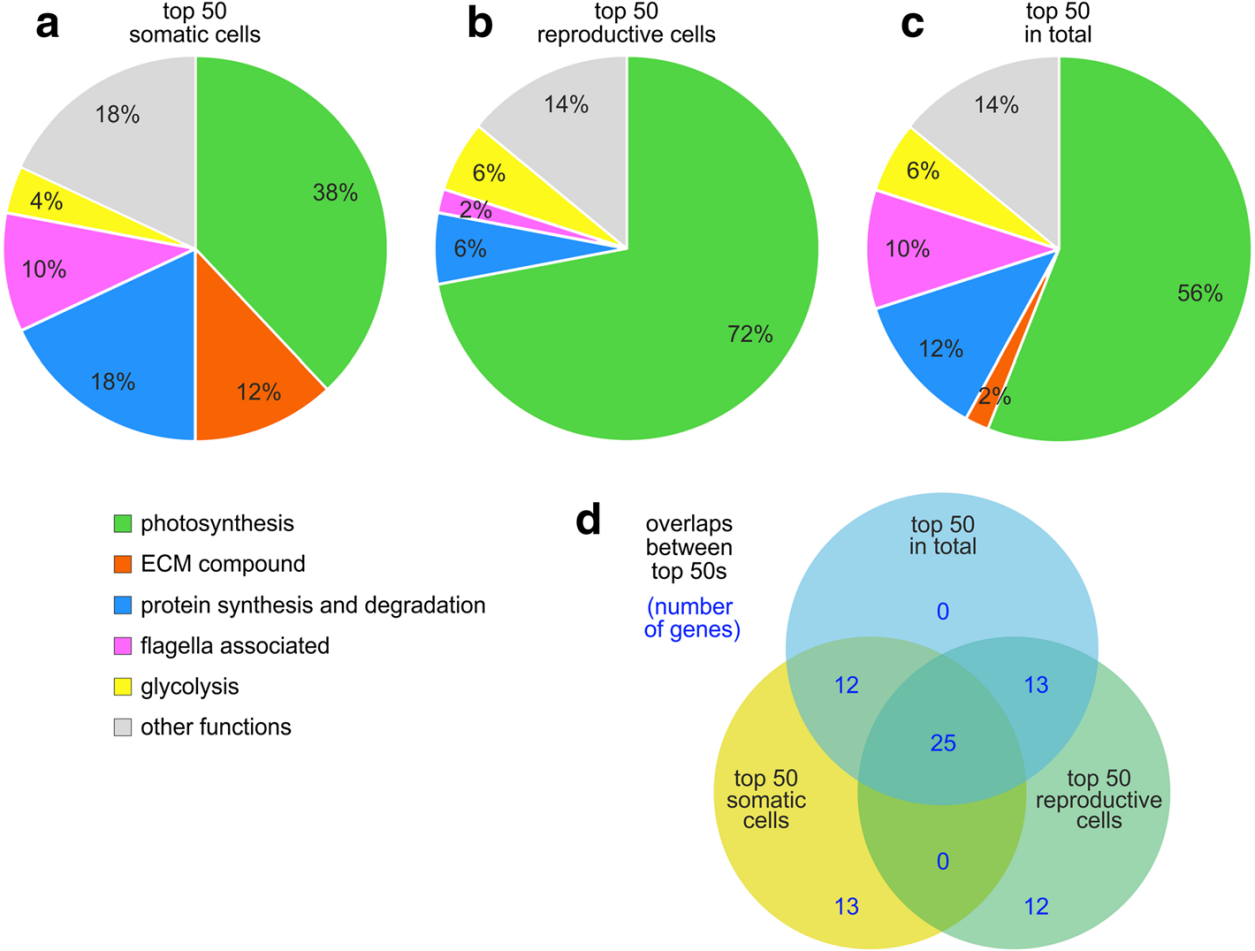
Functional enrichment analysis of the most highly expressed genes. The 50 most highly expressed genes in somatic cells, reproductive cells, and in total were classified based on Pfam, GO, and PANTHER assignments of gene annotation v2.1 on the Phytozome V12 platform. Because several genes came without functional gene annotation, those genes were subject to individual BLASTP searches and the annotation of the hit with the highest sequence similarity was used for classification. In this way, all involved genes obtained a functional assignment. Functional groups that contained not more than one member in both cell types were combined in the group ‘other functions’ for reasons of clarity. **a** Classification of the most highly expressed genes in somatic cells. **b** Classification of the most highly expressed genes in reproductive cells. **c** Classification of the most highly expressed genes in total. **d** Venn diagram illustrating overlaps between the three groups

To identify the most highly expressed genes in *V. carteri*, the RNA-Seq raw data were normalized both to the total read count per sample and to the transcript length [54]. The 50 most highly expressed genes in somatic cells, reproductive cells, and in total were subjected to a functional classification based on the Pfam [102–104], PANTHER [105–107], and GO [108, 109] assignments of gene annotation v2.1 of the *V. carteri* genome on the Phytozome V12 platform [53]. Genes without functional gene annotation were subject to individual BLASTP searches [110–112] and the annotation of the hit with the highest sequence similarity was used for classification. A functional enrichment analysis of the most highly expressed genes in each cell type and in total is shown in Fig. 6 and Additional file 5: Table S4.

In somatic cells, 19 out of the 50 most highly expressed genes (38%) were shown to encode photosynthesis-related proteins (Fig. 6a and Additional file 5: Table S4); 9 (18%) genes to encode proteins involved in protein synthesis and degradation, 6 (12%) ECM compounds, 5 (10%) flagella-related proteins, and 2 (4%) proteins of the glycolysis pathway. The remaining 9 (18%) genes were scattered across quite different functional groups.

In reproductive cells, 36 out of the 50 most highly expressed genes (72%) were shown to encode photosynthesis-related proteins, but none were shown to encode an ECM compound (Fig. 6b and Additional file 5: Table S4); 3 (6%) genes were shown to encode proteins involved in protein synthesis and degradation, 1 (2%) a flagella-related protein, and 3 (6%) proteins of the glycolysis pathway. The remaining 7 (14%) genes were scattered across quite different functional groups.

We also determined the most highly expressed genes in total, i.e., without taking the cell type into account (Fig. 6c and Additional file 5: Table S4). Also in this approach, genes encoding photosynthesis-related proteins formed the largest group (56%), followed by genes that encode proteins involved in protein synthesis and degradation (12%) and genes encoding flagella-related proteins (10%).

Overall, genes encoding photosynthesis-related proteins dominated all three groups of the top 50 most highly expressed genes. In somatic cells, several highly expressed genes that encode ECM compounds stood out, whereas no genes encoding ECM compounds were among the top 50 of reproductive cells. Genes encoding flagella-related proteins and proteins involved in protein synthesis and degradation were also more represented among the top 50 genes in somatic cells.

It is noteworthy that 25 genes belong to all the three groups of the top 50 most highly expressed genes. An overview of all intersections of the three groups is shown in Fig. 6d and all involved genes are mentioned by name in Additional file 5: Table S4.

### Detection of differentially expressed genes between somatic and reproductive cells

The MA-plot in Fig. 3 provides an overview of the genome-wide gene expression differences between somatic and reproductive cells and highlights the differentially expressed genes by using red dots. It is remarkable that more than half of all predicted genes (54%) showed a clear difference in expression between somatic cells and reproductive cells. The total number of 7691 genes with significant cell type-specific overexpression (and a baseMean expression value of at least 12.5) was split more or less evenly between 3728 genes overexpressed in somatic cells and 3963 genes overexpressed in reproductive cells (ratio 48.5:51.5). A further 129 genes showed cell type-specific overexpression (36 somatic, 93 reproductive) but did not reach the baseMean expression limit of 12.5.

**Fig. 7:**
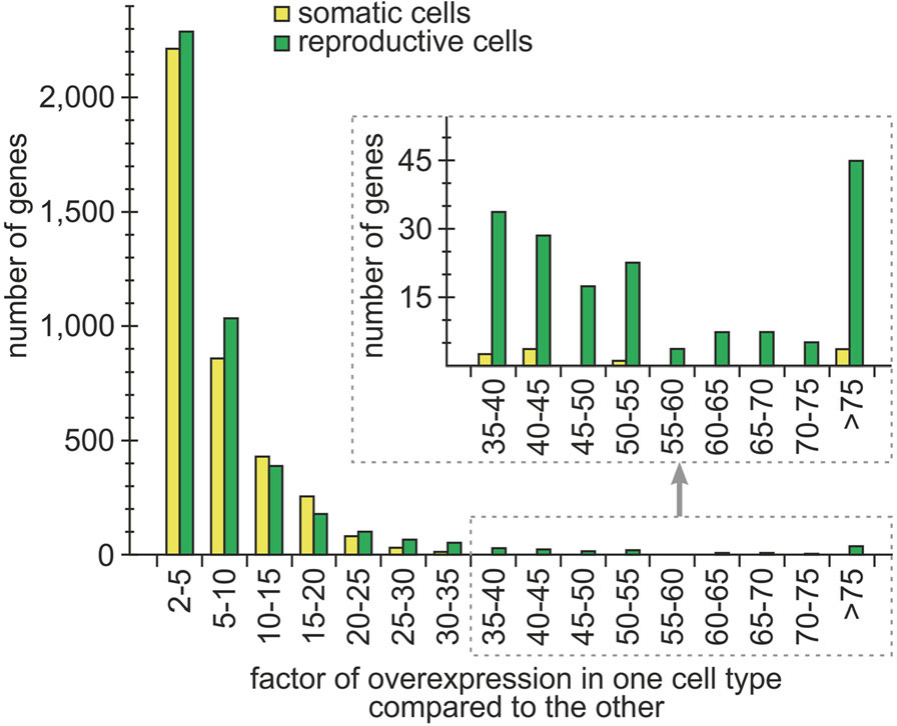
Classification of differentially expressed genes by their factor of overexpression. The differentially expressed genes were sorted in classes by their factor of overexpression in one cell type compared to the other and the number of genes was counted separately for each class and each cell type. Yellow columns: number of genes overexpressed in somatic cells. Green columns: number of genes overexpressed in reproductive cells. Only genes with differential expression were included (fold difference in expression ≥ 2). Inset: enlarged view of the framed portion of the main image

It is notable that the point cloud above the horizontal zero line did not have a mirror-image relationship with the point cloud below the zero line (Fig. 3). Obviously, there were more genes with a high factor of overexpression compared to the other cell type in reproductive cells than in somatic cells. To illustrate this effect more clearly, we identified all genes with more than 30-fold difference in expression compared to the other cell type (and a baseMean expression value of at least 12.5) (Fig. 3). Overall, 193 genes fulfilled these requirements, whereby 175 genes showed such a high factor of overexpression in reproductive cells whereas only 18 did so in somatic cells (ratio 90.7:9.3). Moreover, we identified the 100 most overexpressed genes of each cell type (Fig. 3) and determined the factor of overexpression of each of these genes. On average, the 100 most overexpressed genes in somatic cells showed 29-fold overexpression compared to the other cell type. However, the average value of the 100 most overexpressed genes in reproductive cells was much higher, showing 85-fold overexpression compared to the other cell type. To find the underlying cause, all genes with cell type-specific overexpression were sorted in classes by their factor of overexpression in one cell type compared to the other and the number of genes was counted separately for each class and each cell type (Fig. 7). From a more general perspective, the number of genes per class decreased with increasing factors of overexpression, which was to be expected. However, the number of genes with a high factor of overexpression was different between cell types. For the classes containing 20- to 25-fold overexpression and higher, a similar result was always obtained, wherein a greater number of genes had higher factors of overexpression in reproductive cells than in somatic cells. Actually, there were hardly any genes that exhibited a 35-fold or higher expression in somatic cells compared to reproductive cells (Fig. 7). In the classes containing 15- to 20-fold overexpression or less, there was a more balanced situation between the number of contributing genes from each cell type (Fig. 7).

### Functional enrichment analysis of the most differentially expressed genes

**Fig. 8:**
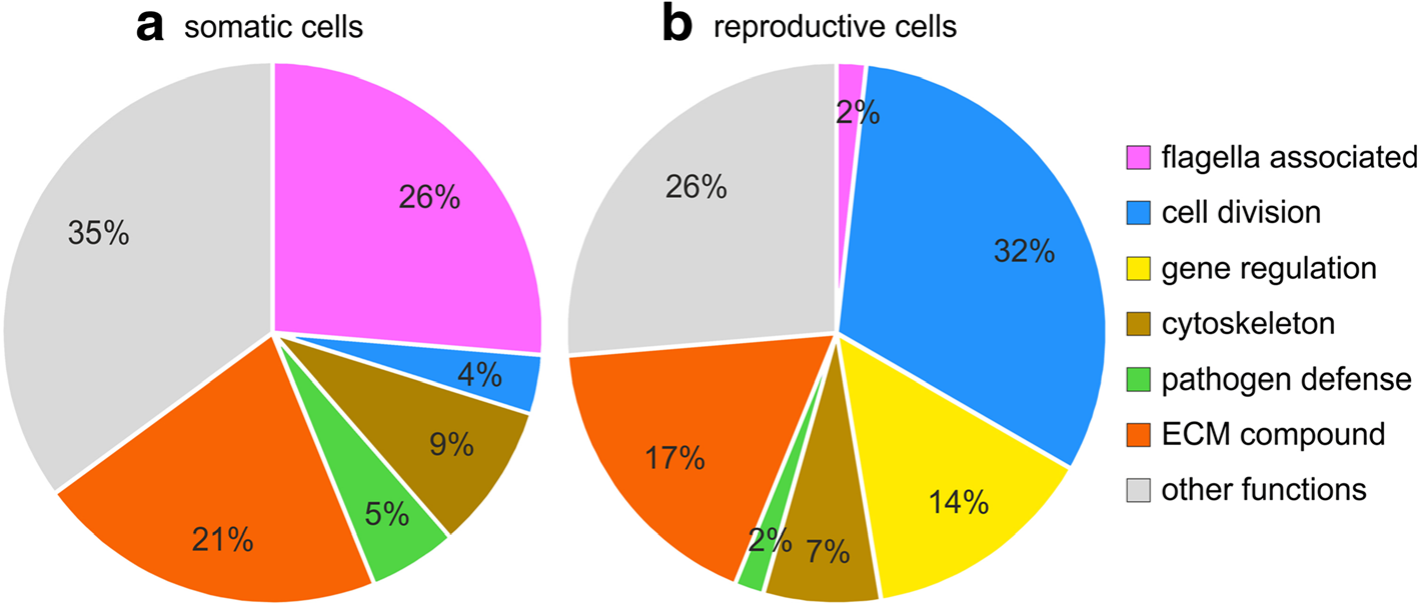
Functional enrichment analysis of the most overexpressed genes of both cell types. The 100 most overexpressed genes of each cell type were classified based on Pfam, GO, and PANTHER assignments according to gene annotation v2.1 on the Phytozome V12 platform. Because many genes came without functional gene annotation, those genes were subject to individual BLASTP searches and the annotation of the hit with the highest sequence similarity was used for classification. Nevertheless, genes without any BLASTP-hits remained and therefore had to be excluded from this analysis due to the missing functional classification. All other involved genes obtained a functional assignment. Functional groups that contained not more than one member in both cell types were combined in the group ‘other functions’ for reasons of clarity. **a** Classification of the most overexpressed genes in somatic cells. **b** Classification of the most overexpressed genes in reproductive cells

To get an idea of the function of the most differentially expressed genes in *V. carteri*, the 100 most overexpressed genes of each cell type were subject to a functional classification based on the Pfam [102–104], PANTHER [105–107], and GO [108, 109] assignments according to gene annotation v2.1 of the *V. carteri* genome on the Phytozome V12 platform. Because several genes came without functional gene annotation, those genes were subject to individual BLASTP searches [110–112] and the annotation of the hit with the highest sequence similarity was used for classification. Nevertheless, genes without any BLASTP-hits also remained and therefore had to be excluded from this analysis due to the missing possibility of functional classification. The results of the functional enrichment analysis of the most overexpressed genes of each cell type are shown in Fig. 8 and Additional file 6: Table S5.

The biggest functional group within the most overexpressed genes in somatic cells (compared to reproductive cells) were genes coding for flagella associated proteins (26%), followed by genes coding for ECM compounds (21%) and genes coding for components of the cytoskeleton (9%) (Fig. 8a). The biggest functional group within the most overexpressed genes in reproductive cells (compared to somatic cells) were genes related to cell division (32%) followed by genes coding for ECM compounds (17%), genes involved in gene regulation (14%), and genes coding for components of the cytoskeleton (7%) (Fig. 8b).

Remarkably, two major groups within the most overexpressed genes in reproductive cells, namely cell division and gene regulation, were only poorly (4%) or not at all represented in somatic cells (Fig. 8). In somatic cells, genes coding for flagella-associated proteins stood out, yet these were poorly (2%) represented within the most overexpressed genes in reproductive cells. It was expected that genes coding for ECM compounds would belong to the most overexpressed genes in somatic cells because it was assumed that somatic cells are solely or at least largely responsible for the biosynthesis of the extensive ECM [29, 72, 113]. However, genes coding for ECM compounds also represented a large proportion (21%) within the most overexpressed genes in reproductive cells (Fig. 8).

### Classification of all protein domains and screening for cell type-specific expression

**Fig. 9:**
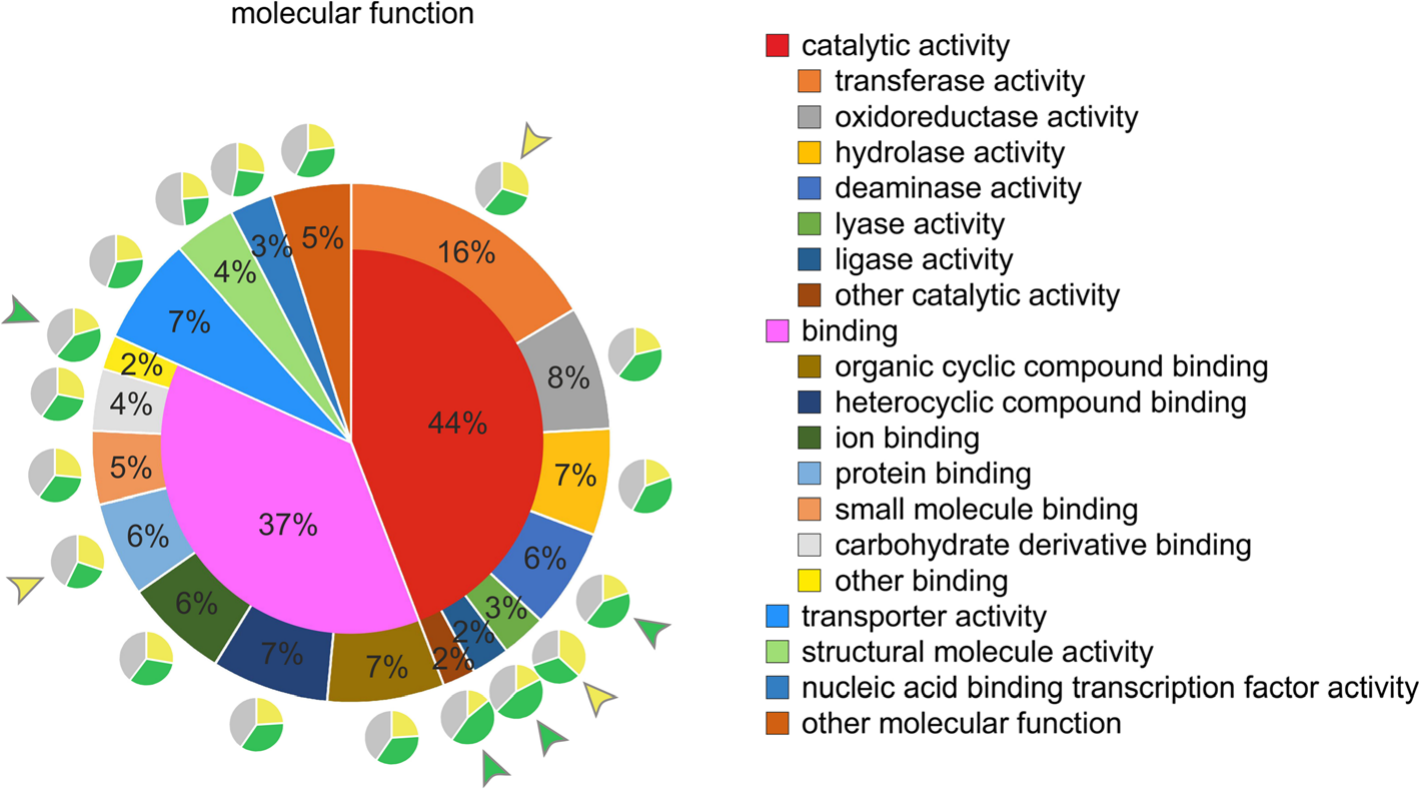
Protein domain classification by molecular function followed by analysis for cell type-specific distribution of expression. All genes were screened for assigned Pfam, GO, and PANTHER identifiers regarding their molecular function and the identifiers were assigned to higher level GO-terms. The identified protein domains were sorted into groups and subgroups using QuickGO. The percentage share of each group and subgroup within the total number of protein domains with assigned functions was determined. Small groups or subgroups with a percentage share of less than 2% were combined for reasons of clarity (see ‘other…’). The very large groups ‘catalytic activity’ (44%) and ‘binding’ (37%) are shown with their subgroups. Each group or subgroup was analyzed for the proportion of protein domains with overexpression (fold difference in expression ≥ 2) in somatic cells or reproductive cells, or without distinct differences in expression between the two cell types. Small pie charts show the results. Yellow color: share of protein domains that are overexpressed in somatic cells; green color: share of protein domains that are overexpressed in reproductive cells; gray color: share of protein domains without cell type-specific overexpression. Groups and subgroups that differ clearly from the distribution of the total quantity (deviation ≥ 20%) are highlighted by colored arrowheads. Yellow arrowhead: larger proportion of domains with overexpression in somatic cells. Green arrowhead: larger proportion of domains with overexpression in reproductive cells

Initially, all *Volvox* genes were screened for assigned Pfam, GO, and PANTHER identifiers regarding their molecular function and the identifiers were assigned to higher level GO-terms. A total of 6216 genes had at least one protein domain with an assigned molecular function and the total number of identified protein domains with assigned molecular function was 9435. The identified protein domains were sorted into groups and subgroups using QuickGO [114, 115]. The percentage share of each group and subgroup within the total number of protein domains with assigned molecular functions was determined (Fig. 9). Each group or subgroup was analyzed for the proportion of protein domains with overexpression (fold difference in expression ≥ 2) in somatic cells or reproductive cells, or without distinct differences in expression between the two cell types (Fig. 9). As a reference, we determined the cell type-specific proportions for all domains in ‘molecular function’ as a whole, which was 25.0% overexpressed in somatic cells, 34.2% overexpressed in reproductive cells, and 40.8% without overexpression. Groups and subgroups that differed clearly from this distribution of the total quantity (deviation ≥ 20%) are highlighted in Fig. 9. For example, protein domains with transferase activity, lyase activity, and protein binding showed a larger proportion of domains with overexpression in somatic cells (Fig. 9). Whereas protein domains with deaminase activity and ligase activity showed a larger proportion of domains with overexpression in reproductive cells (Fig. 9).

**Fig. 10:**
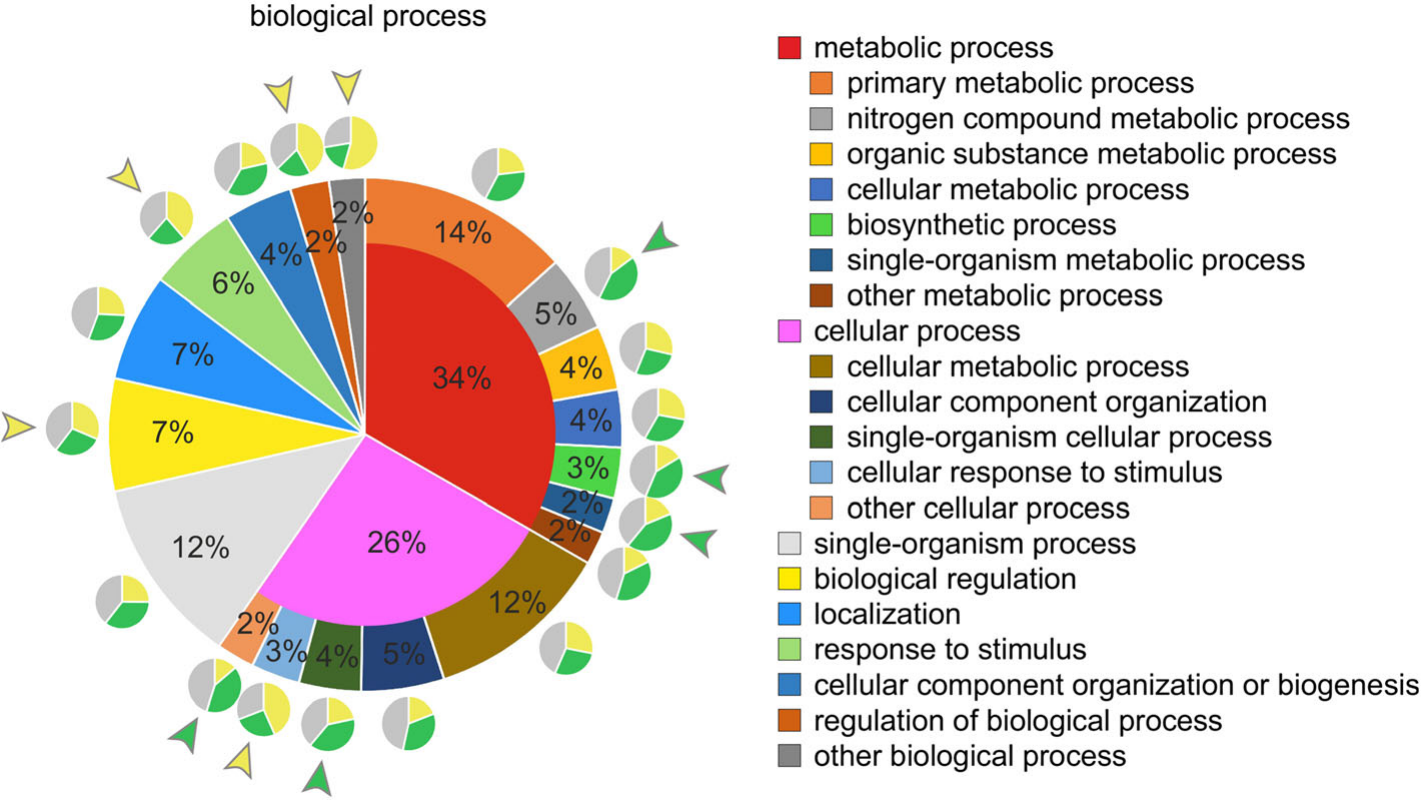
Protein domain classification by biological process followed by analysis for cell type-specific distribution of expression. All genes were screened for assigned Pfam, GO, and PANTHER identifiers with regard to their participation in biological processes and the identifiers were assigned to higher level GO-terms. The identified protein domains were sorted into groups and subgroups using QuickGO. The percentage share of each group and subgroup within the total number of protein domains with assigned functions was determined. Small groups or subgroups with a percentage share of less than 2% were combined for reasons of clarity (see ‘other…’). The very large groups ‘metabolic process’ (34%) and ‘cellular process’ (26%) are shown with their subgroups. Each group or subgroup was analyzed for the proportion of protein domains with overexpression (fold difference in expression ≥ 2) in somatic cells or reproductive cells, or without distinct differences in expression between the two cell types. Small pie charts show the results. Yellow color: share of protein domains that are overexpressed in somatic cells; green color: share of protein domains that are overexpressed in reproductive cells; gray color: share of protein domains without cell type-specific overexpression. Groups and subgroups that differ clearly from the distribution of the total quantity (deviation ≥ 20%) are highlighted by colored arrowheads. Yellow arrowhead: larger proportion of domains with overexpression in somatic cells. Green arrowhead: larger proportion of domains with overexpression in reproductive cells

Apart from the classification by molecular function, all *Volvox* genes were also screened for assigned identifiers regarding their participation in biological processes and the identifiers were again assigned to higher level GO-terms. A total of 6089 genes had at least one protein domain with an assigned biological process and the total number of identified protein domains with assigned biological process was 15,254. The identified protein domains were sorted into groups and subgroups and analyzed as described above. As a reference, we determined the cell type-specific proportions for all domains in ‘biological process’ as a whole, which was 26.4% overexpressed in somatic cells, 32.4% overexpressed in reproductive cells, and 41.2% without overexpression. Groups and subgroups that differed clearly from this distribution of the total quantity (deviation ≥ 20%) are highlighted in Fig. 10. For example, protein domains involved in responses to stimuli and regulation of biological processes showed a larger proportion with overexpression in somatic cells (Fig. 10). Whereas protein domains involved in nitrogen compound metabolic processes, biosynthetic processes, single-organism metabolic processes, and single-organism cellular processes showed a larger proportion with overexpression in reproductive cells (Fig. 10).

Overall, 24,689 protein domains were assigned to the 14,247 genes, which corresponds to an average of approximately 1.7 domains per gene. However, the real number is likely to be higher than 1.7 because new and undescribed domains, as well as domains with significant evolutionary changes, remain undetected.

**Fig. 11:**
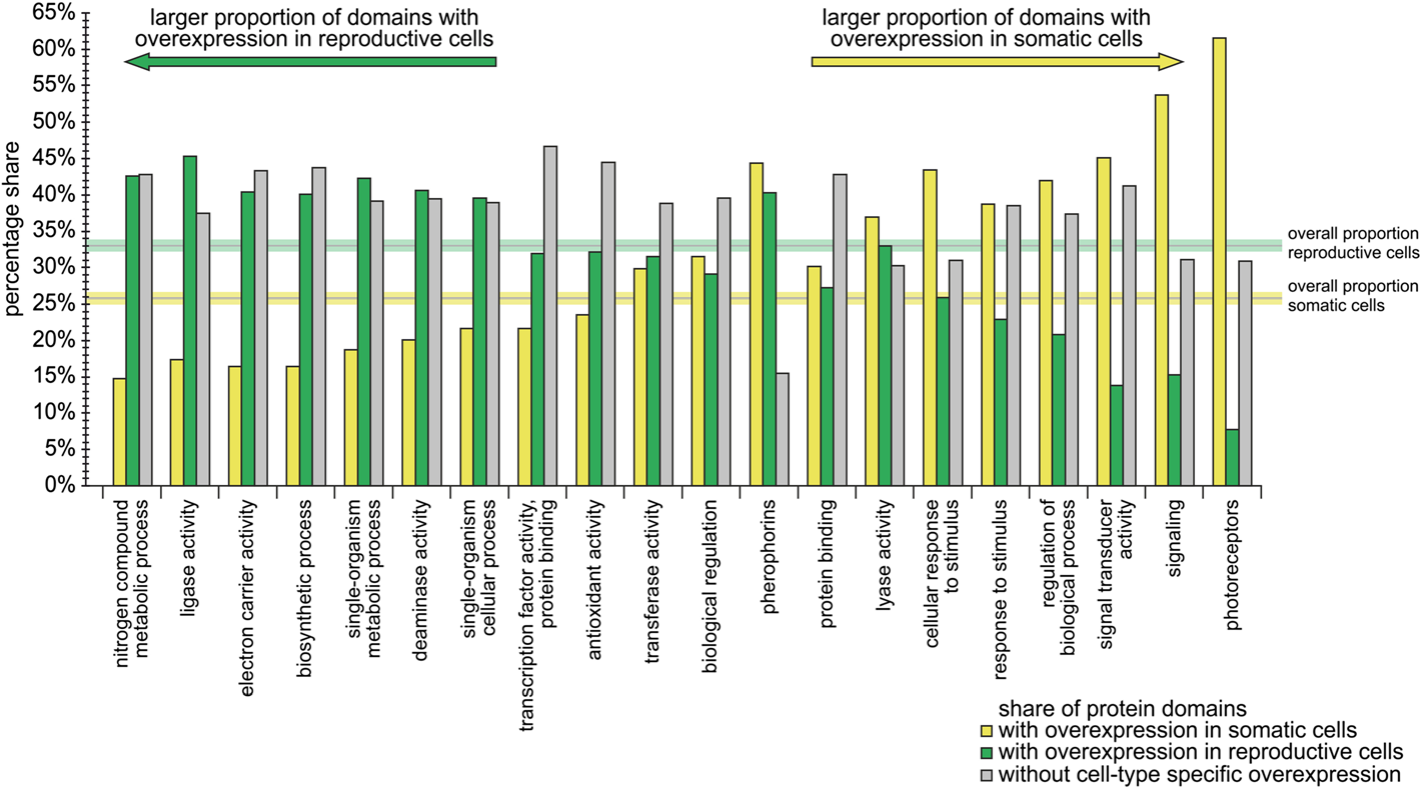
Proportions of cell type-specifically expressed domains in selected groups or subgroups of protein domains. As described in the legends of Figs. 9 and 10, protein domains were sorted into groups and subgroups and each group or subgroup was analyzed for the proportion of protein domains with overexpression (fold difference in expression ≥ 2) in somatic cells or reproductive cells, or without distinct differences in expression between the two cell types. Here, shares of cell type-specific expressed domains in selected groups or subgroups of protein domains are shown in greater detail. The groups are sorted from left to right by the percentage ratio of protein domains with overexpression in somatic cells to protein domains with overexpression in reproductive cells. For comparison, we also determined the proportions for all domains as a whole, which is 25.8% overexpressed in somatic cells (yellow horizontal line), 33.1% overexpressed in reproductive cells (green horizontal line), and 41.1% without overexpression. The listed groups/subgroups come from the classifications by both molecular function (Fig. 9) and biological process (Fig. 10). Some of the subgroups shown here are subgroups of the groups/subgroups shown in Figs. 9 and 10

To more clearly show the differences in the composition of groups or subgroups of protein domains regarding cell type-specific overexpression, we picked out 20 groups or subgroups and arranged them according to their composition. In Fig. 11, these groups or subgroups are sorted by the percentage ratio of protein domains with overexpression in somatic cells to protein domains with overexpression in reproductive cells. The clearest cell type-specific differences can be found in groups or subgroups that contain protein domains with very specific functions. Noticeable is the predominance of photoreceptor domains in somatic cells and the overweighting of domains with ligase activity in reproductive cells (Fig. 11). Particularly remarkable is also the absence of a clear cell type-specific imbalance in a group that contains domains of well-known ECM proteins, the pherophorins (Fig. 11). Previously, pherophorins have only been associated with ECM biosynthesis in somatic cells [29, 72, 79–82]. The issue is discussed below.

## Discussion

The focus of this study was a whole transcriptome RNA-Seq analysis of mechanically separated cell types of *V. carteri* f. *nagariensis* (Fig. 1) to disclose cell type-specific components and functions. After quality filtration, 246 million reads were mapped to the genome and 13,204 genes showed adequate coverage for quantitative analysis of expression. This study thus provides valid expression data for 93% of the total 14,247 *V. carteri* gene loci (Fig. 3). Moreover, our RNA-Seq data can serve as a reliable basis for manual verification of gene predictions (Fig. 4).

There is no doubt that mRNA expression and its regulation are essential for key developmental events such as cellular differentiation. However, an observed expression level of a particular gene can be cause or effect of a cellular condition or phenotype. Expression comparisons alone cannot distinguish between these possibilities. In such a situation, the identification and detailed characterization of key genes can help to unravel cause-and-effect networks. A detailed characterization of selected genes nevertheless involves expression analyses. In this respect, the analysis of cell type-specific gene expression of many previously investigated *Volvox* genes enabled us to provide new information about these genes (Additional file 4: Table S3, Fig. 5). The most thoroughly investigated genes with regard to cellular differentiation are *glsA*, *hsp70A*, *lag*, and *regA* [8, 27, 30, 32, 33, 35–42, 45, 46, 49, 58–64, 73] (Fig. 2). Both *glsA* and *hsp70A* are known to be expressed maximally in asymmetrically dividing embryos to shift cell-division planes but there is already a significant overexpression in reproductive cells before the onset of cell cleavage [27, 36, 38–42, 64, 73]. Our analysis clearly confirms this overexpression in reproductive cells prior to the onset of cell cleavage (Fig. 5). Based on the model for the genetic program of cellular differentiation (Fig. 2), cell specialization results from cell size-specific expression of the regulatory genes *lag* and *regA.* Therefore, these genes were suggested as key components added to the genome during evolution to make possible the conversion of the ancestral, sequential form of cyto-differentiation into the dichotomous form that characterizes *Volvox* [36]. The *regA* gene is expressed only in small cells to repress reproductive development and, thus, to produce somatic cells [42, 45, 46, 49, 58–64]. Accordingly, we observed a strong overexpression of *regA* in somatic cells (Fig. 5). The RegA protein was classified as a transcriptional repressor belonging to the VARL family [63, 64], which has 14 members in *V. carteri.* Our analysis provides the first expression data of the complete family. One of these VARL genes, *rlsM*, is particularly interesting because it is overexpressed in reproductive cells and, therefore, could correspond to the previously undiscovered *lag* gene [8, 27, 30, 32, 33, 35–37, 42]. The Lag protein has been presented and frequently referenced as a transcriptional repressor that acts in reproductive cells to prevent somatic development [8, 27, 30, 32, 33, 35–37, 42]; thus, Lag represents the counterpart of RegA. Previous work on Lag mainly deals with a characteristic mutant phenotype [8, 27, 37, 116] in which presumptive reproductive cells temporarily develop into larger-than-normal somatic cells with long flagella and large eyespots [8], similar to the RegA phenotype, in which presumptive somatic cells develop into reproductive cells [46]. Thus, the *rlsM* gene now appears as the most obvious candidate to be the undiscovered key gene that was previously named *lag*.

Two other key players in cell division and embryogenesis are *RBR1* (*mat3*) and *Algal-CAM. RBR1* is known to be involved in cell-size control of somatic cells [65]. Here, we confirm the overexpression of this gene in somatic cells (Fig. 5), even if the maximum expression of this gene is not expected prior to the onset of cell cleavage but later in embryogenesis [65]. Similarly, we confirm the overexpression of *Algal-CAM* in reproductive cells (Fig. 5). *Algal-CAM* is a cell adhesion molecule required in early embryogenesis [71].

Our actual results regarding cell type-specific expression of previously investigated genes coding for ECM proteins, flagella components, reproductive-cell specific proteins and photoreceptors are all in accordance with the expected results (Additional file 4: Table S3, Fig. 5). However, with regard to a large family of ECM proteins, the pherophorins, we also investigated new members that showed a cell type-specific expression behavior that was opposite to that of previously investigated pherophorins. This issue is further discussed below with regards to the most overexpressed genes.

The results regarding the most highly expressed genes are quite plausible. Since the obligate photoautotrophic *V. carteri* alga uses sunlight alone as the primary energy source, all required energy is provided by converting light energy into chemical energy by photosynthesis, which is conducted in the big chloroplast of each cell. As a consequence, it is reasonable that photosynthesis-related genes dominate the group of the most highly expressed genes in both cell types (Fig. 6a–c). A reproductive cell in the stage just prior to the onset of cell cleavage (Fig. 1a) requires sufficient energy for cell growth and for the forthcoming cleavage divisions. It should also be noted that, in *V. carteri*, reproductive cells grow far larger than somatic cells because a new juvenile is produced in a series of very rapid, synchronous cleavage divisions [8, 117]. The reproductive cell is focused not only on the synthesis of proteins that are required for photosynthesis but also of those for energy conversion by glycolysis, as was apparent in the group of the most highly expressed genes in this cell type (Fig. 6b). In somatic cells, photosynthesis and glycolysis-related genes were also among the most highly expressed genes (Fig. 6a). Even if somatic cells do not grow and divide, they have other energy-intense functions that are not relevant in reproductive cells, requiring sufficient energy to continuously build and restructure the surrounding ECM and to produce and operate the flagella. Fittingly, we also identified ECM- and flagella-related genes among the most highly expressed genes (Fig. 6a).

The examination of differential gene expression revealed that more than half of all genes showed a clear difference in expression between somatic and reproductive cells. More specifically, 7691 of 14,203 genes (54%) exhibited a fold difference in expression of 2 or more and a significance value of less than 0.05, and are therefore considered to be differentially expressed; this large proportion demonstrates an extensive compartmentalization of the transcriptome between cell types. Even though we analyzed the developmental stage prior to the onset of divisions, the group of the most overexpressed genes in reproductive cells (compared to somatic cells) contained large shares of genes related to cell division and gene regulation, respectively (Fig. 8b). Shortly before the beginning of cell divisions, reproductive cells appear to have begun with the synthesis of cleavage-related compounds to allow for subsequent rapid divisions. Initiation of mitosis, for example, requires severe regulation through a network of regulators [118, 119]. Moreover, expression of the corresponding genes obviously needs to be highly cell type specific (Fig. 8b). The fact that there are many more genes with greater than 30-fold overexpression in reproductive cells compared to somatic cells, rather than vice versa (Figs. 3 and 7), might indicate that highly cell type-specific gene expression is more important for reproductive cells than for somatic cells, particularly with regards to pivotal processes with high requirements of regulation such as mitosis. However, expression of such genes does not need to be exceptionally strong because they do not appear within the most highly expressed genes of reproductive cells (Fig. 6b). The group of the most overexpressed genes in somatic cells contains a considerable number of genes that are flagella associated (Fig. 8a), which is hardly surprising since their expression is required for the biogenesis, turnover, and operation of flagella and only somatic cells develop flagella [120]. Nevertheless, some flagellar proteins are also involved in other processes (e.g., transport), which are relevant for both cell types.

In the group of the most overexpressed genes in somatic cells, genes coding for ECM compounds were also prominent (Fig. 6a), which is as expected considering that somatic cells are known to secrete large amounts of ECM during ontogenesis, causing cells to move apart from neighboring cells and a rapid organism size growth [29, 72]. Once the process is complete, the volume of the ECM constitutes approximately 99% of the sphere. The complex ECM environment is considered an essential feature required for the evolutionary transition to multicellular volvocine algae [33, 35]. Previously, several ECM compounds were shown to be synthesized by somatic cells [43, 74, 79–81, 121–124]. It was previously assumed that reproductive cells, which lie below the somatic cell sheet, play only a minor role in ECM biosynthesis, if at all. However, in our analysis, it was surprising to find that, in the group of the most overexpressed genes in reproductive cells, the share of genes coding for ECM compounds was almost as large as that of such genes in somatic cells (Fig. 8a, b). Obviously, the involvement of reproductive cells in ECM biosynthesis is more important than previously thought. The reproductive cell is enclosed by a robust but thin ECM structure, termed the ‘(glycoprotein) vesicle’ (although it contains no membrane). This vesicle protects the reproductive cell and grows during embryogenesis until completion of the inversion [125]. The exact composition of the vesicle and the mechanism of its synthesis remain unclear, yet it is likely that the vesicle is synthesized by the reproductive cells. The fact that both cell types have a significant share of genes coding for ECM compounds within the group of the most overexpressed genes indicates that, even though both cell types contribute significantly to the components and functions of the ECM, these contributions are clearly distinct.

Among the ECM compounds of *Volvox* is a large family of well-known ECM proteins, the pherophorins. Like ECM biosynthesis in general, the synthesis of pherophorins was previously attributed to somatic cells alone [29, 72, 79–82]. The expression of some pherophorin genes was shown to be induced by both a sex-inducer, which triggers sexual development, and by mechanical wounding [82, 86, 126]; however, in our analysis, these genes were also mainly expressed by somatic cells, as expected. Nevertheless, we identified nine pherophorins within the top 100 most highly overexpressed genes in reproductive cells. Furthermore, we found pherophorins among the most overexpressed genes in both somatic cells and reproductive cells to approximately the same extent (Fig. 11). More specifically, we identified 97 pherophorin domains in total, with 44% of these being overexpressed in somatic cells and 40% in reproductive cells (the remaining 16% showed none or unclear cell type specificity). The clear overexpression of two-fifths of all pherophorins in reproductive cells was another surprising result. Concerning ECM biosynthesis, it appears therefore likely that reproductive cells account for more than the synthesis of the glycoprotein vesicle. Future studies need to assess the role of this large number of pherophorins expressed by reproductive cells.

In our search for protein domains with assigned molecular function we identified 9435 domains in 6216 genes, corresponding to 44% of all gene loci. Furthermore, we detected 15,254 domains involved in biological processes, accounting for 43% of all gene loci. Then, we sorted the protein domains into groups and subgroups separately for ‘molecular function’ and ‘biological process’, determined the percentage share of each group and subgroup, and analyzed each group or subgroup for cell type-specific overexpression (Figs. 9 and 10). However, it should be noted that the automated classification was not sufficient to obtain a precise and definite assignment of the domain functions of a particular gene product but was a helpful resource to obtain representative insights into gene expression patterns of some functional groups. An example is the group of genes containing gene products with electron carrier activity, which includes several components of the photosynthetic electron transport system (Fig. 11). The expression analysis demonstrated that 40% of these genes were overexpressed in reproductive cells, whereas only 16% were overexpressed in somatic cells, which correlates with the expectation that reproductive cells have a higher photosynthetic performance. Another interesting group with a larger share of domains overexpressed in reproductive cells compared to somatic cells was ‘antioxidant activity’ (Fig. 11). This group includes, among others, genes coding for peroxiredoxins, thioredoxins, glutathione reductases, and superoxide dismutases. The corresponding proteins are involved in redox signaling and serve as scavengers for reactive oxygen (ROS) and nitrogen species (RNS) [127, 128]. Given the higher photosynthetic performance, and subsequent production of ROS and RNS, of reproductive cells, ROS and RNS scavenging seems to be more important for reproductive cells than for somatic cells. Some peroxiredoxins have been shown to be directly involved in the protection of the photosynthetic apparatus [127], which is more abundant in reproductive cells. Furthermore, preventing ROS or RNS damage to DNA and the resulting mutations is more important in reproductive cells because they represent the germ line.

Conversely, there were groups with a larger share of domains overexpressed in somatic cells compared to reproductive cells. The biggest difference was seen in the group of photoreceptors (Fig. 11), which seems to reflect the fact that only somatic cells have an eyespot apparatus with a basic visual system [50, 100, 129, 130]. Further, there was also a large share of signal-transducer-activity domains and signaling domains overexpressed in somatic cells (Fig. 11). The reason for this could be that somatic cells have a broader range of cellular tasks compared to reproductive cells [8] and, therefore, may require a more complex signaling network.

A closer assessment was carried out for the group of transcription factors since control of gene transcription is a requirement for cell type-specific expression. More than half of all genes with transcription factor domains were shown to be differentially expressed with a fold difference in expression of 2 or more, whereby 32% of these domains were overexpressed in reproductive cells and 22% in somatic cells (Fig. 11). These large differences in the expression of transcription factor genes between the cell types reflects the strong differentiation of cell types despite *V. carteri* having only relatively recently diverged from its unicellular relatives [23, 25, 26].

## Conclusion

This study presents the first transcriptome-wide RNA-Seq analysis of separated cell types of *V. carteri* focusing on gene expression. Our expression dataset and the bioinformatic analyses provide a solid basis for further investigations of cell type-specific expression and its transcriptional regulation. Particularly, the expression analysis of potential key regulatory genes, like *rlsM* and other members of the *VARL* family, could promote the clarification of molecular mechanisms involved in the regulation of cellular differentiation. In addition, the large number of identified ECM-related genes with overexpression in reproductive cells was unexpected and should be subject to further studies.

## Methods

### Strain and culture conditions

The previously described female wild-type strain Eve10 of *V. carteri* f. *nagariensis* was used in this study [131]. Eve10 is a descendant of strains HK10 (female) and 69-1b (male) [117, 132], which originate from Japan. Cultures were grown synchronously under vegetative conditions in *Volvox* standard medium [117, 133]. Growth was performed at 28 °C in a cycle of 8 h dark/16 h cool fluorescent white light [134], at an average of ~100 μmol photons m^–2^ s^–1^ photosynthetically active radiation in glass tubes or Fernbach flasks. The glass tubes had caps that allowed for gas exchange and the Fernbach flasks were aerated with approximately 50 cm^3^ sterile air/min. To obtain three biological replicates for further processing (see below), three Fernbach flasks were inoculated with four Eve10 spheroids each and grown separately to a density of approximately 15 spheroids/mL each.

### Separation of cell types

The three separately grown and harvested cultures were kept separate. For each of these biological replicates the two cell types, i.e., somatic cells and reproductive cells, were separated mechanically from each other as previously described [49]. Briefly, *Volvox* spheroids were harvested from Fernbach flasks shortly before the onset of cell cleavage of reproductive cells (Fig. 1a, c). The spheroids were broken in a Dounce homogenizer and cell size-based separation of cell types was achieved by successive filtration on screens of different mesh sizes and a centrifugation step. The separated somatic cell sheets and reproductive cells were quickly checked microscopically for possible contamination with the other cell type (Fig. 1d, e). Immediately after the separation of cell types, cells were flash-frozen in liquid nitrogen and stored at –80 °C for subsequent RNA extraction.

### RNA isolation

Total RNA was extracted separately from both cell types of each of the three biological replicates using the phenol-based TRI Reagent (Sigma-Aldrich, St. Louis, MO) according to the manufacturer’s instructions and as previously described [100]. The extracted RNA was dissolved in RNase-free water and stored at −70 °C. The RNA concentration was adjusted to 100 ng/μL and quality was determined as described previously [92]. In addition, the quality of the cell type-specific RNA was re-checked using an Agilent 2100 Bioanalyzer (Agilent Technologies, Santa Clara, CA), which performs microfluidic electrophoretic separation on microfabricated chips [135]. All of our six RNA samples passed both quality controls. The OD 260/280 was 1.8–2.2 and the RNA integrity number value was ≥ 8.

### Whole transcriptome sequencing

The six samples with separately isolated total RNA of both cell types of each of the three biological replicates were prepared for RNA-Seq analysis. For each sample, 1 μg of total RNA was subjected to poly(A)^+^ RNA selection and mRNA fragmentation. This was followed by random primer cDNA synthesis and adapter ligation. Massively parallel sequencing was performed on an Illumina HiSeq2500 system with a read length of 50 bp. Purification of poly(A)^+^ RNA and sequencing of the six samples was done by GATC Biotech (Konstanz, Germany). With sequencing by synthesis technology, each base in a read was assigned a quality score (Q-score) by a Phred-like algorithm [136, 137]. Across all reads, 92% of the bases had quality scores above Q30, which indicates that the probability of an incorrect base call was less than 1 in 1000. The sequenced reads were quality filtered by applying the FASTX Toolkit [138], which is a collection of command line tools for short-read FASTA/FASTQ file preprocessing. Quality-based trimming was performed with the GenePattern Trimmomatic module using the following settings: LEADING:3, TRAILING:3, SLIDINGWINDOW:4:15, and MINLEN:36 [139]. All RNA-Seq data are available at the EMBL-EBI ArrayExpress repository under accession number E-MTAB-5691 [140–142].

### Mapping, data analysis, and bioinformatics

Quality filtered and trimmed sequence reads of all replicates of both cell types were mapped to the *V. carteri* f. *nagariensis* genome assembly v2 [16] using TopHat2 [143], with the following settings: tophat2 -r 300 -o $Folder --library-type fr-unstranded -p 8 $reference $forward1_R1.fastq,$forward2_R1.fastq $reverse1_R2.fastq,$reverse2_R2.fastq. Considering sequencing errors and the read length of 50 bp, up to two mismatches were allowed in mapping. Expression analysis and visualization was performed by using the short-read mapping analysis platform ReadXplorer 2.2.3 [52].

The ReadXplorer platform also includes the R package DESeq [54–56], which was used to normalize the count data and calculate mean values (baseMean, baseMeanA, baseMeanB), fold differences in expression, and *P* values (raw and adjusted) of a test for differential gene expression based on generalized linear models using negative binomial distribution errors. The baseMean is the mean normalized expression level of a given transcript, averaged over all replicates from all conditions; baseMeanA is the mean normalized expression level of a given transcript, averaged over the three biological replicates coming from reproductive cells (i.e., all condition A replicates); and baseMeanB is the mean normalized expression level of a given transcript, averaged over the three biological replicates coming from somatic cells (i.e., all condition B replicates) [54–56]. For quality control purposes, we also determined a minimum expression threshold. Therefore, we individually checked 100 expression profiles of genes that show an extremely low expression level, reflected by an extremely low baseMean value. We came to the assessment that a baseMean expression value of at least 12.5 is sufficient for robust expression analysis. As a consequence, we cannot make reliable statements regarding the expression of 999 predicted genes (7%) with a baseMean expression value below 12.5.

### Manual examination of gene structures on a random basis

The determination of gene structures is limited to genes with good coverage by mapped reads and therefore the expression needs to exceed a certain expression threshold. Thus, we individually checked 100 expression profiles of genes with low expression. We came to the assessment that genes with a baseMean expression value of at least 450 show sufficient coverage by mapped reads to estimate the correctness of gene structures. An example of a typical expression profile of such a gene with an expression value of 450 is shown in Additional file 7: Figure S2A. Based on this empirical threshold baseMean expression value of 450, we calculated that our RNA-Seq data provide a source for the quality control of exon-intron gene structures of at least 8893 out of the 14,247 predicted genes, which corresponds to 62% of all predicted genes. For comparison, we also checked expression profiles of genes with average and high expression individually. The average of all baseMean expression values in our dataset of 14,203 genes with mapped reads was 2822. An example of a typical expression profile of a gene with average expression is shown in Additional file 7: Figure S2B. Gene structures can be examined easily. The same applies, of course, to genes with high expression (Additional file 7: Figure S2C).

For quality control purposes, we examined the intron-exon structures of a series of genes on a random basis by using the threshold of 450. To this effect, we manually and systematically checked all expression profiles mapped to the first 1 million base pairs of the randomly chosen scaffold 9. The expression profiles allowed the identification of 103 genes in this section of the genome, 102 of which are known genes and one gene is new (Additional file 2: Table S1). Among the 103 genes are 66 genes with a baseMean expression value of at least 450, which enables estimation of correctness of the corresponding gene structures. The expression profiles revealed that 14 of these 66 genes show discrepancies within the coding sequence as compared to the *Volvox* gene predictions of gene annotation v2.1 on the Phytozome V12 platform, which represents 21% of the sufficiently expressed genes in this section of the genome (Additional file 2: Table S1). Moreover, 35 of the 66 sufficiently expressed genes show discrepancies within the UTRs (45%).

In addition to this part of scaffold 9, we performed the same analysis for 100 randomly selected gene loci from the complete list of all gene loci. For this purpose, we used the random number generator of random.org to randomly pick 100 gene loci out of the total list of 14,247 previously known gene loci (Additional file 3: Table S2). Among these 100 genes were 60 sufficiently expressed genes. The expression profiles revealed that 10 of these 60 sufficiently expressed genes showed discrepancies within the coding sequence as compared to the *Volvox* gene predictions according to gene annotation v2.1 on the Phytozome V12 platform, which represents 17% of the sufficiently expressed genes (Additional file 3: Table S2). Moreover, 25 of the 60 sufficiently expressed genes showed discrepancies within the UTRs (42%).

In summary, both the sample analysis of a genome fragment and the analysis of 100 randomly selected gene loci gave approximately the same percentage of discrepancies between the *Volvox* gene predictions according to gene annotation v2.1 on the Phytozome V12 platform and our expression profiles.

### Annotation

We obtained standard gene information of *V. carteri* f. *nagariensis* from the Phytozome V12 platform [53], including Pfam [102–104], GO [108, 109], and PANTHER annotation data [105–107]. The *V. carteri* gene information of Phytozome V12 came originally from the US Department of Energy's Joint Genome Institute annotation v2.1 of *V. carteri* genome assembly v2 [16]. Genes were screened for the corresponding identifiers, the identifiers were assigned to higher level GO-terms, and genes were sorted into groups of molecular function and biological process using QuickGO [114, 115]. In gene subsets of particular importance (e.g., 50 most abundant transcripts per cell type), we re-checked the contained gene products without annotation by using the BLASTP algorithm to screen all non-redundant protein sequences in the National Center for Biotechnology Information protein databases for sequence similarities [110–112]. If a gene product without previous annotation was found to be similar to sequences with assigned function, and if the e-value was less than 1 × 10^–10^, the same function was assigned to this gene product.

## Additional files

Additional file 1: Figure S1. Complete life cycle of *V. carteri*. (PDF 5372 kb)

Additional file 2: Table S1. Examples for discrepancies between gene predictions according to *V. carteri* genome annotation v2.1 on the Phytozome V12 platform and our expression profiles: examination of all expression profiles mapped to the first 1 million base pairs of the randomly chosen scaffold 9. (PDF 30 kb)

Additional file 3: Table S2. Examples for discrepancies between gene predictions according to *V. carteri* genome annotation v2.1 on the Phytozome V12 platform and our expression profiles: examination of 100 randomly chosen gene loci using the random number generator of random.org. (PDF 32 kb)

Additional file 4: Table S3. Cell type-specific gene expression, further information and references of 376 *Volvox* genes that show at least a brief mention in the literature. (PDF 116 kb)

Additional file 5: Table S4. Functional enrichment analysis of the most highly expressed genes in somatic cells, reproductive cells, and in total. (PDF 42 kb)

Additional file 6: Table S5. Functional enrichment analysis of the most overexpressed genes of both cell types. (PDF 54 kb)

Additional file 7: Figure S2. Examples of typical expression profiles of genes with low, average, and high expression. (PDF 236 kb)

## Abbreviations

BLASTP: basic local alignment search tool for protein-protein searches
cDNA: complementary deoxyribonucleic acid
ECM: extracellular matrix
GO: gene ontology (database)
MA-plot: plot of log-intensity ratios (M-values) versus log-intensity averages (A-values)
mRNA: messenger ribonucleic acid
PANTHER: protein analysis through evolutionary relationships (database)
Pfam: protein families (database)
RNA-Seq: ribonucleic acid sequencing
RNS: reactive nitrogen species
ROS: reactive oxygen species
