# Supplementary material for: Whole transcriptome RNA-Seq analysis reveals extensive cell type-specific compartmentalization in Volvox carteri
Source: BMC Biol. 2017 Nov 28;15:111. doi: 10.1186/s12915-017-0450-y (PMC5704591; doi:10.1186/s12915-017-0450-y)
Supplement: Supplementary file 1 — Complete life cycle of V. carteri. (PDF 5372 kb) [file 12915_2017_450_MOESM1_ESM.pdf]

Additional file 1: Figure S1. Complete life cycle of *V. carteri*.

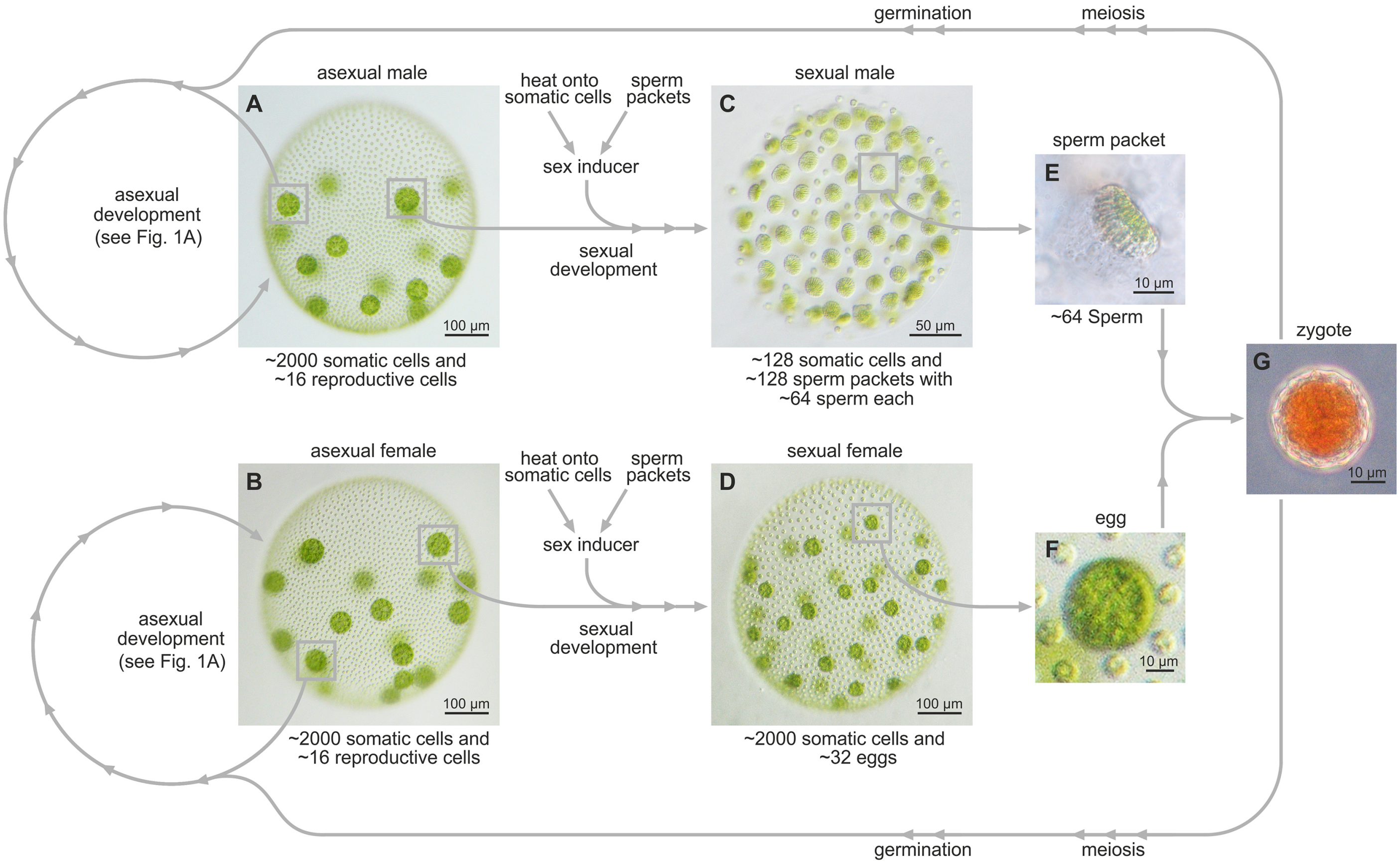

In nature, *V. carteri* lives in freshwater ponds, puddles and ditches. *Volvox* algae exist as distinct males and females, which can reproduce both asexually and sexually (Hallmann 2011; Hallmann et al. 1998; Kirk 1998). *V. carteri* reproduces asexually as long as the conditions are favorable and during this asexual development the males (**A**) look just like the females (**B**). For details of the asexual development see Fig. 1A. However, when their habitat begins to dry out, e.g., in the heat of late summer, they switch to the sexual reproduction. Asexual *Volvox* algae would die quickly once the habitat dried out but zygotes survive the drought and also the upcoming winter. Actually, the sexual development is initiated by heat that causes the somatic cells of the asexual *Volvox* spheroids to produce a highly efficient 32-kDa sex-inducing glycoprotein, the sex inducer (Hallmann et al. 1998; Kirk and Kirk 1986; Mages et al. 1988; Starr 1970; Starr and Jaenicke 1974; Tschochner et al. 1987). When the sex inducer is present, the reproductive cells of both sexes undergo a modified embryogenesis, which is also different in males and females. In males, embryonic cleavage divisions (including asymmetric cell division) result in juveniles with somatic cells and sperm packets in a 1:1 ratio (**C**). In females, juveniles with ~32 eggs arise (**D**). Mature sperm packets (**E**) are released and swim around until they contact a sexual female with mature eggs. The sperm packets also cause an amplification effect regarding induction of sexual development because they produce and liberate large amounts of sex inducer, which triggers

induction of sexual development in all the other *V. carteri* algae of the surrounding water area. As soon as the sperm packets come upon a female with mature eggs, the sperm cells penetrate the extracellular matrix of the female and fertilize the eggs (**F**). The resulting diploid zygotes (**G**) develop an orange coloration and secrete a thick, crenellated cell wall, which enables them to survive even if their habitat dries out completely and the cold of winter comes. The dormant zygote is the only diploid stage in the otherwise haploid life-cycle of *Volvox*. As soon as favorable conditions return in next spring and rain fills their habitat, the zygotes undergo meiosis and germination. The developing embryos then perform an asexual development. Each zygote either produces a single haploid female or a single haploid male.

References

Hallmann A (2011) Evolution of reproductive development in the volvocine algae. Sex. Plant Reprod. 24: 97-112

Hallmann A, Godl K, Wenzl S, Sumper M (1998) The highly efficient sex-inducing pheromone system of *Volvox*. Trends Microbiol. 6: 185-189

Kirk DL (1998) *Volvox*: molecular-genetic origins of multicellularity and cellular differentiation. Cambridge University Press, Cambridge

Kirk DL, Kirk MM (1986) Heat shock elicits production of sexual inducer in *Volvox*. Science 231: 51-54

Mages H-W, Tschochner H, Sumper M (1988) The sexual inducer of *Volvox carteri*. Primary structure deduced from cDNA sequence. FEBS Lett. 234: 407-410

Starr RC (1970) Control of differentiation in *Volvox*. Dev. Biol. Suppl. 4: 59-100

Starr RC, Jaenicke L (1974) Purification and characterization of the hormone initiating sexual morphogenesis in *Volvox carteri* f. *nagariensis* Iyengar. Proc Natl Acad Sci U S A 71: 1050-1054

Tschochner H, Lottspeich F, Sumper M (1987) The sexual inducer of *Volvox carteri*: purification, chemical characterization and identification of its gene. EMBO J. 6: 2203-2207
