# Supplementary material for: Whole transcriptome RNA-Seq analysis reveals extensive cell type-specific compartmentalization in Volvox carteri
Source: BMC Biol. 2017 Nov 28;15:111. doi: 10.1186/s12915-017-0450-y (PMC5704591; doi:10.1186/s12915-017-0450-y)
Supplement: Supplementary file 2 — Examples for discrepancies between gene predictions according to V. carteri genome annotation v2.1 on the Phytozome V12 platform and our expression profiles: examination of all expression profiles mapped to the first 1 million base pairs of the randomly chosen scaffold 9. (PDF 30 kb) [file 12915_2017_450_MOESM2_ESM.pdf]

Additional file 2: Table S1. Examples for discrepancies between gene predictions according to *V. carteri* genome annotation v2.1 on the Phytozome V12 platform and our expression profiles: examination of all expression profiles mapped to the first 1 million base pairs of the randomly chosen scaffold 9.

examination of the first 1 million base pairs of scaffold 9 - overview

determined total number of genes:	103	
previously predicted genes (annotation v2.1):	102	
identified new genes:	1	
genes with baseMean expression value <450:	37	i.e., expression not sufficient to estimate the correctness of gene structures
genes with baseMean expression value >450:	66	i.e., expression valule allows for the estimation of the correctness of gene structures

33 of 66 without detectable discrepancies (50%)

33 of 66 with detectable discrepancies (50%)

- 14 of 66 with discrepancies within the coding sequence (21%)
- 30 of 66 with discrepancies within the UTR (45%)

examination of the first 1 million base pairs of scaffold 9 - details

					identified discrepancies between expression profiles and previous gene predictions according to *V. carteri* genome annotation v2.1					
					discrepancies within coding sequence				discrepancies within UTR	
locusName	baseMean	baseMean reproductive cells	baseMean somatic cells	baseMean < 450? (if so, then no statement)	exon longer than predicted	exon shorter than predicted	additional, unpredicted exon	artificial exon (no reads)	discrepancies within 5'-UTR	discrepancies within 3'-UTR
Vocar.0009s0001	1052	820	1284		X					
Vocar.0009s0002	22	35	8	X						
Vocar.0009s0003	134	209	60	X						
Vocar.0009s0004	173	273	74	X						
Vocar.0009s0005	277	375	179	X						
Vocar.0009s0006	1639	956	2322						X	
Vocar.0009s0007	760	311	1209		X					X
Vocar.0009s0008	75	102	49	X						
Vocar.0009s0009	5687	3017	8357							
Vocar.0009s0010	2157	699	3614			X		X	X	X
Vocar.0009s0011	4898	4020	5776							X
Vocar.0009s0012	511	354	668				X			X
Vocar.0009s0013	1867	1917	1818							X
Vocar.0009s0014	129	209	49	X						
Vocar.0009s0015	1352	1266	1438							X
Vocar.0009s0016	1456	1073	1838							
Vocar.0009s0017	19346	23593	15099							
Vocar.0009s0018	69722	61508	77937						X	
Vocar.0009s0019	126	174	79	X						
Vocar.0009s0020	399	585	213							
Vocar.0009s0021	806	118	1494		X		X		X	X
Vocar.0009s0022	963	963	963							
Vocar.0009s0023	2021	2568	1473							
Vocar.0009s0024	1822	2474	1169		X					
Vocar.0009s0025	923	503	1343							

Vocar.0009s0026	205	331	80	X						
Vocar.0009s0027	270	378	161	X						
Vocar.0009s0028	176	277	75	X						
Vocar.0009s0029	2681	2157	3205							
Vocar.0009s0030	3442	3521	3364		X				X	
Vocar.0009s0031	3421	4851	1990							
Vocar.0009s0032	2240	1811	2668							X
Vocar.0009s0033	2272	1508	3036							
Vocar.0009s0034	545	619	471							
Vocar.0009s0035	5160	3001	7318							
Vocar.0009s0036	115	129	100	X						
Vocar.0009s0037	588	140	1036							
Vocar.0009s0038	20	36	3	X						
Vocar.0009s0039	7	3	10	X						
Vocar.0009s0040	12066	2093	22038							
Vocar.0009s0041	721	112	1331							X
Vocar.0009s0042	1329	804	1855							X
Vocar.0009s0043	735	1034	437		X				X	X
Vocar.0009s0044	80	133	28	X						
Vocar.0009s0045	302	405	199	X						
Vocar.0009s0046	817	848	787							X
Vocar.0009s0047	11280	1676	20884							
Vocar.0009s0048	2	4	0	X						
Vocar.0009s0049	298	531	64							
Vocar.0009s0050	14	27	1	X						
Vocar.0009s0051	897	516	1278							
Vocar.0009s0052	93	166	19	X						
Vocar.0009s0053	68165	61178	75152							X
Vocar.0009s0054	782	819	744							
Vocar.0009s0055	20	33	7	X						
Vocar.0009s0056	11569	14941	8198							
Vocar.0009s0057	1736	1250	2223							
Vocar.0009s0058	981	1816	145							X
Vocar.0009s0059	656	1162	151							X
Vocar.0009s0060	484	832	135							X
Vocar.0009s0061	2246	2071	2421							X
Vocar.0009s0062	257	284	230	X						
Vocar.0009s0063	522	365	678							
Vocar.0009s0064	66	33	100	X						
Vocar.0009s0065	530	438	622							
Vocar.0009s0066	491	608	374							
Vocar.0009s0067	820	467	1173							
Vocar.0009s0068	49897	57244	42549						X	
Vocar.0009s0069	1793	1012	2574							
Vocar.0009s0070	143	183	103	X						
Vocar.0009s0071	954	792	1116		X				X	X
Vocar.0009s0072	87	66	108	X						
Vocar.0009s0073	586	1002	170							

Vocar.0009s0074	1018	555	1482				X		X	X
Vocar.0009s0075	630	525	735			X				
Vocar.0009s0076	2576	317	4835							
Vocar.0009s0077	35	30	40	X						
Vocar.0009s0078	5561	4445	6676							
Vocar.0009s0079	71	15	127	X						
Vocar.0009s0080	1097	1338	855		X	X	X	X	X	
Vocar.0009s0081	1731	214	3248							
Vocar.0009s0082	7	2	11	X						
Vocar.0009s0083	1909	266	3553							
Vocar.0009s0084	1457	1383	1532							
Vocar.0009s0085	3	1	5	X						
Vocar.0009s0086	252	483	20		X			X	X	X
Vocar.0009s0087	3	4	1	X						
Vocar.0009s0088	199	164	234	X						
Vocar.0009s0089	1697	1756	1638							X
Vocar.0009s0090	495	215	774							X
Vocar.0009s0091	181	155	207	X						
Vocar.0009s0092	814	838	789							
Vocar.0009s0093	306	363	250	X						
Vocar.0009s0094	852	608	1095		X	X	X	X	X	X
Vocar.0009s0095	89	143	36	X						
Vocar.0009s0096	1	0	1	X						
Vocar.0009s0097	23	39	7	X						
Vocar.0009s0098	860	868	852						X	
Vocar.0009s0099	12	9	15	X						
new gene	200			X						
Vocar.0009s0100	6	8	5	X						
Vocar.0009s0101	706	600	811							X
Vocar.0009s0102	35587	3419	67756							
