# Supplementary material for: Whole transcriptome RNA-Seq analysis reveals extensive cell type-specific compartmentalization in Volvox carteri
Source: BMC Biol. 2017 Nov 28;15:111. doi: 10.1186/s12915-017-0450-y (PMC5704591; doi:10.1186/s12915-017-0450-y)
Supplement: Supplementary file 3 — Examples for discrepancies between gene predictions according to V. carteri genome annotation v2.1 on the Phytozome V12 platform and our expression profiles: examination of 100 randomly chosen gene loci using the random number generator of random.org. (PDF 32 kb) [file 12915_2017_450_MOESM3_ESM.pdf]

Additional file 3: Table S2. Examples for discrepancies between gene predictions according to *V. carteri* genome annotation v2.1 on the Phytozome V12 platform and our expression profiles: examination of 100 randomly chosen gene loci using the random number generator of random.org.

examination of 100 randomly chosen gene loci - overview

determined total number of genes:	100	
previously predicted genes (annotation v2.1):	100	
identified new genes:	-	(it is not possible to identify new genes with this strategy)
genes with baseMean expression value <450:	40	i.e., expression not sufficient to estimate the correctness of gene structures
genes with baseMean expression value >450:	60	i.e., expression valule allows for the estimation of the correctness of gene structures

31 of 60 without detectable discrepancies (52%)

29 of 60 with detectable discrepancies (48%)

- 10 of 60 with discrepancies within the coding sequence (17%)
- 25 of 60 with discrepancies within the UTR (42%)

examination of 100 randomly chosen gene loci - details

						**identified discrepancies between expression profiles and previous gene predictions according to *V. carteri* genome annotation v2.1**					
						**discrepancies within coding sequence**				**discrepancies within UTR**	
**randomly chosen number to pick 100 gene loci out of the total list with 14,247 gene loci**	**locusName**	**baseMean**	**baseMean reproductive cells**	**baseMean somatic cells**	**baseMean < 450? (if so, then no statement)**	**exon longer than predicted**	**exon shorter than predicted**	**additional, unpredicted exon**	**artificial exon (no reads)**	**discrepancies within 5'-UTR**	**discrepancies within 3'-UTR**
12002	Vocar.0039s0046	40	55	24	X						
10529	Vocar.0029s0054	161	281	41	X						
12779	Vocar.0048s0023	48044	46697	49391							
6322	Vocar.0012s0145	26621	35980	17261							
1174	Vocar.0001s1175	293	487	99							
4378	Vocar.0006s0338	478	613	344							
743	Vocar.0001s0744	244	140	349	X						
11332	Vocar.0033s0170	1139	341	1937				X			
858	Vocar.0001s0859	23	21	26	X						
12119	Vocar.0040s0068	3529	742	6316							X
2144	Vocar.0002s0336	2515	3245	1785							
10229	Vocar.0027s0145	2438	2336	2540							
1306	Vocar.0001s1307	244	369	118	X						
3893	Vocar.0005s0364	149	264	35	X						
11605	Vocar.0036s0087	302	374	230	X						
7291	Vocar.0015s0207	975	1148	803							X
13537	Vocar.0064s0020	85	130	39	X						
11477	Vocar.0029s0002	205	68	342	X						
12171	Vocar.0041s0012	1017	1267	766							X
963	Vocar.0001s0964	59	90	27	X						
13057	Vocar.0053s0042	458	787	129							X
4803	Vocar.0007s0288	1353	960	1745							
6422	Vocar.0012s0245	29	53	6	X						
3633	Vocar.0005s0103	1559	117	3002		X		X		X	X
8184	Vocar.0018s0173	75	140	11	X						

5044	Vocar.0008s0065	98	181	14	X						
6365	Vocar.0012s0188	3753	1855	5652							
9310	Vocar.0023s0107	1	3	0	X						
8961	Vocar.0021s0196	8795	872	16719							X
9064	Vocar.0022s0089	44	58	30	X						
1580	Vocar.0001s1581	84	120	49	X						
9902	Vocar.0026s0004	259	376	143	X						
1448	Vocar.0001s1449	8534	1027	16041							
5685	Vocar.0009s0245	9	8	10	X						
12999	Vocar.0052s0031	7876	5554	10198							
5602	Vocar.0009s0162	542	1017	67					X	X	X
6575	Vocar.0013s0085	1080	1713	447							
2087	Vocar.0002s0279	502	397	607				X			X
12576	Vocar.0045s0022	167	185	150	X						
1775	Vocar.0001s1777	1161	1842	479							
2825	Vocar.0003s0337	144	143	144	X						
7014	Vocar.0014s0223	27654	24759	30548							
13603	Vocar.0066s0016	99	187	11	X						
2044	Vocar.0002s0236	629	405	852						X	X
9153	Vocar.0022s0178	151	251	51	X						
670	Vocar.0001s0671	1204	285	2122							
3218	Vocar.0004s0213	928	1558	297							
13166	Vocar.0055s0024	13	8	19	X						
10662	Vocar.0029s0187	691	836	545		X					
4023	Vocar.0005s0495	132	91	173	X						
4049	Vocar.0006s0008	242	323	161	X						
10777	Vocar.0030s0109	3181	3123	3239						X	X
4609	Vocar.0007s0094	130	176	84	X						
11903	Vocar.0038s0005	42	17	68	X						
5820	Vocar.0009s0381	127	30	225	X						
12849	Vocar.0049s0042	1162	1741	584							X
6904	Vocar.0014s0113	232	239	226	X						
11206	Vocar.0033s0044	2711	3094	2328							
2504	Vocar.0003s0015	2020	718	3322							X
6442	Vocar.0012s0265	883	1074	692				X			X
10036	Vocar.0026s0139	710	1158	262							
11902	Vocar.0038s0004	1066	1124	1008							X
1351	Vocar.0001s1352	149	237	61	X						
6815	Vocar.0014s0022	7874	967	14781							
5005	Vocar.0008s0026	3850	2350	5350							X
1692	Vocar.0001s1694	2943	407	5479						X	
2572	Vocar.0003s0083	4681	1228	8134		X					
8822	Vocar.0021s0057	770	1255	285						X	X
8222	Vocar.0018s0211	1091	872	1310							X
12291	Vocar.0042s0035	3	3	3	X						
6291	Vocar.0012s0114	4044	3396	4693							
5558	Vocar.0009s0118	308	561	56							
8511	Vocar.0019s0223	2578	1715	3441							

3086	Vocar.0004s0081	2	3	1	X						
11597	Vocar.0035s0137	175307	50327	300287							
2883	Vocar.0003s0395	1197	1120	1275							
12195	Vocar.0041s0036	541	872	210				X		X	X
6227	Vocar.0012s0050	587	372	802			X				
13441	Vocar.0062s0009	69	121	16	X						
13773	Vocar.0073s0022	1547	2718	376							X
10495	Vocar.0029s0020	3720	2641	4799							
4380	Vocar.0006s0340	150	251	50	X						
5327	Vocar.0008s0348	2437	2906	1968							
1218	Vocar.0001s1219	2418	1841	2995							X
2512	Vocar.0003s0023	7118	13982	255							
10402	Vocar.0028s0119	770	1150	390							
9005	Vocar.0022s0030	1301	649	1952						X	X
6589	Vocar.0013s0099	1	1	1	X						
4520	Vocar.0007s0005	3667	2101	5233							
2002	Vocar.0002s0194	6441	1064	11819							
13027	Vocar.0053s0012	39	58	20	X						
12091	Vocar.0040s0040	1847	2342	1353							
2578	Vocar.0003s0089	142	265	19	X						
11169	Vocar.0033s0007	905	885	924						X	X
12459	Vocar.0043s0075	22	20	24	X						
5679	Vocar.0009s0239	5	9	0	X						
1891	Vocar.0002s0082	1195	824	1566				X			X
7898	Vocar.0017s0105	419	471	367							X
5488	Vocar.0009s0048	2	4	0	X						
6987	Vocar.0014s0196	572	440	705							
