# Supplementary material for: Whole transcriptome RNA-Seq analysis reveals extensive cell type-specific compartmentalization in Volvox carteri
Source: BMC Biol. 2017 Nov 28;15:111. doi: 10.1186/s12915-017-0450-y (PMC5704591; doi:10.1186/s12915-017-0450-y)
Supplement: Supplementary file 4 — Cell type-specific gene expression, further information and references of 376 Volvox genes that show at least a brief mention in the literature. (PDF 116 kb) [file 12915_2017_450_MOESM4_ESM.pdf]

Additional file 4: Table S3. Cell-type specific gene expression, further information and references of 376 *Volvox* genes that show at least a brief mention in literature.

		data of this publicaton								previously published information				
gene name	ID (Volvox v2.1)	baseMean	baseMean reproductive cells	baseMean somatic cells	p adjusted	fold difference (somatic cells/ reproductive cells)	fold difference (reproductive cells/ somatic cells)	overexpressed in somatic cells	overexpressed in reproductive cells	fold difference (somatic cells/ reproductive cells)	fold difference (reproductive cells/ somatic cells)	overexpressed in somatic cells	overexpressed in reproductive cells	references
**Genes involved in major morphological and developmental traits according to Olson and Nedelcu 2016:**														
regA	Vocar.0021s0042	2575	434	4716	0.000	10.87	0.09	X		149.70	0.01	X		Harper et al. 1987, Kirk et al. 1987, Kirk et al. 1999, Meissner et al. 1999, Stark et al. 2001, Babinger et al.
rlsA	Vocar.0021s0041	55	35	74	0.150	2.11	0.47	(X)		69.32	0.01	X		Olson and Nedelcu 2016, Duncan et al. 2007, Nematollahi et al. 2006
rlsb	Vocar.0021s0043	99	27	171	0.000	6.33	0.16	X						Olson and Nedelcu 2016, Duncan et al. 2007
rlsc	Vocar.0021s0045	16	9	23	0.160	2.56	0.39	(X)						Olson and Nedelcu 2016, Duncan et al. 2007
rlsd	Vocar.0026s0121	1411	1166	1655	0.270	1.42	0.70							Olson and Nedelcu 2016, Duncan et al. 2007
rlse	Vocar.0003s0509	4813	2891	6735	0.000	2.33	0.43	X						Olson and Nedelcu 2016, Duncan et al. 2007
rlsF	Vocar.0008s0105	232	119	344	0.070	2.89	0.35	(X)						Olson and Nedelcu 2016, Duncan et al. 2007
rlsG	Vocar.0003s0512	1902	1646	2158	0.260	1.31	0.76							Olson and Nedelcu 2016, Duncan et al. 2007
rlsH	Vocar.0022s0042	274	107	440	0.000	4.11	0.24	X						Olson and Nedelcu 2016, Duncan et al. 2007
rlsI	Vocar.0020s0165	931	662	1200	0.080	1.81	0.55	(X)						Olson and Nedelcu 2016, Duncan et al. 2007
rlsJ	Vocar.0024s0052	870	911	829	0.660	0.91	1.10							Olson and Nedelcu 2016, Duncan et al. 2007
rlsK	Vocar.0024s0053	1444	1071	1817	0.030	1.70	0.59	(X)						Olson and Nedelcu 2016, Duncan et al. 2007
rlsL	Vocar.0001s1384	1935	1099	2771	0.000	2.52	0.40	X						Olson and Nedelcu 2016, Duncan et al. 2007
rlsM	Vocar.0025s0071	976	1693	259	0.000	0.15	6.54		X					Olson and Nedelcu 2016, Duncan et al. 2007

RBR1 (mat3)	Vocar.0038s0042	5656	3248	8064	0.000	2.48	0.40	X		9.00	0.11	X		Nematollahi et al. 2006, Kianianmomeni et al. 2008, van den Heuvel and Dyson 2008, Hallmann 2009, Ferris et al. 2010, Hiraide et al. 2013, Hanschen et al. 2016, Olson and Nedelcu 2016
cycd1.1	Vocar.0030s0189	13762	4709	22814	0.000	4.84	0.21	X						Olson and Nedelcu 2016, Prochnik et al. 2010
cycd1.2	Vocar.0040s0002	5	7	3	0.350	0.43	2.33							Olson and Nedelcu 2016, Prochnik et al. 2010
cycd1.3	Vocar.0040s0001	640	1084	195	0.000	0.18	5.56		X					Olson and Nedelcu 2016, Prochnik et al. 2010
cycd1.4	Vocar.0040s0003	587	841	333	0.000	0.40	2.53		X					Olson and Nedelcu 2016, Prochnik et al. 2010
glsA	Vocar.0024s0133	440	853	27	0.000	0.03	31.59		X					Kirk 1997, Miller and Kirk 1999, Kirk 2001, Cheng et al. 2003, Cheng et al. 2005, Pappas and Miller 2009, Olson and Nedelcu 2016
invA	Vocar.0016s0074	2313	2837	1788	0.080	0.63	1.59							Olson and Nedelcu 2016, Nishii et al. 2003
invB	Vocar.0031s0075	1316	1210	1422	0.680	1.18	0.85							Olson and Nedelcu 2016
invC	Vocar.0002s0555	1213	1155	1270	0.730	1.10	0.91							Olson and Nedelcu 2016
**Genes of ECM glycoproteins according to Hallmann 2003:**														
phI	Vocar.0004s0255	83001	7038	158963	0.000	22.59	0.04	X						Sumper et al. 1993, Godl et al. 1995, Sumper and Hallmann 1998, Hallmann 2003, Hallmann 2006, Prochnik et al. 2010
phII	85077 (Volvox v1.0)	48000	6000	90000	0.000	15.00	0.07	X						Sumper et al. 1993, Godl et al. 1995, Sumper and Hallmann 1998, Hallmann 2003, Hallmann 2006, Prochnik et al. 2010
phIII	83859 (Volvox v1.0)	4800	600	9000	0.000	15.00	0.07	X						Hallmann 2003, Godl et al. 1995
phS	104453 (Volvox v1.0)	4800	600	9200	0.000	15.33	0.07	X						Godl et al. 1997, Sumper and Hallmann 1998, Hallmann 2003, Hallmann 2006, Prochnik et al. 2010
ph DZ1	77905 (Volvox v1.0)	2200	400	4000	0.000	10.00	0.10	X						Hallmann 2003, Ender et al. 2002
ph DZ2	90188 (Volvox v1.0)	2200	400	4000	0.000	10.00	0.10	X						Hallmann 2003, Ender et al. 2002
Algal-CAM	Vocar.0039s0020	1265	2509	20	0.000	0.01	125.45		X					Huber and Sumper 1994, Sumper and Hallmann 1998, Hallmann 2003
VheA	Vocar.0001s0611	944	583	1304	0.000	2.24	0.45	X						Tam and Kirk 1991a, Tam and Kirk 1991b, Tam et al. 1991, Shimizu et al. 2002, Hallmann 2003, Fukada et al. 2006, Nishimura et al. 2017
ISG	104540 (Volvox v1.0)	11	20	1	0.000	0.05	20.00		X					Hallmann 2003

SSG185 = ssgA	Vocar.0002s0564	50529	7584	93473	0.000	12.33	0.08	X		46,00/ 10,00		X	Ertl et al. 1989, Holst et al. 1989, Tam and Kirk 1991a, Tam and Kirk 1991b, Tam et al. 1991, Mengele and Sumper 1992, Sumper and Hallmann 1998, Hallmann 2003, Nematollahi et al. 2006
DZ-HRGP	127246 (Volvox v1.0)	1500	500	2500	0.000	5.00	0.20	X					Hallmann 2003, Ender et al. 1999
Chi1	Vocar.0004s0398	200	20	380	0.030	19.00	0.05	X					Amon et al. 1998, Sumper and Hallmann 1998, Hallmann 2003
cprA	Vocar.0001s1730	14430	1382	27478	0.000	19.88	0.05	X					Amon et al. 1998, Sumper and Hallmann 1998, Hallmann 2003
LSG1	Vocar.0016s0019	17617	2540	32693	0.000	12.87	0.08	X					Hallmann 2003, Shimizu et al. 2002, Tam and Kirk 1991a, Tam and Kirk 1991b, Tam et al. 1991
LSG2	Vocar.0012s0179	5314	3140	7487	0.170	2.38	0.42						Hallmann 2003, Shimizu et al. 2002, Tam and Kirk 1991a, Tam and Kirk 1991b, Tam et al. 1991
VMP1 (S9)	Vocar.0007s0366	80292	9410	151173	0.000	16.07	0.06	X					Hallmann et al. 2001, Heitzer and Hallmann 2002, Shimizu et al. 2002, Hallmann 2003, Hanschen et al. 2016
VMP2	Vocar.0062s0024	2613	435	4791	0.000	11.01	0.09	X					Hallmann 2003, Heitzer and Hallmann 2002
VMP3	Vocar.0016s0064	125163	19926	230400	0.000	11.56	0.09	X					Hallmann et al. 2001, Heitzer and Hallmann 2002, Hallmann 2003, Hanschen et al. 2016
VMP4	Vocar.0002s0001	1646	238	3053	0.020	12.83	0.08	X					Hallmann 2003
Ars1	Vocar.0025s0060	7	3	11	0.130	3.67	0.27						Hallmann 2003
phoX	Vocar.0013s0281	201	381	20	0.000	0.05	19.05		X				Hallmann 1999, Hallmann 2003
**Reference genes investigated by Kianianmomeni et al. 2013:**													
cyn23	Vocar.0024s0252	9313	4506	14120	0.000	3.13	0.32	X					Kianianmomeni and Hallmann 2013
eef1	Vocar.0006s0271	25906	22641	29170	0.300	1.29	0.78						Kianianmomeni and Hallmann 2013
gap3	Vocar.0013s0021	371936	428603	315268	0.490	0.74	1.36						Kianianmomeni and Hallmann 2013
rlp8	Vocar.0043s0070	68880	71703	66056	0.700	0.92	1.09						Kianianmomeni and Hallmann 2013
rlp23	Vocar.0002s0021	35997	35724	36269	0.960	1.02	0.98						Kianianmomeni and Hallmann 2013, Kianianmomeni and Hallmann 2015
tbpA	Vocar.0016s0293	2445	2626	2264	0.590	0.86	1.16						Kianianmomeni and Hallmann 2013
tubA1	Vocar.0035s0137	175307	50327	300287	0.000	5.97	0.17	X					Mages et al. 1988, Mages et al. 1995, Kianianmomeni and Hallmann 2013, Hanschen et al. 2016
tubB1	Vocar.0007s0110	135505	34555	236455	0.000	6.84	0.15	X					Harper and Mages 1988, Mages et al. 1995, Kianianmomeni and Hallmann 2013, Hanschen et al. 2016

18S	FJ610144 (GenBank)	7500	5000	10000	0.000	2.00	0.50	X						Kianianmomeni and Hallmann 2013
act	Vocar.0043s0003	40985	37466	44504	0.590	1.19	0.84							Kianianmomeni and Hallmann 2013
**Genes that were investigated by real-time RT-PCR in Nematollahi et al. 2006:**														
actA	Vocar.0043s0003	40985	37466	44504	0.590	1.19	0.84							Nematollahi et al. 2006, Kianianmomeni and Hallmann 2013
SSG185 = ssgA	Vocar.0002s0564	50529	7584	93473	0.000	12.33	0.08	X		46,00/ 10,00		X		Ertl et al. 1989, Holst et al. 1989, Tam and Kirk 1991a, Tam and Kirk 1991b, Tam et al. 1991, Mengele and Sumper 1992, Sumper and Hallmann 1998, Hallmann 2003, Nematollahi et al. 2006
regA	Vocar.0021s0042	2575	434	4716	0.000	10.87	0.09	X		149.70	0.01	X		Harper et al. 1987, Kirk et al. 1987, Kirk et al. 1999, Meissner et al. 1999, Stark et al. 2001, Babinger et al. 2006, Nematollahi et al. 2006, Duncan et al. 2006, Duncan et al. 2007, Olson and Nedelcu 2016, Matt and Umen 2016
gon30	Vocar.0030s0054	13491	24055	2927	0.000	0.12	8.22		X	0.15	6.62		X	Nematollahi et al. 2006
gon167	Vocar.0001s1685	82	149	15	0.000	0.10	9.93		X	0.17	5.80		X	Tam and Kirk 1991a, Tam and Kirk 1991b, Tam et al. 1991, Nematollahi et al. 2006
rlsA	Vocar.0021s0041	55	35	74	0.150	2.11	0.47	(X)		69.32	0.01	X		Nematollahi et al. 2006, Duncan et al. 2007
csrp1	Vocar.0017s0201	21493	18305	24681	0.240	1.35	0.74	(X)		3.74	0.27	X		Nematollahi et al. 2006
ard1	Vocar.0001s0162	2191	1998	2384	0.430	1.19	0.84			0.44	2.27		X	Nematollahi et al. 2006
mrp2	Vocar.0012s0088	31630	58577	4682	0.000	0.08	12.51		X	16.45	0.06	X		Nematollahi et al. 2006
gspk47	Vocar.0007s0112	3955	7138	771	0.010	0.11	9.26		X	0.57	1.75			Nematollahi et al. 2006
nitA	Vocar.0008s0140	2166	4203	129	0.000	0.03	32.58		X	1.36	0.74			Nematollahi et al. 2006
dyhA	Vocar.0014s0194	23304	2755	43853	0.000	15.92	0.06	X		159.80	0.01	X		Mitchell and Brown 1994, Porter et al. 1996, Mitchell and Brown 1997, Myster et al 1997, Yagi et al. 2005, Nematollahi et al. 2006
klpA	Vocar.0006s0438	12490	6036	18943	0.000	3.14	0.32	X		21.40	0.05	X		Nematollahi et al. 2006
fer1	Vocar.0002s0012	16539	32201	876	0.000	0.03	36.76		X	0.13	7.70		X	Nematollahi et al. 2006
nab1	Vocar.0002s0155	52607	63357	41856	0.350	0.66	1.51		(X)	0.24	4.10		X	Nematollahi et al. 2006
rap41	Vocar.0001s1288	1904	3624	183	0.000	0.05	19.80		X	0.20	5.00		X	Nematollahi et al. 2006
fbp1	Vocar.0016s0204	20485	27081	13889	0.190	0.51	1.95			0.63	1.60			Nematollahi et al. 2006
cp12	Vocar.0042s0039	140328	185149	95506	0.110	0.52	1.94		(X)	0.25	4.00		X	Nematollahi et al. 2006

prfA	Vocar.0005s0310	5158	8560	1756	0.000	0.21	4.87		X	0.19	5.30		X	Nematollahi et al. 2006
fsd1	Vocar.0057s0039	16231	29401	3061	0.000	0.10	9.61		X	0.30	3.30		X	Nematollahi et al. 2006
rpl37	Vocar.0005s0266	32859	31626	34091	0.750	1.08	0.93			0.14	7.40		X	Nematollahi et al. 2006
glu1	Vocar.0006s0290	2521	4543	499	0.000	0.11	9.10		X	1.70	0.59			Nematollahi et al. 2006
hsp70B	Vocar.0038s0026	7987	11233	4740	0.000	0.42	2.37		X	0.77	1.30			Nematollahi et al. 2006
hsp40A	Vocar.0001s1052	2712	2548	2875	0.660	1.13	0.89			6.50	0.15	X		Nematollahi et al. 2006
ubcA	Vocar.0007s0147	2609	3078	2139	0.220	0.69	1.44			4.40	0.23	X		Nematollahi et al. 2006
ponA	Vocar.0046s0033	2727	1880	3573	0.010	1.90	0.53			1.90	0.53			Nematollahi et al. 2006
RBR1 (mat3)	Vocar.0038s0042	5656	3248	8064	0.000	2.48	0.40	X		9.00	0.11	X		Nematollahi et al. 2006, Kianianmomeni et al. 2008, van den Heuvel and Dyson 2008, Hallmann 2009, Ferris et al. 2010, Hiraide et al. 2013, Hanschen et al. 2016, Olson and Nedelcu 2016
vpeA	Vocar.0031s0120	282	298	265	0.880	0.89	1.12			1.60	0.63			Nematollahi et al. 2006
sac1	Vocar.0018s0012	685	1037	333	0.000	0.32	3.11		X	2.00	0.50	X		Nematollahi et al. 2006
rcd1	Vocar.0007s0052	1287	1659	915	0.020	0.55	1.81			0.19	5.20		X	Nematollahi et al. 2006
adcA	Vocar.0203s0001	1983	387	3578	0.000	9.25	0.11	X		22.20	0.05	X		Nematollahi et al. 2006
nipA	Vocar.0033s0037	9548	5947	13148	0.000	2.21	0.45	X		3.40	0.29	X		Nematollahi et al. 2006
lciB	Vocar.0008s0028	2981	2995	2966	0.880	0.99	1.01			4.40	0.23	X		Nematollahi et al. 2006
upf1	Vocar.0036s0096	248	331	164	0.020	0.50	2.02		X	1.90	0.53			Nematollahi et al. 2006
upf2	Vocar.0031s0107	5170	4839	5501	0.570	1.14	0.88			0.71	1.40			Nematollahi et al. 2006
upf3	Vocar.0003s0179	24257	34284	14230	0.010	0.42	2.41		X	1.60	0.63			Nematollahi et al. 2006
upf4	Vocar.0001s1288	1904	3624	183	0.000	0.05	19.80		X	0.23	4.40		X	Nematollahi et al. 2006
upf5	Vocar.0004s0384	9616	11231	8000	0.470	0.71	1.40			2.50	0.40	X		Nematollahi et al. 2006
upf6	Vocar.0031s0009	34621	23660	45581	0.030	1.93	0.52	(X)		6.80	0.15	X		Nematollahi et al. 2006
**Genes of matrix metalloproteases according to Hanschen et al. 2016:**														
VMP31	EFJ42898 (GenBank)	0	0	0	---	---	---							Hanschen et al. 2016
[without name]	Vocar.0001s1363	391	61	720	0.000	11.80	0.08	X						Hanschen et al. 2016
VMP35	41924 (Volvox v1.0)	50	25	75	---	3.00	0.33							Hanschen et al. 2016

VMP36	56439 (Volvox v1.0)	14	2	25	---	12.50	0.08		Hanschen et al. 2016
[without name]	Vocar.0028s0115	11	18	3	0.380	0.17	6.00		Hanschen et al. 2016
VMP8	Vocar.0016s0066	10385	1471	19298	0.000	13.12	0.08	X	Hanschen et al. 2016
VMP9	Vocar.0016s0065	8190	1362	15018	0.000	11.03	0.09	X	Hanschen et al. 2016
VMP11	40166 (Volvox v1.0)	2750	500	5000	---	10.00	0.10		Hanschen et al. 2016
VMP3	Vocar.0016s0064	125163	19926	230400	0.000	11.56	0.09	X	Hallmann et al. 2001, Heitzer and Hallmann 2002, Hallmann 2003, Hanschen et al. 2016
VMP10	Vocar.0016s0063	19294	4443	34144	0.000	7.68	0.13	X	Hanschen et al. 2016
[without name]	Vocar.0016s0032	25	18	31	0.380	1.72	0.58		Hanschen et al. 2016
VMP37	Vocar.0007s0367	3939	1146	6731	0.000	5.87	0.17	X	Hanschen et al. 2016
VMP1 = S9	Vocar.0007s0366	80292	9410	151173	0.000	16.07	0.06	X	Hanschen et al. 2016, Shimizu et al. 2002, Tam and Kirk 1991a, Tam and Kirk 1991b; Tam et al. 1991
[without name]	Vocar.0007s0365	5547	823	10270	0.000	12.48	0.08	X	Hanschen et al. 2016
VMP40	Vocar.0029s0155	15	9	20	0.240	2.22	0.45		Hanschen et al. 2016
[without name]	Vocar.0019s0056	175	84	266	0.000	3.17	0.32	X	Hanschen et al. 2016
VMP20	42295 (Volvox v1.0)	325	50	600	---	12.00	0.08		Hanschen et al. 2016
VMP21	Vocar.0054s0052	827	96	1558	0.000	16.23	0.06	X	Hanschen et al. 2016
VMP22	Vocar.0054s0053	8013	819	15206	0.000	18.57	0.05	X	Hanschen et al. 2016
VMP23	Vocar.0054s0055	858	120	1596	0.000	13.30	0.08	X	Hanschen et al. 2016
VMP24	Vocar.0062s0036	388	494	281	0.040	0.57	1.76		Hanschen et al. 2016
VMP25	Vocar.0062s0033	524	39	1008	0.000	25.85	0.04	X	Hanschen et al. 2016
VMP6	61971 (Volvox v1.0)	28	50	5	---	0.10	10.00		Hanschen et al. 2016
VMP39	Vocar.0003s0417	21671	3669	39673	0.000	10.81	0.09	X	Hanschen et al. 2016
VMP27	Vocar.0017s0049	9	12	5	0.440	0.42	2.40		Hanschen et al. 2016
VMP28	Vocar.0017s0047	27	20	33	0.400	1.65	0.61		Hanschen et al. 2016
VMP7	Vocar.0017s0039	40	40	39	0.870	0.98	1.03		Hanschen et al. 2016

VMP26	82178 (Volvox v1.0)	20	20	20	---	1.00	1.00			Hanschen et al. 2016
[without name]	Vocar.0014s0045	726	96	1356	0.000	14.13	0.07	X		Hanschen et al. 2016
[without name]	66400 (Volvox v1.0)	0	0	0	---	---	---			Hanschen et al. 2016
VMP33	127223 (Volvox v1.0)	30	50	10	0.000	0.20	5.00		X	Hanschen et al. 2016
VMP32	127222 (Volvox v1.0)	0	0	0	---	---	---			Hanschen et al. 2016
VMP17	127219 (Volvox v1.0)	0	0	0	---	---	---			Hanschen et al. 2016
VMP16	127218 (Volvox v1.0)	0	0	0	---	---	---			Hanschen et al. 2016
VMP14	127215 (Volvox v1.0)	0	0	0	---	---	---			Hanschen et al. 2016
VMP13	66578 (Volvox v1.0)	0	0	0	---	---	---			Hanschen et al. 2016
VMP15	127216 (Volvox v1.0)	0	0	0	---	---	---			Hanschen et al. 2016
[without name]	41285 (Volvox v1.0)	0	0	0	---	---	---			Hanschen et al. 2016
[without name]	66589 (Volvox v1.0)	0	0	0	---	---	---			Hanschen et al. 2016
[without name]	97008 (Volvox v1.0)	0	0	0	---	---	---			Hanschen et al. 2016
VMP2	Vocar.0062s0024	2613	435	4791	0.000	11.01	0.09	X		Hanschen et al. 2016, Heitzer and Hallmann 2002
[without name]	Vocar.0062s0029	1103	391	1814	0.000	4.64	0.22	X		Hanschen et al. 2016
VMP19	Vocar.0062s0031	1811	119	3502	0.000	29.43	0.03	X		Hanschen et al. 2016
VMP4	Vocar.0002s0001	1646	238	3053	0.020	12.83	0.08	X		Hanschen et al. 2016
[without name]	Vocar.0001s0355	8669	1940	15397	0.000	7.94	0.13	X		Hanschen et al. 2016
[without name]	Vocar.0002s0127	10	5	15	0.160	3.00	0.33			Hanschen et al. 2016

[without name]	Vocar.0003s0191	48	20	76	0.000	3.80	0.26	X		Hanschen et al. 2016
[without name]	Vocar.0003s0288	3817	6948	686	0.000	0.10	10.13		X	Hanschen et al. 2016
[without name]	Vocar.0003s0314	829	131	1527	0.000	11.66	0.09	X		Hanschen et al. 2016
[without name]	Vocar.0004s0009	3	2	4	0.860	2.00	0.50			Hanschen et al. 2016
[without name]	Vocar.0004s0228	663	1190	136	0.000	0.11	8.75		X	Hanschen et al. 2016
[without name]	Vocar.0004s0341	56	56	56	0.910	1.00	1.00			Hanschen et al. 2016
[without name]	Vocar.0008s0181	688	791	584	0.650	0.74	1.35			Hanschen et al. 2016
[without name]	Vocar.0008s0183	279	112	446	0.080	3.98	0.25			Hanschen et al. 2016
[without name]	Vocar.0008s0184	39	8	70	0.000	8.75	0.11	X		Hanschen et al. 2016
[without name]	Vocar.0008s0187	2741	819	4663	0.000	5.69	0.18	X		Hanschen et al. 2016

[without name]	Vocar.0008s0188	407	94	719	0.000	7.65	0.13	X		Hanschen et al. 2016
[without name]	Vocar.0011s0345	661	1213	109	0.000	0.09	11.13		X	Hanschen et al. 2016
[without name]	Vocar.0011s0346	20	23	17	0.660	0.74	1.35			Hanschen et al. 2016
[without name]	Vocar20005537m_PACid_23135245 (Volvox v2.0)	---	---	---	---	---	---			Hanschen et al. 2016
[without name]	Vocar20005440m_PACid_23135260 (Volvox v2.0)	---	---	---	---	---	---			Hanschen et al. 2016
[without name]	Vocar.0011s0347	392	734	50	0.000	0.07	14.68		X	Hanschen et al. 2016
VMP12	Vocar20005748m_PACid_23135047 (Volvox v2.0)	---	---	---	---	---	---			Hanschen et al. 2016
LSG2	Vocar.0012s0179	5314	3140	7487	0.170	2.38	0.42			Hanschen et al. 2016, Shimizu et al. 2002, Tam and Kirk 1991a, Tam and Kirk 1991b; Tam et al. 1991; Nishimura et al. 2017
[without name]	Vocar.0012s0180	1361	1346	1376	0.970	1.02	0.98			Hanschen et al. 2016
[without name]	Vocar.0012s0181	717	980	453	0.060	0.46	2.16			Hanschen et al. 2016

[without name]	Vocar.0012s0269	3882	636	7128	0.000	11.21	0.09	X		Hanschen et al. 2016
[without name]	Vocar.0012s0270	26195	5129	47261	0.000	9.21	0.11	X		Hanschen et al. 2016
VMP42	Vocar.0012s0271	23232	3128	43336	0.000	13.85	0.07	X		Hanschen et al. 2016
VMP29	Vocar.0013s0001	185	52	318	0.000	6.12	0.16	X		Hanschen et al. 2016
[without name]	Vocar.0013s0081	82	19	144	0.000	7.58	0.13	X		Hanschen et al. 2016
[without name]	Vocar.0014s0029	275	211	339	0.080	1.61	0.62			Hanschen et al. 2016
[without name]	Vocar.0014s0030	26	7	44	0.000	6.29	0.16	X		Hanschen et al. 2016
[without name]	Vocar.0014s0044	463	551	374	0.120	0.68	1.47			Hanschen et al. 2016
[without name]	Vocar.0014s0062	110	187	32	0.000	0.17	5.84		X	Hanschen et al. 2016
[without name]	Vocar.0014s0203	12	13	10	0.681	0.77	1.30			Hanschen et al. 2016

[without name]	Vocar.0014s0204	191	320	62	0.000	0.19	5.16		X	Hanschen et al. 2016
[without name]	Vocar.0015s0120	6	1	11	0.017	11.00	0.09			Hanschen et al. 2016
VMP34	Vocar.0015s0198	2784	393	5175	0.000	13.17	0.08	X		Hanschen et al. 2016
[without name]	Vocar20000410m_PACid_23131656 (Volvox v2.0)	20	20	20	---	1.00	1.00			Hanschen et al. 2016
[without name]	Vocar.0017s0175	286	77	494	0.000	6.42	0.16	X		Hanschen et al. 2016
[without name]	Vocar.0017s0176	105	61	148	0.010	2.43	0.41	X		Hanschen et al. 2016
[without name]	Vocar.0021s0204	6119	2295	9942	0.000	4.33	0.23	X		Hanschen et al. 2016
[without name]	Vocar.0021s0205	2356	663	4048	0.000	6.11	0.16	X		Hanschen et al. 2016
[without name]	Vocar.0021s0205	2356	663	4048	0.000	6.11	0.16	X		Hanschen et al. 2016
[without name]	Vocar.0021s0206	247	315	178	0.050	0.57	1.77			Hanschen et al. 2016

[without name]	Vocar.0021s0208	4	6	2	0.280	0.33	3.00			Hanschen et al. 2016
[without name]	Vocar20003206m_PACid_23135857 (Volvox v2.0)	350	100	600	---	6.00	0.17			Hanschen et al. 2016
[without name]	Vocar.0021s0209	4367	498	8236	0.000	16.54	0.06	X		Hanschen et al. 2016
[without name]	Vocar.0028s0111	680	428	932	0.000	2.18	0.46	X		Hanschen et al. 2016
[without name]	Vocar.0028s0114	207	130	283	0.000	2.18	0.46	X		Hanschen et al. 2016
[without name]	Vocar.0028s0173	853	450	1256	0.010	2.79	0.36	X		Hanschen et al. 2016
[without name]	Vocar.0028s0174	16616	1900	31332	0.000	16.49	0.06	X		Hanschen et al. 2016
[without name]	Vocar.0031s0068	836	505	1166	0.000	2.31	0.43	X		Hanschen et al. 2016
[without name]	Vocar.0031s0069	330	203	457	0.000	2.25	0.44	X		Hanschen et al. 2016
[without name]	Vocar.0037s0139	482	904	60	0.000	0.07	15.07		X	Hanschen et al. 2016

[without name]	Vocar.0040s0065	18	14	22	0.440	1.57	0.64			Hanschen et al. 2016
[without name]	Vocar.0094s0001	770	1025	515	0.040	0.50	1.99			Hanschen et al. 2016
**Genes of proteins related to protein secretion and membrane trafficking according to Hanschen et al. 2016:**										
RabA	Vocar.0011s0210	1282	1996	568	0.000	0.28	3.51		X	Hanschen et al. 2016
YptV4/RabB	Vocar.0044s0035	5227	5039	5415	0.760	1.07	0.93			Hanschen et al. 2016
RabC1	Vocar.0033s0060	1660	1162	2157	0.010	1.86	0.54			Hanschen et al. 2016
RabC2	Vocar.0002s0587	388	438	338	0.320	0.77	1.30			Hanschen et al. 2016
YptV3/RabC3	Vocar.0096s0001	409	574	243	0.000	0.42	2.36		X	Hanschen et al. 2016
YptV1/RabD	Vocar.0001s1045	4943	4812	5073	0.860	1.05	0.95			Hanschen et al. 2016
YptV2/RabE	Vocar.0012s0034	967	1050	884	0.460	0.84	1.19			Hanschen et al. 2016
RabF	Vocar.0006s0349	5474	3845	7102	0.010	1.85	0.54			Hanschen et al. 2016
YptV5/RabG	Vocar.0015s0024	2178	3006	1350	0.000	0.45	2.23		X	Hanschen et al. 2016
RabH	Vocar.0001s0542	976	1553	398	0.000	0.26	3.90		X	Hanschen et al. 2016
Rabl2A	Vocar.0024s0066	1365	1046	1683	0.040	1.61	0.62			Hanschen et al. 2016
Rab23	Vocar.0002s0497	2760	769	4751	0.000	6.18	0.16	X		Hanschen et al. 2016
Fap156	Vocar.0001s0561	5542	886	10198	0.000	11.51	0.09	X		Hanschen et al. 2016

Rab28	Vocar.0026s0106	2262	1148	3375	0.000	2.94	0.34	X		Hanschen et al. 2016
Syp1	Vocar.0020s0009	1525	1918	1131	0.030	0.59	1.70			Hanschen et al. 2016
Syp3	Vocar.0032s0013	456	455	457	1.000	1.00	1.00			Hanschen et al. 2016
Syp4	Vocar.0016s0275	573	894	251	0.000	0.28	3.56		X	Hanschen et al. 2016
Syp5	Vocar.0021s0032	876	1108	644	0.030	0.58	1.72			Hanschen et al. 2016
Syp6	Vocar.0001s1502	235	274	196	0.230	0.72	1.40			Hanschen et al. 2016
Syp71	Vocar.0012s0125	1376	1333	1419	0.810	1.06	0.94			Hanschen et al. 2016
Syp8	Vocar.0005s0428	969	517	1421	0.000	2.75	0.36	X		Hanschen et al. 2016
Syp6L	Vocar.0001s1502	235	274	196	0.230	0.72	1.40			Hanschen et al. 2016
Syp2, part 1	Vocar.0001s1385	868	979	756	0.290	0.77	1.29			Hanschen et al. 2016
Syp2, part 2	Vocar.0001s1386	426	493	359	0.190	0.73	1.37			Hanschen et al. 2016
Syp2, part 3	Vocar.0001s1387	135	216	53	0.000	0.25	4.08		X	Hanschen et al. 2016
Syp2, part 4	Vocar.0001s1388	216	305	127	0.000	0.42	2.40		X	Hanschen et al. 2016
Vamp75	Vocar.0002s0246	548	631	465	0.220	0.74	1.36			Hanschen et al. 2016
Sec22	Vocar.0007s0359	1172	739	1605	0.000	2.17	0.46	X		Hanschen et al. 2016
Ykt6	Vocar.0030s0115	1195	994	1395	0.180	1.40	0.71			Hanschen et al. 2016
Vamp71	Vocar.0049s0026	1272	1159	1385	0.460	1.19	0.84			Hanschen et al. 2016

Vamp72	Vocar.0049s0025	1047	1436	657	0.000	0.46	2.19		X	Hanschen et al. 2016
Vamp73	Vocar.0049s0019	657	936	378	0.000	0.40	2.48		X	Hanschen et al. 2016
Bet1	Vocar.0011s0184	787	530	1043	0.010	1.97	0.51			Hanschen et al. 2016
Bet3.1; Bet3.2	Vocar.0014s0185	3573	2608	4538	0.010	1.74	0.57			Hanschen et al. 2016
Tml1	Vocar20008999m (Volvox v2.0)	450	600	300	---	0.50	2.00			Hanschen et al. 2016
Snap34	Vocar.0012s0108	57	89	25	0.000	0.28	3.56		X	Hanschen et al. 2016
Memb1	Vocar.0007s0349	391	347	434	0.450	1.25	0.80			Hanschen et al. 2016
SnapG1	Vocar.0008s0095	114	132	96	0.290	0.73	1.38			Hanschen et al. 2016
SnapA1	Vocar.0036s0094	3791	2576	5006	0.010	1.94	0.51			Hanschen et al. 2016
Cdc48	Vocar.0002s0167	9587	6307	12866	0.000	2.04	0.49	X		Hanschen et al. 2016
Vps45	Vocar.0006s0003	711	922	500	0.030	0.54	1.84			Hanschen et al. 2016
Vps33	Vocar.0006s0324	883	1035	731	0.230	0.71	1.42			Hanschen et al. 2016
**Genes of proteins related to actin cytoskeleton according to Hanschen et al. 2016:**										
MyoA	Vocar.0004s0052	1548	2243	853	0.000	0.38	2.63		X	Hanschen et al. 2016
MyoB	Vocar.0028s0060	209	348	69	0.000	0.20	5.04		X	Hanschen et al. 2016
MyoC	Vocar.0011s0270	883	1067	699	0.090	0.66	1.53			Hanschen et al. 2016
MyoD	Vocar.0016s0096	1751	292	3210	0.000	10.99	0.09	X		Hanschen et al. 2016
MyoE	Vocar.0011s0189	3691	3817	3564	0.810	0.93	1.07			Hanschen et al. 2016

MyoF	Vocar.0004s0053	297	328	265	0.420	0.81	1.24							Hanschen et al. 2016
ActA	Vocar.0043s0003	40985	37466	44504	0.590	1.19	0.84							Hanschen et al. 2016, Kianianmomeni and Hallmann 2013
Nap1	Vocar.0015s0188	530	68	991	0.010	14.57	0.07	X						Hanschen et al. 2016
Arp2	Vocar.0023s0174	276	202	349	0.050	1.73	0.58							Hanschen et al. 2016
Arp3	Vocar.0018s0072	627	1072	182	0.000	0.17	5.89		X					Hanschen et al. 2016
Arp4	Vocar.0038s0013	540	784	296	0.000	0.38	2.65		X					Hanschen et al. 2016
Arp6	Vocar.0042s0049	276	401	151	0.000	0.38	2.66		X					Hanschen et al. 2016
Arp7	Vocar.0007s0175	188	353	22	0.000	0.06	16.05		X					Hanschen et al. 2016
ArpC1	Vocar.0001s0562	186	203	169	0.680	0.83	1.20							Hanschen et al. 2016
ArpC2	Vocar.0039s0033	195	234	155	0.230	0.66	1.51							Hanschen et al. 2016
ArpC3	Vocar.0008s0238	307	325	288	0.870	0.89	1.13							Hanschen et al. 2016
ArpC4	Vocar.0002s0457	622	982	261	0.000	0.27	3.76		X					Hanschen et al. 2016
ForA	Vocar.0076s0007	129	203	54	0.070	0.27	3.76							Hanschen et al. 2016
AdfA	Vocar.0023s0221	576	968	184	0.000	0.19	5.26		X					Hanschen et al. 2016
gelsolin	Vocar.0001s1011	199	222	175	0.710	0.79	1.27							Hanschen et al. 2016
PrfA	Vocar.0005s0310	5158	8560	1756	0.000	0.21	4.87		X	0.19	5.30		X	Hanschen et al. 2016, Nematollahi et al. 2006

CapA	Vocar.0020s0166	386	644	127	0.030	0.20	5.07		X	Hanschen et al. 2016
VilA	Vocar.0050s0063	1537	423	2650	0.000	6.26	0.16	X		Hanschen et al. 2016
**Genes of proteins related to microtubule cytoskeleton according to Hanschen et al. 2016:**										
tubA1	Vocar.0035s0137	175307	50327	300287	0.000	5.97	0.17	X		Mages et al. 1988, Mages et al. 1995, Kianianmomeni and Hallmann 2013, Hanschen et al. 2016
TubA2	Vocar.0013s0278	235010	65584	404435	0.000	6.17	0.16	X		Hanschen et al. 2016
tubB1	Vocar.0007s0110	135505	34555	236455	0.000	6.84	0.15	X		Harper and Mages 1988, Mages et al. 1995, Kianianmomeni and Hallmann 2013, Hanschen et al. 2016
TubB2	Vocar.0007s0229	161593	36234	286952	0.000	7.92	0.13	X		Hanschen et al. 2016
TubG	Vocar.0001s0264	720	1112	327	0.000	0.29	3.40		X	Hanschen et al. 2016
TubD	Vocar.0011s0165	97	159	34	0.000	0.21	4.68		X	Hanschen et al. 2016
TubH	Vocar.0006s0287	147	173	120	0.220	0.69	1.44			Hanschen et al. 2016
TubE	Vocar.0015s0161	222	346	97	0.000	0.28	3.57		X	Hanschen et al. 2016
centrin	Vocar.0012s0203	4596	2568	6623	0.000	2.58	0.39	X		Hanschen et al. 2016
CnrA	Vocar.0050s0015	3856	884	6827	0.000	7.72	0.13	X		Hanschen et al. 2016
KatA	Vocar.0005s0308	5317	1679	8955	0.000	5.33	0.19	X		Hanschen et al. 2016
KatB	Vocar.0055s0016	1506	554	2457	0.000	4.44	0.23	X		Hanschen et al. 2016
KatC	Vocar.0005s0286	1474	1189	1759	0.100	1.48	0.68			Hanschen et al. 2016
Map1	Vocar.0039s0038	506	230	782	0.000	3.40	0.29	X		Hanschen et al. 2016
MorA	Vocar.0014s0138	1557	1566	1547	0.890	0.99	1.01			Hanschen et al. 2016

map65	Vocar.0057s0007	1671	1606	1735	0.870	1.08	0.93			Hanschen et al. 2016
EB1	Vocar.0040s0009	2114	1782	2445	0.180	1.37	0.73			Hanschen et al. 2016
Clasp	Vocar.0019s0219	4719	2791	6646	0.000	2.38	0.42	X		Hanschen et al. 2016
Spr1	Vocar.0009s0308	6161	8989	3333	0.000	0.37	2.70		X	Hanschen et al. 2016
Gcp2	Vocar.0037s0023	605	810	399	0.000	0.49	2.03		X	Hanschen et al. 2016
Gcp3	Vocar.0027s0135	430	640	219	0.000	0.34	2.92		X	Hanschen et al. 2016
Gcp4	Vocar.0045s0049	395	648	141	0.000	0.22	4.60		X	Hanschen et al. 2016
PldA	Vocar.0064s0024	988	860	1115	0.280	1.30	0.77			Hanschen et al. 2016
D1bLIC	Vocar.0032s0018	5408	1217	9599	0.000	7.89	0.13	X		Hanschen et al. 2016
DHC1b	Vocar.0038s0044	22819	5458	40180	0.000	7.36	0.14	X		Hanschen et al. 2016
Asp	Vocar.0002s0092	160	280	40	0.000	0.14	7.00		X	Hanschen et al. 2016
TtlA	Vocar.0001s0262	2605	574	4636	0.000	8.08	0.12	X		Hanschen et al. 2016
TtlB	Vocar.0004s0317	1047	191	1903	0.000	9.96	0.10	X		Hanschen et al. 2016
TtlC	Vocar.0014s0182	2125	261	3988	0.000	15.28	0.07	X		Hanschen et al. 2016
TtlD	Vocar.0032s0151	392	101	682	0.000	6.75	0.15	X		Hanschen et al. 2016
TtlE	Vocar.0007s0203	1325	1572	1078	0.130	0.69	1.46			Hanschen et al. 2016
TtlF	Vocar.0001s0608	372	108	636	0.000	5.89	0.17	X		Hanschen et al. 2016
TtlG	Vocar.0064s0020	85	130	39	0.000	0.30	3.33		X	Hanschen et al. 2016

TtlH	Vocar.0061s0020	565	187	942	0.000	5.04	0.20	X					Hanschen et al. 2016
**Genes of basal body proteins according to Hanschen et al. 2016:**													
bbs5	Vocar.0002s0180	16431	13902	18959	0.170	1.36	0.73						Hanschen et al. 2016
SFA	Vocar.0009s0093	307	363	250	0.180	0.69	1.45						Hanschen et al. 2016
bbs8	Vocar.0066s0006	4113	753	7472	0.000	9.92	0.10	X					Hanschen et al. 2016
Bbs4	Vocar.0007s0218	3133	298	5968	0.000	20.03	0.05	X					Hanschen et al. 2016
arf8	Vocar.0041s0017	746	327	1165	0.000	3.56	0.28	X					Hanschen et al. 2016
Bbs2	Vocar.0002s0661	900	307	1492	0.000	4.86	0.21	X					Hanschen et al. 2016
Bbs1	Vocar.0003s0317	5931	1767	10094	0.000	5.71	0.18	X					Hanschen et al. 2016
Ofd1	Vocar.0004s0175	1207	793	1620	0.010	2.04	0.49	X					Hanschen et al. 2016
Vfl3	Vocar.0002s0080	994	291	1696	0.000	5.83	0.17	X					Hanschen et al. 2016
bldJ	Vocar.0016s0003	90	129	51	0.050	0.40	2.53		X				Hanschen et al. 2016
bbs9	Vocar.0025s0100	1724	640	2807	0.000	4.39	0.23	X					Hanschen et al. 2016
**Genes of kinesin motor proteins according to Hanschen et al. 2016:**													
[without name]	Vocar.0018s0229	127	244	10	0.110	0.04	24.40						Hanschen et al. 2016
flaJ	Vocar.0009s0040	12066	2093	22038	0.000	10.53	0.09	X					Hanschen et al. 2016
flaH/klpA	Vocar.0006s0438	12490	6036	18943	0.000	3.14	0.32	X		21.40	0.05	X	Hanschen et al. 2016, Nematollahi et al. 2006
[without name]	Vocar.0011s0337	401	581	220	0.010	0.38	2.64		X				Hanschen et al. 2016
[without name]	Vocar.0056s0026	3125	387	5863	0.000	15.15	0.07	X					Hanschen et al. 2016

[without name]	Vocar.0001s0710	2164	457	3871	0.000	8.47	0.12	X		Hanschen et al. 2016
[without name]	Vocar.0001s0708	1078	154	2002	0.000	13.00	0.08	X		Hanschen et al. 2016
[without name]	Vocar.0005s0215	787	230	1343	0.000	5.84	0.17	X		Hanschen et al. 2016
invA	Vocar.0016s0074	2313	2837	1788	0.080	0.63	1.59			Hanschen et al. 2016, Nishii et al. 2003
[without name]	Vocar.0001s1640	142	258	25	0.000	0.10	10.32		X	Hanschen et al. 2016
[without name]	Vocar.0008s0297	5152	4371	5933	0.220	1.36	0.74			Hanschen et al. 2016
[without name]	Vocar.0047s0017	2777	2196	3358	0.100	1.53	0.65			Hanschen et al. 2016
[without name]	Vocar.0030s0142	107	207	7	0.020	0.03	29.57		X	Hanschen et al. 2016
[without name]	Vocar.0005s0163	3101	2326	3875	0.050	1.67	0.60			Hanschen et al. 2016
[without name]	Vocar.0035s0010	3185	320	6050	0.000	18.91	0.05	X		Hanschen et al. 2016
[without name]	Vocar.0035s0011	465	760	170	0.000	0.22	4.47		X	Hanschen et al. 2016
[without name]	Vocar.0035s0012	98	169	26	0.090	0.15	6.50			Hanschen et al. 2016
[without name]	Vocar.0014s0193	2704	1765	3642	0.000	2.06	0.48	X		Hanschen et al. 2016
[without name]	Vocar.0004s0058	89	167	10	0.150	0.06	16.70			Hanschen et al. 2016
[without name]	Vocar.0004s0057	560	622	498	0.380	0.80	1.25			Hanschen et al. 2016
[without name]	Vocar.0002s0592	4428	3373	5483	0.030	1.63	0.62			Hanschen et al. 2016
[without name]	Vocar.0037s0098	2664	756	4571	0.000	6.05	0.17	X		Hanschen et al. 2016
[without name]	Vocar.0003s0405	687	1346	27	0.000	0.02	49.85		X	Hanschen et al. 2016
[without name]	Vocar.0003s0462	602	780	424	0.010	0.54	1.84			Hanschen et al. 2016
[without name]	Vocar.0005s0329	4248	421	8075	0.000	19.18	0.05	X		Hanschen et al. 2016
[without name]	Vocar.0007s0156	3834	303	7364	0.000	24.30	0.04	X		Hanschen et al. 2016

[without name]	Vocar.0006s0081	3076	3112	3039	0.920	0.98	1.02			Hanschen et al. 2016
[without name]	Vocar.0040s0077	723	208	1237	0.000	5.95	0.17	X		Hanschen et al. 2016
[without name]	Vocar.0070s0026	841	184	1498	0.000	8.14	0.12	X		Hanschen et al. 2016
[without name]	Vocar.0001s0029	2035	210	3860	0.000	18.38	0.05	X		Hanschen et al. 2016
[without name]	Vocar.0017s0144	748	151	1344	0.000	8.90	0.11	X		Hanschen et al. 2016
[without name]	Vocar.0012s0070	736	211	1261	0.000	5.98	0.17	X		Hanschen et al. 2016
[without name]	Vocar.0003s0204	169	216	122	0.380	0.56	1.77			Hanschen et al. 2016
[without name]	Vocar.0029s0025	5397	835	9958	0.000	11.93	0.08	X		Hanschen et al. 2016
**Genes of cell cycle proteins according to Hanschen et al. 2016:**										
rdp1	Vocar.0082s0003	404	197	611	0.000	3.10	0.32	X		Hanschen et al. 2016
rdp2	Vocar.0007s0372	99	161	37	0.000	0.23	4.35		X	Hanschen et al. 2016
rdp3	Vocar.0005s0090	84	82	85	0.900	1.04	0.96			Hanschen et al. 2016
cdka1	Vocar.0029s0104	4574	3337	5811	0.020	1.74	0.57			Hanschen et al. 2016
cdkb1	Vocar.0065s0009	520	968	71	0.000	0.07	13.63		X	Hanschen et al. 2016
cdkc1	Vocar.0008s0098	1272	1129	1414	0.340	1.25	0.80			Hanschen et al. 2016
cdkd1	Vocar.0033s0175	950	1159	741	0.110	0.64	1.56			Hanschen et al. 2016
cdke1	Vocar.0025s0146	477	541	413	0.330	0.76	1.31			Hanschen et al. 2016

cdkg1	Vocar.0002s0394	2394	2280	2507	0.850	1.10	0.91			Hanschen et al. 2016
cdkg2	Vocar.0030s0048	35	11	59	0.010	5.36	0.19	X		Hanschen et al. 2016
cdkh1	Vocar.0076s0004	682	643	721	0.610	1.12	0.89			Hanschen et al. 2016
cdki1/lf2	Vocar.0031s0012	327	289	365	0.380	1.26	0.79			Hanschen et al. 2016
cyca1	Vocar.0011s0177	142	279	4	0.140	0.01	69.75			Hanschen et al. 2016
cycb1	Vocar.0037s0088	1242	1960	524	0.010	0.27	3.74		X	Hanschen et al. 2016
cycab1	Vocar.0029s0111	397	779	15	0.000	0.02	51.93		X	Hanschen et al. 2016
cycc1	Vocar.0068s0004	1102	1046	1158	0.650	1.11	0.90			Hanschen et al. 2016
cycd1.1	Vocar.0040s0003	587	841	333	0.000	0.40	2.53		X	Hanschen et al. 2016, Prochnik et al. 2010
cycd1.2	Vocar.0040s0002	5	7	3	0.350	0.43	2.33			Hanschen et al. 2016, Prochnik et al. 2010
cycd1.3	Vocar.0040s0001	640	1084	195	0.000	0.18	5.56		X	Hanschen et al. 2016, Prochnik et al. 2010
cycd1.4	Vocar.0030s0189	13762	4709	22814	0.000	4.84	0.21	X		Hanschen et al. 2016, Prochnik et al. 2010
cycd2	Vocar.0063s0006	7729	8671	6787	0.290	0.78	1.28			Hanschen et al. 2016, Prochnik et al. 2010
cycd3	Vocar.0013s0004	5355	5392	5317	0.780	0.99	1.01			Hanschen et al. 2016, Prochnik et al. 2010
cycd4	Vocar.0013s0190	1035	368	1701	0.000	4.62	0.22	X		Hanschen et al. 2016, Prochnik et al. 2010
cycl1	Vocar.0021s0171	1664	1074	2254	0.000	2.10	0.48	X		Hanschen et al. 2016
cycm1	Vocar.0004s0393	5607	3052	8161	0.000	2.67	0.37	X		Hanschen et al. 2016
cycu1	Vocar.0009s0009	5687	3017	8357	0.000	2.77	0.36	X		Hanschen et al. 2016

cyct1	Vocar.0057s0006	1187	1351	1023	0.310	0.76	1.32						Hanschen et al. 2016
wee1	Vocar.0076s0002	547	1068	26	0.000	0.02	41.08		X				Hanschen et al. 2016
cks1	Vocar.0015s0308	3027	1036	5017	0.000	4.84	0.21	X					Hanschen et al. 2016
RBR1 (mat3)	Vocar.0038s0042	5656	3248	8064	0.000	2.48	0.40	X		9.00	0.11	X	Nematollahi et al. 2006, Kianianmomeni et al. 2008, van den Heuvel and Dyson 2008, Hallmann 2009, Ferris et al. 2010, Hiraide et al. 2013, Hanschen et al. 2016, Olson and Nedelcu 2016
e2f1	Vocar.0001s0396	633	890	376	0.000	0.42	2.37		X				Hanschen et al. 2016, van den Heuvel and Dyson 2008
dp1	Vocar.0003s0219	1265	1236	1293	0.840	1.05	0.96						Hanschen et al. 2016, Ferris et al. 2010, van den Heuvel and Dyson 2008
e2fr1	Vocar.0001s0192	1002	1961	42	0.000	0.02	46.69		X				Hanschen et al. 2016
**Genes of cell wall proteins according to Hanschen et al. 2016:**													
gp2	Vocar.0002s0641	12205	2843	21567	0.000	7.59	0.13	X					Hanschen et al. 2016
gp3	Vocar.0030s0169	19834	4561	35106	0.000	7.70	0.13	X					Hanschen et al. 2016
**Influencial genes according to Matt and Umen 2016:**													
Hsp70A	Vocar.0006s0028	12726	21096	4355	0.000	0.21	4.84		X				Cheng et al. 2005, Cheng et al. 2006, Matt and Umen 2016
glsA	Vocar.0024s0133	440	853	27	0.000	0.03	31.59		X				Kirk 1997, Miller and Kirk 1999, Kirk 2001, Cheng et al. 2003, Cheng et al. 2005, Pappas and Miller 2009, Olson and Nedelcu 2016
invA	Vocar.0016s0074	2313	2837	1788	0.080	0.63	1.59						Matt and Umen 2016, Nishii et al. 2003
invB	Vocar.0031s0075	1316	1210	1422	0.680	1.18	0.85						Matt and Umen 2016
invC	Vocar.0002s0555	1213	1155	1270	0.730	1.10	0.91						Matt and Umen 2016
RBR1 (mat3)	Vocar.0038s0042	5656	3248	8064	0.000	2.48	0.40	X		9.00	0.11	X	Nematollahi et al. 2006, Kianianmomeni et al. 2008, van den Heuvel and Dyson 2008, Hallmann 2009, Ferris et al. 2010, Hiraide et al. 2013, Hanschen et al. 2016, Olson and Nedelcu 2016
regA	Vocar.0021s0042	2575	434	4716	0.000	10.87	0.09	X		149.70	0.01	X	Harper et al. 1987, Kirk et al. 1987, Kirk et al. 1999, Meissner et al. 1999, Stark et al. 2001, Babinger et al. 2006, Nematollahi et al. 2006, Duncan et al. 2006, Duncan et al. 2007, Olson and Nedelcu 2016, Matt and Umen 2016

**Potential target genes of regA according to Meissner et al. 1999:**														
Ycf6-like / G37	Vocar.0032s0139	69831	85444	54218	0.390	0.63	1.58							Meissner et al. 1999, Tam and Kirk 1991a, Tam and Kirk 1991b, Tam et al. 1991
LhcA / G12	Vocar.0011s0017	89488	118456	60519	0.100	0.51	1.96							Meissner et al. 1999, Tam and Kirk 1991a, Tam and Kirk 1991b, Tam et al. 1991
LhcA / G18	Vocar.0002s0333	123085	188415	57754	0.030	0.31	3.26		X					Meissner et al. 1999, Tam and Kirk 1991a, Tam and Kirk 1991b, Tam et al. 1991
LhcA / G8	Vocar.0028s0157	124868	155802	93933	0.240	0.60	1.66							Meissner et al. 1999, Tam and Kirk 1991a, Tam and Kirk 1991b, Tam et al. 1991
Li818 (G36)	Vocar.0020s0067	20392	39575	1209	0.000	0.03	32.73		X					Tam and Kirk 1991a, Tam and Kirk 1991b, Tam et al. 1991, Savard et al. 1996, Meissner et al. 1999
PsaN / G46	Vocar.0004s0500	59913	85189	34637	0.040	0.41	2.46		X					Meissner et al. 1999, Tam and Kirk 1991a, Tam and Kirk 1991b, Tam et al. 1991
PsbO / G14	Vocar.0009s0198	217896	267915	167876	0.220	0.63	1.60							Meissner et al. 1999, Tam and Kirk 1991a, Tam and Kirk 1991b, Tam et al. 1991
PsbQ / G5	Vocar.0065s0007	116831	155822	77839	0.100	0.50	2.00							Meissner et al. 1999, Tam and Kirk 1991a, Tam and Kirk 1991b, Tam et al. 1991
PetC / G9	Vocar.0009s0349	63152	77859	48444	0.340	0.62	1.61							Meissner et al. 1999, Tam and Kirk 1991a, Tam and Kirk 1991b, Tam et al. 1991
PetH / G40	Vocar.0027s0034	94359	116940	71777	0.320	0.61	1.63							Meissner et al. 1999, Tam and Kirk 1991a, Tam and Kirk 1991b, Tam et al. 1991
AtpG / G34	Vocar.0048s0019	60721	68644	52797	0.570	0.77	1.30							Meissner et al. 1999, Tam and Kirk 1991a, Tam and Kirk 1991b, Tam et al. 1991
AtpD / G45	Vocar.0009s0371	76409	85647	67171	0.610	0.78	1.28							Meissner et al. 1999, Tam and Kirk 1991a, Tam and Kirk 1991b, Tam et al. 1991
Pgk / G23	Vocar.0030s0188	118575	194810	42339	0.030	0.22	4.60		X					Meissner et al. 1999, Tam and Kirk 1991a, Tam and Kirk 1991b, Tam et al. 1991
Pdi / G15	Vocar.0082s0014	16804	11305	22302	0.000	1.97	0.51							Meissner et al. 1999, Tam and Kirk 1991a, Tam and Kirk 1991b, Tam et al. 1991
**Genes investigated by Tam et al. 1991:**														
gon167	Vocar.0001s1685	82	149	15	0.000	0.10	9.93		X	0.17	5.80		X	Tam and Kirk 1991a, Tam and Kirk 1991b, Tam et al. 1991, Nematollahi et al. 2006
G1	Vocar.0071s0004	6801	3264	10337	0.000	3.17	0.32	X						Tam and Kirk 1991a, Tam and Kirk 1991b, Tam et al. 1991
G5 / PsbQ	Vocar.0065s0007	116831	155822	77839	0.100	0.50	2.00							Meissner et al. 1999, Tam and Kirk 1991a, Tam and Kirk 1991b, Tam et al. 1991
G8 / LhcA	Vocar.0028s0157	124868	155802	93933	0.240	0.60	1.66							Meissner et al. 1999, Tam and Kirk 1991a, Tam and Kirk 1991b, Tam et al. 1991
G9 / PetC	Vocar.0009s0349	63152	77859	48444	0.340	0.62	1.61							Meissner et al. 1999, Tam and Kirk 1991a, Tam and Kirk 1991b, Tam et al. 1991
G12 / LhcA	Vocar.0011s0017	89488	118456	60519	0.100	0.51	1.96							Meissner et al. 1999, Tam and Kirk 1991a, Tam and Kirk 1991b, Tam et al. 1991
G14 / PsbO	Vocar.0009s0198	217896	267915	167876	0.220	0.63	1.60							Meissner et al. 1999, Tam and Kirk 1991a, Tam and Kirk 1991b, Tam et al. 1991
G15 / Pdi	Vocar.0082s0014	16804	11305	22302	0.000	1.97	0.51							Meissner et al. 1999, Tam and Kirk 1991a, Tam and Kirk 1991b, Tam et al. 1991
G18 / LhcA	Vocar.0002s0333	123085	188415	57754	0.030	0.31	3.26		X					Meissner et al. 1999, Tam and Kirk 1991a, Tam and Kirk 1991b, Tam et al. 1991
G21	Vocar.0001s1168	12415	20728	4101	0.000	0.20	5.05		X					Tam and Kirk 1991a, Tam and Kirk 1991b, Tam et al. 1991

G23 / Pgk	Vocar.0030s0188	118575	194810	42339	0.030	0.22	4.60		X					Meissner et al. 1999, Tam and Kirk 1991a, Tam and Kirk 1991b, Tam et al. 1991
G30	Vocar.0030s0054	13491	24055	2927	0.000	0.12	8.22		X	0.15	6.62		X	Nematollahi et al. 2006, Tam and Kirk 1991a, Tam and Kirk 1991b, Tam et al. 1991
G32	Vocar.0073s0017	5763	10250	1275	0.000	0.12	8.04		X					Meissner et al. 1999, Tam and Kirk 1991a, Tam and Kirk 1991b, Tam et al. 1991
G34	Vocar.0048s0019	60721	68644	52797	0.570	0.77	1.30							Meissner et al. 1999, Tam and Kirk 1991a, Tam and Kirk 1991b, Tam et al. 1991
Li818 (G36)	Vocar.0020s0067	20392	39575	1209	0.000	0.03	32.73		X					Tam and Kirk 1991a, Tam and Kirk 1991b, Tam et al. 1991, Savard et al. 1996, Meissner et al. 1999
G37 / Ycf6-like	Vocar.0032s0139	69831	85444	54218	0.390	0.63	1.58							Meissner et al. 1999, Tam and Kirk 1991a, Tam and Kirk 1991b, Tam et al. 1991
G40 / PetH	Vocar.0027s0034	94359	116940	71777	0.320	0.61	1.63							Meissner et al. 1999, Tam and Kirk 1991a, Tam and Kirk 1991b, Tam et al. 1991
G45 / AtpD	Vocar.0009s0371	76409	85647	67171	0.610	0.78	1.28							Meissner et al. 1999, Tam and Kirk 1991a, Tam and Kirk 1991b, Tam et al. 1991
G46 / PsaN	Vocar.0004s0500	59913	85189	34637	0.040	0.41	2.46		X					Meissner et al. 1999, Tam and Kirk 1991a, Tam and Kirk 1991b, Tam et al. 1991
alpha tubulin	Vocar.0035s0137	175307	50327	300287	0.000	5.97	0.17	X		6.00	0.17	X		Hanschen et al. 2016, Tam and Kirk 1991a, Tam and Kirk 1991b, Tam et al. 1991, Meissner et al. 1999, Kianianmomeni and Hallmann 2013
SSG185 = ssgA	Vocar.0002s0564	50529	7584	93473	0.000	12.33	0.08	X		46,00/ 10,00		X		Ertl et al. 1989, Holst et al. 1989, Tam and Kirk 1991a, Tam and Kirk 1991b, Tam et al. 1991, Mengele and Sumper 1992, Sumper and Hallmann 1998, Hallmann 2003, Nematollahi et al. 2006
**Genes of photoreceptors investigated by Kianianmomeni and Hallmann 2015:**														
VcCRYp	Vocar.0003s0260	2508	1321	3694	0.000	2.80	0.36	X		6.00	0.17	X		Kianianmomeni and Hallmann 2015
VcCRYa	Vocar.0002s0198	35	40	30	0.440	0.75	1.33			0.60	1.67			Kianianmomeni and Hallmann 2015
VcCRYd1	Vocar.0004s0454	303	170	435	0.000	2.56	0.39	X		0.91	1.10			Kianianmomeni and Hallmann 2015
VcCRXd2	Vocar.0004s0452	306	293	318	0.680	1.09	0.92			0.56	1.79			Kianianmomeni and Hallmann 2015
VcPhot1	Vocar.0011s0213	11303	4395	18210	0.000	4.14	0.24	X		8.00	0.13	X		Kianianmomeni and Hallmann 2015
VChR1	Vocar.0050s0060	2947	186	5708	0.000	30.69	0.03	X		700.00	0.00	X		Kianianmomeni et al. 2009, Kianianmomeni and Hallmann 2015
VChR2	Vocar.0028s0176	837	82	1591	0.000	19.40	0.05	X		650.00	0.00	X		Kianianmomeni et al. 2009, Kianianmomeni and Hallmann 2015
VcHKR1	Vocar.0044s0018	2215	886	3544	0.000	4.00	0.25	X		6.00	0.17	X		Kianianmomeni and Hallmann 2015
VcHKR2	Vocar.0009s0380	1020	732	1307	0.020	1.79	0.56	(X)		4.00	0.25	X		Kianianmomeni and Hallmann 2015
VcHKR3	Vocar.0001s0831	4654	2258	7050	0.000	3.12	0.32	X		3.80	0.26	X		Kianianmomeni and Hallmann 2015
VcHKR4	Vocar.0069s0008	529	284	773	0.000	2.72	0.37	X		8.00	0.13	X		Kianianmomeni and Hallmann 2015
VR1 (vop)	Vocar.0024s0227	8946	16313	1578	0.000	0.10	10.34		X	0.13	8.00		X	Ebnet et al. 1999, Kianianmomeni and Hallmann 2015

References

Amon P, Haas E, Sumper M (1998) The sex-inducing pheromone and wounding trigger the same set of genes in the multicellular green alga *Volvox*. Plant Cell 10: 781-789

Babinger K, Hallmann A, Schmitt R (2006) Translational control of *regA*, a key gene controlling cell differentiation in *Volvox carteri*. Development 133: 4045-4051

Cheng Q, Fowler R, Tam LW, Edwards L, Miller SM (2003) The role of GlsA in the evolution of asymmetric cell division in the green alga *Volvox carteri*. Dev. Genes Evol. 213: 328-335

Cheng Q, Hallmann A, Edwards L, Miller SM (2006) Characterization of a heat-shock-inducible *hsp70* gene of the green alga *Volvox carteri*. Gene 371: 112-120

Cheng Q, Pappas V, Hallmann A, Miller SM (2005) Hsp70A and GlsA interact as partner chaperones to regulate asymmetric division in *Volvox*. Dev. Biol. 286: 537-548

Duncan L, Nishii I, Harryman A, Buckley S, Howard A, Friedman NR, Miller SM (2007) The VARL gene family and the evolutionary origins of the master cell-type regulatory gene, *regA*, in *Volvox carteri*. J. Mol. Evol. 65: 1-11

Duncan L, Nishii I, Howard A, Kirk D, Miller SM (2006) Orthologs and paralogs of *regA*, a master cell-type regulatory gene in *Volvox carteri*. Curr. Genet. 50: 61-72

Ebnet E, Fischer M, Deininger W, Hegemann P (1999) Volvoxrhodopsin, a light-regulated sensory photoreceptor of the spheroidal green alga *Volvox carteri*. Plant Cell 11: 1473-1484

Ender F, Godl K, Wenzl S, Sumper M (2002) Evidence for autocatalytic cross-linking of hydroxyproline-rich glycoproteins during extracellular matrix assembly in *Volvox*. Plant Cell 14: 1147-1160.

Ender F, Hallmann A, Amon P, Sumper M (1999) Response to the sexual pheromone and wounding in the green alga *Volvox*: induction of an extracellular glycoprotein consisting almost exclusively of hydroxyproline. J. Biol. Chem. 274: 35023-35028

Ertl H, Mengele R, Wenzl S, Engel J, Sumper M (1989) The extracellular matrix of *Volvox carteri*: molecular structure of the cellular compartment. J. Cell Biol. 109: 3493-3501

Ferris P, Olson BJ, De Hoff PL, Douglass S, Casero D, Prochnik S, Geng S, Rai R, Grimwood J, Schmutz J, Nishii I, Hamaji T, Nozaki H, Pellegrini M, Umen JG (2010) Evolution of an expanded sex-determining locus in *Volvox*. Science 328: 351-354

Fukada K, Inoue T, Shiraishi H (2006) A posttranslationally regulated protease, VheA, is involved in the liberation of juveniles from parental spheroids in *Volvox carteri*. Plant Cell 18: 2554-2566

Godl K, Hallmann A, Rappel A, Sumper M (1995) Pherophorins: a family of extracellular matrix glycoproteins from *Volvox* structurally related to the sex-inducing pheromone. Planta 196: 781-787

Godl K, Hallmann A, Wenzl S, Sumper M (1997) Differential targeting of closely related ECM glycoproteins: the pherophorin family from *Volvox*. EMBO J. 16: 25-34

Hallmann A (1999) Enzymes in the extracellular matrix of *Volvox*: an inducible, calcium-dependent phosphatase with a modular composition. J. Biol. Chem. 274: 1691-1697

Hallmann A (2003) Extracellular matrix and sex-inducing pheromone in *Volvox*. Int. Rev. Cytol. 227: 131-182

Hallmann A (2006) The pherophorins: common, versatile building blocks in the evolution of extracellular matrix architecture in Volvocales. Plant J. 45: 292-307

Hallmann A (2009) Key elements of the retinoblastoma tumor suppressor pathway in *Volvox carteri*. Commun. Integr. Biol. 2: 396-399

Hallmann A, Amon P, Godl K, Heitzer M, Sumper M (2001) Transcriptional activation by the sexual pheromone and wounding: a new gene family from *Volvox* encoding modular proteins with (hydroxy)proline-rich and metalloproteinase homology domains. Plant J. 26: 583-593

Hanschen ER, Marriage TN, Ferris PJ, Hamaji T, Toyoda A, Fujiyama A, Neme R, Noguchi H, Minakuchi Y, Suzuki M, Kawai-Toyooka H, Smith DR, Sparks H, Anderson J, Bakaric R, Luria V, Karger A, Kirschner MW, Durand PM, Michod RE, Nozaki H, Olson BJ (2016) The *Gonium pectorale* genome demonstrates co-option of cell cycle regulation during the evolution of multicellularity. Nature communications 7: 11370

Harper JF, Huson KS, Kirk DL (1987) Use of repetitive sequences to identify DNA polymorphisms linked to *regA*, a developmentally important locus in *Volvox*. Genes Dev. 1: 573-584

Harper JF, Mages W (1988) Organization and structure of *Volvox* beta-tubulin genes. Mol. Gen. Genet. 213: 315-324

Heitzer M, Hallmann A (2002) An extracellular matrix-localized metalloproteinase with an exceptional QEXXH metal binding site prefers copper for catalytic activity. J. Biol. Chem. 277: 28280-28286.

Hiraide R, Kawai-Toyooka H, Hamaji T, Matsuzaki R, Kawafune K, Abe J, Sekimoto H, Umen J, Nozaki H (2013) The evolution of male-female sexual dimorphism predates the gender-based divergence of the mating locus gene *MAT3*/*RB*. Mol. Biol. Evol. 30: 1038-1040

Holst O, Christoffel V, Fründ R, Moll H, Sumper M (1989) A phosphodiester bridge between two arabinose residues as a structural element of an extracellular glycoprotein of *Volvox carteri*. Eur. J. Biochem. 181: 345-350

Huber O, Sumper M (1994) Algal-CAMs: isoforms of a cell adhesion molecule in embryos of the alga *Volvox* with homology to *Drosophila* fasciclin I. EMBO J. 13: 4212-4222

Kianianmomeni A, Hallmann A (2013) Validation of reference genes for quantitative gene expression studies in *Volvox carteri* using real-time RT-PCR. Mol. Biol. Rep. 40: 6691-6699

Kianianmomeni A, Hallmann A (2015) Transcriptional analysis of *Volvox* photoreceptors suggests the existence of different cell-type specific light-signaling pathways. Curr. Genet. 61: 3-18

Kianianmomeni A, Nematollahi G, Hallmann A (2008) A gender-specific retinoblastoma-related protein in *Volvox carteri* implies a role for the retinoblastoma protein family in sexual development. Plant Cell 20: 2399-2419

Kianianmomeni A, Stehfest K, Nematollahi G, Hegemann P, Hallmann A (2009) Channelrhodopsins of *Volvox carteri* are photochromic proteins that are specifically expressed in somatic cells under control of light, temperature, and the sex inducer. Plant Physiol. 151: 347-366

Kirk DL (1997) The genetic program for germ-soma differentiation in *Volvox*. Annu. Rev. Genet. 31: 359-380

Kirk DL (2001) Germ-soma differentiation in *Volvox*. Dev. Biol. 238: 213-223

Kirk DL, Baran GJ, Harper JF, Huskey RJ, Huson KS, Zagris N (1987) Stage-specific hypermutability of the *regA* locus of *Volvox*, a gene regulating the germ-soma dichotomy. Cell 48: 11-24

Kirk MM, Stark K, Miller SM, Müller W, Taillon BE, Gruber H, Schmitt R, Kirk DL (1999) *regA*, a *Volvox* gene that plays a central role in germ-soma differentiation, encodes a novel regulatory protein. Development 126: 639-647

Mages W, Cresnar B, Harper JF, Brüderlein M, Schmitt R (1995) *Volvox carteri* alpha 2- and beta 2-tubulin-encoding genes: regulatory signals and transcription. Gene 160: 47-54

Mages W, Salbaum JM, Harper JF, Schmitt R (1988) Organization and structure of *Volvox* alpha-tubulin genes. Mol. Gen. Genet. 213: 449-458

Matt G, Umen J (2016) *Volvox*: A simple algal model for embryogenesis, morphogenesis and cellular differentiation. Dev. Biol. 419: 99-113

Meissner M, Stark K, Cresnar B, Kirk DL, Schmitt R (1999) *Volvox* germline-specific genes that are putative targets of RegA repression encode chloroplast proteins. Curr. Genet. 36: 363-370

Mengele R, Sumper M (1992) Gulose as a constituent of a glycoprotein. FEBS Lett. 298: 14-16

Miller SM, Kirk DL (1999) *glsA*, a *Volvox* gene required for asymmetric division and germ cell specification, encodes a chaperone-like protein. Development 126: 649-658

Mitchell DR, Brown KS (1994) Sequence analysis of the *Chlamydomonas* alpha and beta dynein heavy chain genes. J. Cell Sci. 107 (Pt 3): 635-644

Mitchell DR, Brown KS (1997) Sequence analysis of the *Chlamydomonas reinhardtii* flagellar alpha dynein gene. Cell Motil Cytoskeleton 37: 120-126

Myster SH, Knott JA, O'Toole E, Porter ME (1997) The *Chlamydomonas Dhc1* gene encodes a dynein heavy chain subunit required for assembly of the I1 inner arm complex. Mol. Biol. Cell 8: 607-620

Nematollahi G, Kianianmomeni A, Hallmann A (2006) Quantitative analysis of cell-type specific gene expression in the green alga *Volvox carteri*. BMC Genomics 7: 321

Nishii I, Ogihara S, Kirk DL (2003) A kinesin, invA, plays an essential role in *Volvox* morphogenesis. Cell 113: 743-753

Nishimura M, Nagashio R, Sato Y, Hasegawa T (2017) Late Somatic Gene 2 disrupts parental spheroids cooperatively with *Volvox* hatching enzyme A in *Volvox*. Planta 245: 183-192

Olson BJ, Nedelcu AM (2016) Co-option during the evolution of multicellular and developmental complexity in the volvocine green algae. Curr. Opin. Genet. Dev. 39: 107-115

Pappas V, Miller SM (2009) Functional analysis of the *Volvox carteri* asymmetric division protein GlsA. Mech. Dev. 126: 842-851

Porter ME, Knott JA, Myster SH, Farlow SJ (1996) The dynein gene family in *Chlamydomonas reinhardtii*. Genetics 144: 569-585

Prochnik SE, Umen J, Nedelcu AM, Hallmann A, Miller SM, Nishii I, Ferris P, Kuo A, Mitros T, Fritz-Laylin LK, Hellsten U, Chapman J, Simakov O, Rensing SA, Terry A, Pangilinan J, Kapitonov V, Jurka J, Salamov A, Shapiro H, Schmutz J, Grimwood J, Lindquist E, Lucas S, Grigoriev IV, Schmitt R, Kirk D, Rokhsar DS (2010) Genomic analysis of organismal complexity in the multicellular green alga *Volvox carteri*. Science 329: 223-226

Savard F, Richard C, Guertin M (1996) The *Chlamydomonas reinhardtii LI818* gene represents a distant relative of the *cabI/II* genes that is regulated during the cell cycle and in response to illumination. Plant Mol. Biol. 32: 461-473

Shimizu T, Inoue T, Shiraishi H (2002) Cloning and characterization of novel extensin-like cDNAs that are expressed during late somatic cell phase in the green alga *Volvox carteri*. Gene 284: 179-187

Stark K, Kirk DL, Schmitt R (2001) Two enhancers and one silencer located in the introns of regA control somatic cell differentiation in *Volvox carteri*. Genes Dev. 15: 1449-1460

Sumper M, Berg E, Wenzl S, Godl K (1993) How a sex pheromone might act at a concentration below 10^{-16} M. EMBO J. 12: 831-836

Sumper M, Hallmann A (1998) Biochemistry of the extracellular matrix of *Volvox*. Int. Rev. Cytol. 180: 51-85

Tam LW, Kirk DL (1991a) Identification of cell-type-specific genes of *Volvox carteri* and characterization of their expression during the asexual life cycle. Dev. Biol. 145: 51-66

Tam LW, Kirk DL (1991b) The program for cellular differentiation in *Volvox carteri* as revealed by molecular analysis of development in a gonidialess/somatic regenerator mutant. Development 112: 571-580

Tam LW, Stamer KA, Kirk DL (1991) Early and late gene expression programs in developing somatic cells of *Volvox carteri*. Dev. Biol. 145: 67-76

van den Heuvel S, Dyson NJ (2008) Conserved functions of the pRB and E2F families. Nat. Rev. Mol. Cell Biol. 9: 713-724

Yagi T, Minoura I, Fujiwara A, Saito R, Yasunaga T, Hirono M, Kamiya R (2005) An axonemal dynein particularly important for flagellar movement at high viscosity. Implications from a new *Chlamydomonas* mutant deficient in the dynein heavy chain gene *DHC9*. J. Biol. Chem. 280: 41412-41420
