# Supplementary material for: Whole transcriptome RNA-Seq analysis reveals extensive cell type-specific compartmentalization in Volvox carteri
Source: BMC Biol. 2017 Nov 28;15:111. doi: 10.1186/s12915-017-0450-y (PMC5704591; doi:10.1186/s12915-017-0450-y)
Supplement: Supplementary file 5 — Functional enrichment analysis of the most highly expressed genes in somatic cells, reproductive cells, and in total. (PDF 42 kb) [file 12915_2017_450_MOESM5_ESM.pdf]

Additional file 5: Table S4. Functional enrichment analysis of the most highly expressed genes in somatic cells, reproductive cells, and in total.

X = gene present in intersection of top 50 somatic cells, top 50 reproductive cells and top 50 in total
X = gene present in intersection of top 50 somatic cells and top 50 reproductive cells
X = gene present in intersection of top 50 somatic cells and top 50 in total
X = gene present in intersection of top 50 reproductive cells and top 50 in total

top 50 - somatic cells

rank in expression level				locusName	defline	baseMean somatic cells	functional group
1			X	Vocar.0013s0278	tubulin alpha-2 chain	404435	flagella associated
2			X	Vocar.0070s0007	flagellar associated protein	321906	flagella associated
3	X	X	X	Vocar.0013s0021	glyceraldehyde-3-phosphate dehydrogenase B subunit	315268	glycolysis
4			X	Vocar.0035s0137	tubulin alpha-2 chain	300287	flagella associated
5	X	X	X	Vocar.0008s0418	fructose-bisphosphate aldolase 2	299645	glycolysis
6			X	Vocar.0007s0229	tubulin beta chain 2	286952	flagella associated
7			X	Vocar.0059s0039	cysteine proteinases superfamily protein	259801	protein synthesis and degradation
8	X	X	X	Vocar.0001s0479	photosystem II light harvesting complex gene 2.1	251375	photosynthesis
9			X	Vocar.0007s0110	tubulin beta chain 2	236455	flagella associated
10			X	Vocar.0032s0153	PLAC8 family protein	235621	other functions
11	X	X	X	Vocar.0007s0216	photosystem II light harvesting complex gene 2.1	232376	photosynthesis
12			X	Vocar.0016s0064	gametolysin peptidase M11 (peptidase M11)	230400	ECM compound
13	X	X	X	Vocar.0002s0517	GTP binding elongation factor Tu family protein	215035	protein synthesis and degradation
14	X	X	X	Vocar.0007s0215	photosystem II light harvesting complex gene 2.2	204724	photosynthesis
15			X	Vocar.0007s0404	unknown function	195521	other functions
16	X	X	X	Vocar.0018s0037	light harvesting complex of photosystem II 5	173401	photosynthesis
17	X	X	X	Vocar.0009s0198	PS II oxygen-evolving complex 1	167876	photosynthesis
18				Vocar.0004s0255	extracellular matrix glycoprotein pherophorin I	158963	ECM compound
19				Vocar.0034s0104	pherophorin (DUF3707)	156488	ECM compound
20				Vocar.0007s0366	gametolysin / lysin	151173	ECM compound
21				Vocar.0001s1548	PLAC8 family protein	146314	other functions
22				Vocar.0015s0089	unknown function	142805	other functions
23			X	Vocar.0009s0275	glycine-rich RNA-binding protein 2	129056	protein synthesis and degradation
24	X	X	X	Vocar.0021s0124	light harvesting complex photosystem II	128005	photosynthesis
25				Vocar.0059s0041	small cysteine-rich extracellular protein	122224	ECM compound
26				Vocar.0003s0105	xylem serine peptidase 1	121983	protein synthesis and degradation
27	X	X	X	Vocar.0007s0217	Rho-GTPase activating protein, pleckstrin homology	117621	other functions
28	X	X	X	Vocar.0015s0272	plastocyanin 1	111593	photosynthesis
29				Vocar.0003s0418	unknown function	108902	other functions
30	X	X	X	Vocar.0002s0459	photosystem II subunit R	108192	photosynthesis
31	X	X	X	Vocar.0007s0361	phosphoribulokinase	103838	photosynthesis

32	X	X	X	Vocar.0009s0370	photosystem I light harvesting complex gene 3	100175	photosynthesis
33	X	X	X	Vocar.0002s0071	photosystem I light harvesting complex gene 1	96661	photosynthesis
34	X	X	X	Vocar.0042s0039	CP12 domain-containing protein 1	95506	photosynthesis
35	X	X	X	Vocar.0028s0157	photosystem I light harvesting complex gene 2	93933	photosynthesis
36				Vocar.0002s0564	pherophorin (DUF3707)	93473	ECM compound
37				Vocar.0001s0636	formate C-acetyltransferase / pyruvate formate-lyase	92664	other functions
38	X	X	X	Vocar.0017s0129	photosystem I subunit D-2	90263	photosynthesis
39	X	X	X	Vocar.0006s0098	photosystem I subunit F	90199	photosynthesis
40			X	Vocar.0001s1775	photosystem II light harvesting complex gene 2.3	89405	photosynthesis
41			X	Vocar.0009s0214	ribosomal protein L4/L1 family	87526	protein synthesis and degradation
42	X	X	X	Vocar.0027s0094	photosystem II reaction center W	87506	photosynthesis
43				Vocar.0036s0057	DC1 domain-containing protein	85154	other functions
44	X	X	X	Vocar.0036s0111	ribosomal protein L1p/L10e family	82963	protein synthesis and degradation
45	X	X	X	Vocar.0016s0012	ribosomal protein 1	81380	protein synthesis and degradation
46				Vocar.0066s0009	ribosomal protein S30 family protein	80374	protein synthesis and degradation
47				Vocar.0006s0331	ribosomal protein S5/elongation factor G/III/V family protein	80259	protein synthesis and degradation
48	X	X	X	Vocar.0005s0361	light-harvesting chlorophyll-protein complex I subunit A4	80162	photosynthesis
49	X	X	X	Vocar.0004s0497	photosystem II subunit X	79848	photosynthesis
50	X	X	X	Vocar.0007s0241	DNA repair protein Rev1	79116	other functions

top 50 - reproductive cells

rank in expression level					locusName	defline	baseMean reproductive cells	functional group
1	X	X		X	Vocar.0013s0021	glyceraldehyde-3-phosphate dehydrogenase B subunit	428603	glycolysis
2	X	X		X	Vocar.0008s0418	fructose-bisphosphate aldolase 2	406555	glycolysis
3	X	X		X	Vocar.0018s0037	light harvesting complex of photosystem II 5	279882	photosynthesis
4	X	X		X	Vocar.0009s0198	PS II oxygen-evolving complex 1	267915	photosynthesis
5	X	X		X	Vocar.0021s0124	light harvesting complex photosystem II	267638	photosynthesis
6	X	X		X	Vocar.0001s0479	photosystem II light harvesting complex gene 2.1	229075	photosynthesis
7	X	X		X	Vocar.0007s0216	photosystem II light harvesting complex gene 2.1	203701	photosynthesis
8	X	X		X	Vocar.0002s0517	GTP binding elongation factor Tu family protein	202576	protein synthesis and degradation
9				X	Vocar.0030s0188	phosphoglycerate kinase 1	194810	glycolysis
10	X	X		X	Vocar.0002s0071	photosystem I light harvesting complex gene 1	190236	photosynthesis
11				X	Vocar.0002s0333	photosystem I light harvesting complex gene 5	188415	photosynthesis
12	X	X		X	Vocar.0009s0370	photosystem I light harvesting complex gene 3	187414	photosynthesis
13	X	X		X	Vocar.0042s0039	CP12 domain-containing protein 1	185149	photosynthesis
14	X	X		X	Vocar.0007s0215	photosystem II light harvesting complex gene 2.2	183301	photosynthesis
15	X	X		X	Vocar.0005s0361	light-harvesting chlorophyll-protein complex I subunit A4	182454	photosynthesis
16	X	X		X	Vocar.0007s0241	DNA-directed DNA polymerase	180875	other functions
17				X	Vocar.0002s0045	unknown function	172594	other functions
18				X	Vocar.0002s0047	photosystem II light harvesting complex gene 2.3	167691	photosynthesis
19				X	Vocar.0012s0221	photosystem II light harvesting complex gene 2.2	163774	photosynthesis
20	X	X		X	Vocar.0007s0361	phosphoribulokinase	157464	photosynthesis
21				X	Vocar.0065s0007	photosystem II subunit Q-2	155822	photosynthesis
22	X	X		X	Vocar.0028s0157	photosystem I light harvesting complex gene 2	155802	photosynthesis

23	X	X		X	Vocar.0004s0497	photosystem II subunit X	154216	photosynthesis
24				X	Vocar.0023s0180	photosystem I light harvesting complex gene 5	150550	photosynthesis
25	X	X		X	Vocar.0017s0129	photosystem I subunit D-2	147954	photosynthesis
26				X	Vocar.0002s0040	Golgi to ER traffic protein 4 homolog	145409	other functions
27	X	X		X	Vocar.0006s0098	photosystem I subunit F	141547	photosynthesis
28	X	X		X	Vocar.0002s0459	photosystem II subunit R	140259	photosynthesis
29				X	Vocar.0016s0243	light-harvesting chlorophyll-protein complex I subunit A4	137348	photosynthesis
30	X	X		X	Vocar.0015s0272	plastocyanin 1	133780	photosynthesis
31				X	Vocar.0016s0158	photosystem I subunit l	128786	photosynthesis
32	X	X		X	Vocar.0027s0094	photosystem II reaction center W	122259	photosynthesis
33				X	Vocar.0011s0017	photosystem I light harvesting complex gene 5	118456	photosynthesis
34				X	Vocar.0027s0034	root FNR 2	116940	other functions
35				X	Vocar.0001s0400	oxygen evolving enhancer protein 3 (psbQ)	114257	photosynthesis
36					Vocar.0003s0404	flagellar associated protein	106706	flagella associated
37	X	X		X	Vocar.0036s0111	ribosomal protein L1p/L10e family	104309	protein synthesis and degradation
38	X	X		X	Vocar.0007s0217	Rho-GTPase activating protein, pleckstrin homology	103942	other functions
39					Vocar.0012s0172	photosystem II light harvesting complex gene 2.2	101201	photosynthesis
40					Vocar.0020s0109	nucleotide transporter 1	99165	other functions
41					Vocar.0002s0051	photosystem II light harvesting complex gene 2.3	98894	photosynthesis
42					Vocar.0002s0048	photosystem II light harvesting complex gene 2.3	98711	photosynthesis
43					Vocar.0002s0456	ATPase, F1 complex, gamma subunit protein	96548	other functions
44					Vocar.0024s0047	photosystem I subunit K	96472	photosynthesis
45					Vocar.0002s0044	photosystem II light harvesting complex gene 2.3	95033	photosynthesis
46					Vocar.0002s0055	photosystem II light harvesting complex gene 2.1	94080	photosynthesis
47	X	X		X	Vocar.0016s0012	ribosomal protein 1	93679	protein synthesis and degradation
48					Vocar.0002s0042	photosystem II light harvesting complex gene 2.3	93578	photosynthesis
49					Vocar.0001s1032	photosystem I subunit G	89794	photosynthesis
50					Vocar.0013s0247	photosystem I reaction center subunit O	89582	photosynthesis

top 50 - in total

rank in expression level				locusName	define	baseMean	functional group
1	X	X	X	Vocar.0013s0021	glyceraldehyde-3-phosphate dehydrogenase B subunit	371936	glycolysis
2	X	X	X	Vocar.0008s0418	fructose-bisphosphate aldolase 2	353100	glycolysis
3	X	X	X	Vocar.0001s0479	photosystem II light harvesting complex gene 2.1	240225	photosynthesis
4		X		Vocar.0013s0278	tubulin alpha-2 chain	235010	flagella associated
5	X	X	X	Vocar.0018s0037	light harvesting complex of photosystem II 5	226642	photosynthesis
6	X	X	X	Vocar.0007s0216	photosystem II light harvesting complex gene 2.1	218039	photosynthesis
7	X	X	X	Vocar.0009s0198	PS II oxygen-evolving complex 1	217895	photosynthesis
8	X	X	X	Vocar.0002s0517	GTP binding elongation factor Tu family protein	208806	protein synthesis and degradation
9	X	X	X	Vocar.0021s0124	light harvesting complex photosystem II	197822	photosynthesis
10	X	X	X	Vocar.0007s0215	photosystem II light harvesting complex gene 2.2	194012	photosynthesis
11		X		Vocar.0035s0137	tubulin alpha-2 chain	175307	flagella associated
12		X		Vocar.0070s0007	flagellar associated protein	168528	flagella associated
13		X		Vocar.0007s0229	tubulin beta chain 2	161593	flagella associated

14	X	X	X	Vocar.0009s0370	photosystem I light harvesting complex gene 3	143794	photosynthesis
15	X	X	X	Vocar.0002s0071	photosystem I light harvesting complex gene 1	143448	photosynthesis
16	X	X	X	Vocar.0042s0039	CP12 domain-containing protein 1	140327	photosynthesis
17		X		Vocar.0007s0110	tubulin beta chain 2	135505	flagella associated
18		X		Vocar.0059s0039	cysteine proteinases superfamily protein	135132	protein synthesis and degradation
19	X	X	X	Vocar.0005s0361	light-harvesting chlorophyll-protein complex I subunit A4	131308	photosynthesis
20	X	X	X	Vocar.0007s0361	phosphoribulokinase	130651	photosynthesis
21	X	X	X	Vocar.0007s0241	DNA-directed DNA polymerase	129996	other functions
22		X		Vocar.0016s0064	gametolysin peptidase M11 (peptidase M11)	125163	ECM compound
23	X	X	X	Vocar.0028s0157	photosystem I light harvesting complex gene 2	124867	photosynthesis
24	X	X	X	Vocar.0002s0459	photosystem II subunit R	124226	photosynthesis
25		X		Vocar.0032s0153	PLAC8 family protein	124146	other functions
26			X	Vocar.0002s0333	photosystem I light harvesting complex gene 5	123085	photosynthesis
27	X	X	X	Vocar.0015s0272	plastocyanin 1	122686	photosynthesis
28	X	X	X	Vocar.0017s0129	photosystem I subunit D-2	119108	photosynthesis
29			X	Vocar.0030s0188	phosphoglycerate kinase 1	118575	glycolysis
30	X	X	X	Vocar.0004s0497	photosystem II subunit X	117032	photosynthesis
31			X	Vocar.0065s0007	photosystem II subunit Q-2	116830	photosynthesis
32	X	X	X	Vocar.0006s0098	photosystem I subunit F	115873	photosynthesis
33			X	Vocar.0023s0180	photosystem I light harvesting complex gene 5	114082	photosynthesis
34			X	Vocar.0002s0045	unknown function	112688	other functions
35	X	X	X	Vocar.0007s0217	Rho-GTPase activating protein, pleckstrin homology	110781	other functions
36			X	Vocar.0002s0047	photosystem II light harvesting complex gene 2.3	109394	photosynthesis
37			X	Vocar.0016s0243	light-harvesting chlorophyll-protein complex I subunit A4	106691	photosynthesis
38		X		Vocar.0009s0275	glycine-rich RNA-binding protein 2	106014	protein synthesis and degradation
39	X	X	X	Vocar.0027s0094	photosystem II reaction center W	104883	photosynthesis
40		X		Vocar.0007s0404	unknown function	104366	other functions
41			X	Vocar.0012s0221	photosystem II light harvesting complex gene 2.2	101764	photosynthesis
42			X	Vocar.0016s0158	photosystem I subunit I	101186	photosynthesis
43			X	Vocar.0001s0400	oxygen evolving enhancer protein 3 (psbQ)	96305	photosynthesis
44			X	Vocar.0002s0040	Golgi to ER traffic protein 4 homolog	95771	other functions
45			X	Vocar.0027s0034	root FNR 2	94359	other functions
46	X	X	X	Vocar.0036s0111	ribosomal protein L1p/L10e family	93636	protein synthesis and degradation
47			X	Vocar.0011s0017	photosystem I light harvesting complex gene 5	89487	photosynthesis
48	X	X	X	Vocar.0016s0012	ribosomal protein 1	87530	protein synthesis and degradation
49		X		Vocar.0001s1775	photosystem II light harvesting complex gene 2.3	87337	photosynthesis
50		X		Vocar.0009s0214	ribosomal protein L4/L1 family	85588	protein synthesis and degradation
