# Supplementary material for: Whole transcriptome RNA-Seq analysis reveals extensive cell type-specific compartmentalization in Volvox carteri
Source: BMC Biol. 2017 Nov 28;15:111. doi: 10.1186/s12915-017-0450-y (PMC5704591; doi:10.1186/s12915-017-0450-y)
Supplement: Supplementary file 6 — Functional enrichment analysis of the most overexpressed genes of both cell types. (PDF 54 kb) [file 12915_2017_450_MOESM6_ESM.pdf]

**Additional file 6: Table S5. Functional enrichment analysis of the most overexpressed genes of both cell types.**

**top 100 fold difference - somatic cells (compared to the other cell type)**

| rank in fold difference | locusName | defline | baseMean somatic cells | baseMean reproductive cells | fold difference | functional group |
|---|---|---|---|---|---|---|
| 1 | Vocar.0002s0260 | unknown function | 36.8 | 0.3 | 142.7 | *[unknown]* |
| 2 | Vocar.0904s0001 | unknown function | 20936.2 | 157.8 | 132.7 | *[unknown]* |
| 3 | Vocar.0904s0002 | unknown function | 476.5 | 4.0 | 118.8 | *[unknown]* |
| 4 | Vocar.0034s0103 | unknown function | 59.5 | 1.2 | 51.7 | *[unknown]* |
| 5 | Vocar.0026s0055 | unknown function | 116.6 | 2.7 | 42.4 | *[unknown]* |
| 6 | Vocar.0075s0010 | unknown function | 111.8 | 2.7 | 42.0 | *[unknown]* |
| 7 | Vocar.0003s0420 | aprataxin-like protein, RNA ligase, DNA ligase III | 19989.4 | 489.7 | 40.8 | other functions |
| 8 | Vocar.0021s0002 | unknown function | 17563.5 | 480.8 | 36.5 | *[unknown]* |
| 9 | Vocar.0321s0002 | unknown function | 40.7 | 1.1 | 35.5 | *[unknown]* |
| 10 | Vocar.0374s0001 | blackjack, microtubule associated protein | 699.8 | 20.7 | 33.8 | flagella associated |
| 11 | Vocar.0041s0047 | tetratricopeptide repeat protein; kinesin light chain | 3211.6 | 96.0 | 33.5 | cytoskeleton |
| 12 | Vocar.0006s0060 | unknown function | 1769.5 | 53.1 | 33.3 | *[unknown]* |
| 13 | Vocar.0004s0368 | pherophorin | 443.9 | 13.4 | 33.1 | ECM compound |
| 14 | Vocar.0006s0207 | glycosyltransferase 14 family protein | 2917.5 | 88.6 | 32.9 | other functions |
| 15 | Vocar.0007s0063 | unknown function | 25.0 | 0.8 | 32.3 | *[unknown]* |
| 16 | Vocar.0059s0041 | small cysteine-rich extracellular protein | 122224.3 | 3964.0 | 30.8 | ECM compound |
| 17 | Vocar.0004s0369 | pherophorin (duf3707) | 19521.2 | 633.9 | 30.8 | ECM compound |
| 18 | Vocar.0050s0060 | Bacteriorhodopsin-like protein | 5707.7 | 186.3 | 30.6 | other functions |
| 19 | Vocar.0014s0094 | unknown function | 59.6 | 2.0 | 30.1 | *[unknown]* |
| 20 | Vocar.0007s0026 | unknown function | 2253.0 | 74.9 | 30.1 | *[unknown]* |
| 21 | Vocar.0062s0031 | gametolysin / lysin | 3501.6 | 118.5 | 29.5 | ECM compound |
| 22 | Vocar.0016s0268 | shippo-1-related, PGP motif | 3079.1 | 105.9 | 29.1 | flagella associated |
| 23 | Vocar.0019s0251 | ammonium transporter | 477.9 | 16.5 | 29.0 | other functions |
| 24 | Vocar.0075s0009 | disintegrin / metalloproteinase | 87.4 | 3.0 | 29.0 | ECM compound |
| 25 | Vocar.0029s0048 | unknown function | 33.0 | 1.1 | 28.8 | *[unknown]* |
| 26 | Vocar.0034s0056 | guanylate cyclase | 4418.8 | 155.0 | 28.5 | other functions |
| 27 | Vocar.0017s0072 | unknown function | 28077.7 | 985.8 | 28.5 | *[unknown]* |
| 28 | Vocar.0062s0034 | pr-1 like protein | 2205.0 | 77.4 | 28.5 | pathogen defense |
| 29 | Vocar.0005s0200 | tetratricopeptide repeat protein; kinesin light chain | 3461.3 | 122.0 | 28.4 | cytoskeleton |
| 30 | Vocar.0013s0137 | unknown function | 423.8 | 15.4 | 27.5 | *[unknown]* |
| 31 | Vocar.0051s0012 | unknown function | 3632.0 | 133.4 | 27.2 | *[unknown]* |
| 32 | Vocar.0003s0333 | unknown function | 1513.8 | 55.6 | 27.2 | *[unknown]* |
| 33 | Vocar.0034s0075 | pherophorin | 124.9 | 4.6 | 27.2 | ECM compound |
| 34 | Vocar.0013s0248 | centrin2, EF-hand domain pair, calcium binding domair | 5607.0 | 206.5 | 27.1 | other functions |
| 35 | Vocar.0007s0123 | unknown function | 352.3 | 13.0 | 27.1 | *[unknown]* |
| 36 | Vocar.0019s0250 | unknown function | 550.6 | 20.4 | 27.0 | *[unknown]* |
| 37 | Vocar.0002s0257 | stop protein, neuronal microtubule stability | 4026.7 | 151.0 | 26.7 | flagella associated |
| 38 | Vocar.0025s0001 | unknown function | 2097.4 | 79.1 | 26.5 | *[unknown]* |

| | | | | | | |
|---|---|---|---|---|---|---|
| 39 | Vocar.0008s0372 | unknown function | 712.7 | 27.0 | 26.4 | [unknown] |
| 40 | Vocar.0025s0040 | dopamine beta hydroxylase related | 2240.1 | 85.6 | 26.2 | other functions |
| 41 | Vocar.0015s0002 | pr-1 like protein | 740.9 | 28.4 | 26.0 | pathogen defense |
| 42 | Vocar.0062s0033 | sterol esterase / gametolysin peptidase m11 | 1007.5 | 39.1 | 25.8 | ECM compound |
| 43 | Vocar.0005s0209 | flagella associated protein | 14501.7 | 562.8 | 25.8 | flagella associated |
| 44 | Vocar.0005s0103 | unknown function | 3002.0 | 116.5 | 25.8 | [unknown] |
| 45 | Vocar.0020s0152 | transducin family protein / WD-40 repeat family protein | 6655.9 | 260.9 | 25.5 | cell division |
| 46 | Vocar.0016s0015 | unknown function | 227.5 | 8.9 | 25.5 | [unknown] |
| 47 | Vocar.0008s0351 | myosin light chain kinase | 51.2 | 2.0 | 25.4 | cytoskeleton |
| 48 | Vocar.0019s0149 | unknown function | 4028.5 | 158.9 | 25.4 | [unknown] |
| 49 | Vocar.0012s0267 | unknown function | 1298.3 | 51.2 | 25.3 | [unknown] |
| 50 | Vocar.0016s0113 | unknown function | 4110.5 | 162.8 | 25.3 | [unknown] |
| 51 | Vocar.0059s0039 | Cysteine proteinases superfamily protein, cathepsin B1 | 259801.3 | 10462.6 | 24.8 | other functions |
| 52 | Vocar.0001s1576 | calpain-type cysteine protease family, Calpain-2 | 1582.3 | 63.7 | 24.8 | other functions |
| 53 | Vocar.0002s0553 | dynein heavy chain 6, axonemal | 105.6 | 4.3 | 24.7 | cytoskeleton |
| 54 | Vocar.0011s0149 | unknown function | 74.9 | 3.0 | 24.6 | [unknown] |
| 55 | Vocar.0020s0135 | scavenger receptor cys-rich | 1258.7 | 51.2 | 24.6 | pathogen defense |
| 56 | Vocar.0006s0353 | unknown function | 28.9 | 1.2 | 24.5 | [unknown] |
| 57 | Vocar.0006s0059 | unknown function | 241.8 | 9.9 | 24.4 | [unknown] |
| 58 | Vocar.0007s0156 | kinesin 1, kinesin FAP125 | 7364.3 | 303.4 | 24.3 | cytoskeleton |
| 59 | Vocar.0020s0212 | metallo-peptidase family M12B reprolysin-like | 7531.8 | 312.2 | 24.1 | ECM compound |
| 60 | Vocar.0005s0015 | serineaspartate repeat-containing protein, ca binding | 837.2 | 35.5 | 23.6 | ECM compound |
| 61 | Vocar.0046s0015 | pherophorin | 67633.4 | 2871.5 | 23.6 | ECM compound |
| 62 | Vocar.0014s0099 | unknown function | 4216.1 | 179.4 | 23.5 | [unknown] |
| 63 | Vocar.0031s0008 | hemingway homologue, sperm flagella assembly | 15274.8 | 652.6 | 23.4 | flagella associated |
| 64 | Vocar.0006s0468 | unknown function | 1380.3 | 59.0 | 23.4 | [unknown] |
| 65 | Vocar.0065s0001 | unknown function | 227.8 | 9.8 | 23.3 | [unknown] |
| 66 | Vocar.0016s0016 | unknown function | 5259.8 | 227.6 | 23.1 | [unknown] |
| 67 | Vocar.0012s0283 | unknown function | 17836.1 | 773.9 | 23.0 | [unknown] |
| 68 | Vocar.0004s0251 | ras group-related LRR 1, Ras suppressor protein | 1109.4 | 48.2 | 23.0 | other functions |
| 69 | Vocar.0004s0511 | 2-oxoglutarate (2OG) and Fe(II)-dependent oxygenase | 5923.5 | 257.2 | 23.0 | other functions |
| 70 | Vocar.0004s0423 | MYND finger (zf-MYND) containing protein | 11793.4 | 518.1 | 22.8 | other functions |
| 71 | Vocar.0012s0268 | unknown function | 918.1 | 40.4 | 22.7 | [unknown] |
| 72 | Vocar.0013s0115 | protein cal-5 (calcium binding) | 4062.0 | 179.3 | 22.6 | other functions |
| 73 | Vocar.0004s0255 | extracellular matrix glycoprotein pherophorin I | 158963.2 | 7038.4 | 22.6 | ECM compound |
| 74 | Vocar.0057s0045 | unknown function | 8555.9 | 379.8 | 22.5 | [unknown] |
| 75 | Vocar.0034s0013 | cell division control 2, cyclin-dependent kinase-like 5 | 20711.1 | 924.6 | 22.4 | cell division |
| 76 | Vocar.0005s0167 | Protein kinase superfamily protein,calmodulin-binding | 9486.7 | 425.5 | 22.3 | other functions |
| 77 | Vocar.0016s0307 | ADP-ribosylation factor B1A | 808.4 | 36.3 | 22.3 | other functions |
| 78 | Vocar.0019s0248 | periodic tryptophan protein 2 | 6178.1 | 277.8 | 22.2 | other functions |
| 79 | Vocar.0039s0015 | unknown function | 3099.3 | 139.4 | 22.2 | [unknown] |
| 80 | Vocar.0028s0145 | MOS4-associated complex 3B; WD40 repeat protein | 5289.5 | 239.3 | 22.1 | other functions |
| 81 | Vocar.0026s0030 | unknown function | 2254.5 | 102.1 | 22.1 | [unknown] |
| 82 | Vocar.0002s0489 | sperm-tail PG-rich repeat (shippo-rpt) | 30251.2 | 1381.2 | 21.9 | flagella associated |
| 83 | Vocar.0015s0078 | unknown function | 3294.5 | 150.4 | 21.9 | [unknown] |
| 84 | Vocar.0001s1194 | flagella associated protein | 17255.7 | 788.9 | 21.9 | flagella associated |
| 85 | Vocar.0024s0216 | pherophorin (DUF3707) | 932.9 | 42.7 | 21.9 | ECM compound |
| 86 | Vocar.0028s0147 | flagella associated protein | 3766.3 | 172.6 | 21.8 | flagella associated |

| | | | | | | |
|---|---|---|---|---|---|---|
| 87 | Vocar.0015s0126 | flagella associated protein | 6113.7 | 280.9 | 21.8 | flagella associated |
| 88 | Vocar.0014s0054 | unknown function | 5779.0 | 265.8 | 21.7 | *[unknown]* |
| 89 | Vocar.0015s0258 | adenosine monophosphate kinase, KPL2-related | 29278.0 | 1351.0 | 21.7 | flagella associated |
| 90 | Vocar.0001s0591 | flagella associated protein | 4936.9 | 229.4 | 21.5 | flagella associated |
| 91 | Vocar.0012s0001 | unknown function | 2360.0 | 109.8 | 21.5 | *[unknown]* |
| 92 | Vocar.0015s0006 | unknown function | 196.9 | 9.2 | 21.5 | *[unknown]* |
| 93 | Vocar.0006s0264 | flagella associated protein | 2024.0 | 94.4 | 21.4 | flagella associated |
| 94 | Vocar.0005s0122 | nuclear control of ATPase protein 2, flagella associatec | 20987.6 | 981.4 | 21.4 | flagella associated |
| 95 | Vocar.0028s0005 | flagella associated protein | 14716.2 | 690.5 | 21.3 | flagella associated |
| 96 | Vocar.0016s0002 | spermatogenesis-associated protein 4 | 3638.5 | 171.1 | 21.3 | flagella associated |
| 97 | Vocar.0070s0007 | NGS-gene like, nitrogen starvation gene | 321905.6 | 15149.4 | 21.2 | other functions |
| 98 | Vocar.0007s0124 | NAD(P)-linked oxidoreductase superfamily protein | 8065.2 | 380.3 | 21.2 | other functions |
| 99 | Vocar.0030s0118 | unknown function | 5789.4 | 273.0 | 21.2 | *[unknown]* |
| 100 | Vocar.0004s0402 | transient receptor potential channel | 5703.6 | 269.2 | 21.2 | other functions |

**top 100 fold difference - reproductive cells (compared to the other cell type)**

| rank in fold difference | locusName | defline | baseMean somatic cells | baseMean reproductive cells | fold difference | functional group |
|---|---|---|---|---|---|---|
| 1 | Vocar.0002s0575 | myb domain protein 3r-5, myb-like DNA-binding proteir | 0.5 | 454.9 | 900.6 | cell division |
| 2 | Vocar.0004s0462 | unknown function | 1.5 | 384.5 | 248.6 | *[unknown]* |
| 3 | Vocar.0021s0094 | peptidoglycan binding domain, topoisomerase | 0.5 | 124.5 | 246.6 | pathogen defense |
| 4 | Vocar.0013s0228 | unknown function | 0.5 | 115.5 | 228.7 | *[unknown]* |
| 5 | Vocar.0033s0161 | plus-end-directed kinesin ATPase / kinesin | 6.2 | 988.1 | 158.9 | cytoskeleton |
| 6 | Vocar.0002s0013 | unknown function | 4.3 | 627.8 | 145.7 | *[unknown]* |
| 7 | Vocar.0002s0038 | unknown function | 3.6 | 518.6 | 142.4 | *[unknown]* |
| 8 | Vocar.0002s0605 | unknown function | 3.4 | 465.5 | 135.0 | *[unknown]* |
| 9 | Vocar.0007s0165 | cytochrome B561 / ferric reductase, transmembrane | 2.1 | 267.5 | 127.6 | other functions |
| 10 | Vocar.0039s0020 | cell adhesion molecule, algal-CAM | 19.9 | 2509.1 | 126.0 | ECM compound |
| 11 | Vocar.0002s0216 | unknown function | 1.4 | 172.1 | 123.2 | *[unknown]* |
| 12 | Vocar.0020s0148 | unknown function | 0.3 | 34.5 | 118.8 | *[unknown]* |
| 13 | Vocar.0053s0065 | diphthamide synthesis DPH2 family protein | 1.7 | 196.4 | 116.4 | other functions |
| 14 | Vocar.0025s0185 | transcription activator, Nckap1 related | 2.0 | 232.3 | 114.5 | gene regulation |
| 15 | Vocar.0055s0039 | JmjC domain-containing histone demethylation protein | 3.6 | 401.1 | 110.1 | gene regulation |
| 16 | Vocar.0019s0016 | dynamin-like protein ARC5 | 2.4 | 258.8 | 109.5 | cell division |
| 17 | Vocar.0011s0353 | chloroplast division site determinant protein | 0.7 | 72.3 | 106.9 | cell division |
| 18 | Vocar.0042s0032 | unknown function | 0.4 | 41.1 | 106.4 | *[unknown]* |
| 19 | Vocar.0001s1647 | JmjC domain-containing histone demethylation protein | 5.4 | 544.4 | 101.3 | gene regulation |
| 20 | Vocar.0032s0001 | unknown function | 0.9 | 86.6 | 97.2 | *[unknown]* |
| 21 | Vocar.0004s0179 | chromatin binding proten SWI/SNF-related | 13.8 | 1288.3 | 93.3 | gene regulation |
| 22 | Vocar.0019s0201 | unknown function | 2.2 | 202.5 | 93.3 | *[unknown]* |
| 23 | Vocar.0006s0181 | N1-methyltransferase | 1.6 | 146.6 | 92.2 | other functions |
| 24 | Vocar.0029s0079 | gag polypeptide of LTR retrotransposon, copia type | 16.4 | 1501.7 | 91.4 | other functions |
| 25 | Vocar.0018s0129 | BACK protein with BTB, C-terminal Kelch domain | 2.0 | 173.5 | 85.8 | other functions |
| 26 | Vocar.0003s0030 | DNA primases; small subunit | 4.8 | 409.5 | 85.6 | cell division |
| 27 | Vocar.0021s0184 | helicase, minichromosome maintenance protein 3 | 12.6 | 1027.7 | 81.7 | cell division |

| | | | | | | |
|---|---|---|---|---|---|---|
| 28 | Vocar.0002s0282 | unknown function | 1.9 | 153.0 | 80.5 | *[unknown]* |
| 29 | Vocar.0015s0250 | UvrB/UvrC domain-containing protein | 3.6 | 283.8 | 79.6 | other functions |
| 30 | Vocar.0018s0186 | unknown function | 2.6 | 204.3 | 79.3 | *[unknown]* |
| 31 | Vocar.0001s0263 | unknown function | 1.7 | 134.3 | 77.1 | *[unknown]* |
| 32 | Vocar.0012s0232 | unknown function | 9.2 | 704.8 | 76.8 | *[unknown]* |
| 33 | Vocar.0021s0129 | unknown function | 15.4 | 1168.1 | 76.1 | *[unknown]* |
| 34 | Vocar.0003s0025 | pherophorin | 182.0 | 13842.4 | 76.0 | ECM compound |
| 35 | Vocar.0036s0102 | unknown function | 26.2 | 1973.2 | 75.4 | *[unknown]* |
| 36 | Vocar.0006s0454 | septum site-determining protein, MIND homolog | 13.8 | 1037.4 | 75.3 | cell division |
| 37 | Vocar.0001s0298 | pherophorin | 139.5 | 10295.9 | 73.8 | ECM compound |
| 38 | Vocar.0020s0211 | unknown function | 0.8 | 55.1 | 71.4 | *[unknown]* |
| 39 | Vocar.0011s0177 | G2/mitotic-specific cyclin A | 4.0 | 279.2 | 70.7 | cell division |
| 40 | Vocar.0011s0182 | unknown function | 11.1 | 788.0 | 70.7 | *[unknown]* |
| 41 | Vocar.0014s0064 | myosin light-chain kinase | 3.6 | 243.8 | 68.3 | cytoskeleton |
| 42 | Vocar.0024s0192 | RING/U-box protein witch Zinc finger, C3HC4 type | 7.0 | 469.3 | 66.9 | gene regulation |
| 43 | Vocar.0002s0462 | sterol 4-alpha-methyl-oxidase 2-2 | 1.2 | 77.1 | 66.6 | other functions |
| 44 | Vocar.0073s0026 | RING/FYVE/PHD zinc finger superfamily protein | 10.8 | 706.8 | 65.6 | gene regulation |
| 45 | Vocar.0003s0022 | unknown function | 6.0 | 394.5 | 65.6 | *[unknown]* |
| 46 | Vocar.0184s0001 | unknown function | 0.9 | 58.2 | 65.3 | *[unknown]* |
| 47 | Vocar.0439s0001 | unknown function | 1.7 | 107.5 | 64.6 | *[unknown]* |
| 48 | Vocar.0031s0098 | ribonucleoside-diphosphate reductase small chain | 27.5 | 1762.2 | 64.0 | cell division |
| 49 | Vocar.0027s0113 | unknown function | 51.3 | 3253.0 | 63.4 | *[unknown]* |
| 50 | Vocar.0001s0297 | pherophorin (DUF3707) | 5.6 | 340.3 | 60.6 | ECM compound |
| 51 | Vocar.0030s0092 | flagellar associated protein | 4.1 | 250.2 | 60.4 | flagella associated |
| 52 | Vocar.0003s0026 | pherophorin | 454.2 | 27323.7 | 60.2 | ECM compound |
| 53 | Vocar.0036s0088 | kinetochore protein NDC80, spindle checkp. Signalling | 11.7 | 667.0 | 57.2 | cell division |
| 54 | Vocar.0003s0021 | pherophorin (DUF3707) | 1.4 | 78.8 | 56.4 | ECM compound |
| 55 | Vocar.0008s0168 | unknown function | 0.9 | 49.2 | 55.2 | *[unknown]* |
| 56 | Vocar.0003s0023 | pherophorin | 255.3 | 13981.7 | 54.8 | ECM compound |
| 57 | Vocar.0029s0112 | unknown function | 30.5 | 1671.8 | 54.8 | *[unknown]* |
| 58 | Vocar.0036s0103 | unknown function | 20.7 | 1130.6 | 54.6 | *[unknown]* |
| 59 | Vocar.0075s0002 | unknown function | 151.1 | 8138.0 | 53.9 | *[unknown]* |
| 60 | Vocar.0003s0321 | unknown function | 1.0 | 54.3 | 53.7 | *[unknown]* |
| 61 | Vocar.0013s0164 | unknown function | 1.2 | 61.8 | 53.3 | *[unknown]* |
| 62 | Vocar.0006s0320 | proliferating cell nuclear antigen 2 | 56.8 | 3013.2 | 53.1 | cell division |
| 63 | Vocar.0001s1329 | serine/threonine-protein kinase TNNI3K | 0.5 | 26.5 | 52.5 | other functions |
| 64 | Vocar.0013s0200 | PAN-like domain (PAN_3), carbohydrate-binding | 6.2 | 324.7 | 52.2 | other functions |
| 65 | Vocar.0017s0205 | unknown function | 4.9 | 256.6 | 52.1 | *[unknown]* |
| 66 | Vocar.0005s0369 | haloacid dehalogenase-like hydrolase (HAD) protein | 2.6 | 135.9 | 51.7 | other functions |
| 67 | Vocar.0027s0096 | ankyrin repeat protein (cytosceleton associated) | 49.4 | 2545.7 | 51.6 | cytoskeleton |
| 68 | Vocar.0001s0226 | unknown function | 26.6 | 1364.6 | 51.3 | *[unknown]* |
| 69 | Vocar.0029s0111 | Cyclin A1, G2/mitotic-specific cyclin B | 15.4 | 778.9 | 50.4 | cell division |
| 70 | Vocar.0003s0405 | kinesin-like protein 1 | 26.8 | 1346.1 | 50.3 | cytoskeleton |
| 71 | Vocar.0031s0085 | ribonucleotide reductase 1, ribonucleoside-diphosphate | 52.6 | 2643.8 | 50.3 | cell division |
| 72 | Vocar.0061s0028 | Metal-dependent phosphohydrolase, cyclin-L1-1 | 14.3 | 717.1 | 50.3 | cell division |
| 73 | Vocar.0039s0070 | RecQ-mediated genome instability protein 2 (RMI2) | 6.7 | 335.6 | 50.1 | cell division |
| 74 | Vocar.0001s0749 | DNA-binding enhancer protein-related | 7.7 | 383.5 | 49.8 | gene regulation |
| 75 | Vocar.0004s0285 | unknown function | 58.5 | 2905.9 | 49.7 | *[unknown]* |

| | | | | | | |
|---|---|---|---|---|---|---|
| 76 | Vocar.0001s0882 | tRNA/rRNA methyltransferase (SpoU) family protein | 1.9 | 93.2 | 49.0 | gene regulation |
| 77 | Vocar.0005s0001 | unknown function | 46.9 | 2279.6 | 48.6 | *[unknown]* |
| 78 | Vocar.0024s0154 | unknown function | 12.3 | 598.1 | 48.6 | *[unknown]* |
| 79 | Vocar.0019s0200 | phragmoplast orienting kinesin 2, putative kinesin K39 | 3.2 | 152.1 | 47.8 | cell division |
| 80 | Vocar.0045s0055 | sumo conjugation enzyme 1 | 9.2 | 428.7 | 46.5 | other functions |
| 81 | Vocar.0034s0027 | unknown function | 1.5 | 70.2 | 46.3 | *[unknown]* |
| 82 | Vocar.0001s0192 | Transcription factor DP, E2F interaction | 42.4 | 1960.9 | 46.2 | cell division |
| 83 | Vocar.0026s0184 | chloroplast outer envelope protein | 8.4 | 385.7 | 45.7 | other functions |
| 84 | Vocar.0009s0116 | unknown function | 2.0 | 93.3 | 45.5 | *[unknown]* |
| 85 | Vocar.0033s0046 | plastid division protein FtsZ1 | 51.3 | 2331.7 | 45.4 | cell division |
| 86 | Vocar.0003s0466 | unknown function | 2.3 | 104.7 | 45.3 | *[unknown]* |
| 87 | Vocar.0025s0165 | DNA-directed DNA polymerase alpha | 22.0 | 991.5 | 45.0 | cell division |
| 88 | Vocar.0001s0295 | pherophorin (DUF3707) | 34.7 | 1559.2 | 45.0 | ECM compound |
| 89 | Vocar.0004s0233 | unknown function | 3.8 | 170.6 | 44.7 | *[unknown]* |
| 90 | Vocar.0025s0064 | unknown function | 6.9 | 306.9 | 44.6 | *[unknown]* |
| 91 | Vocar.0018s0226 | unknown function | 0.6 | 25.8 | 44.4 | *[unknown]* |
| 92 | Vocar.0032s0002 | unknown function | 1.9 | 81.6 | 43.9 | *[unknown]* |
| 93 | Vocar.0001s0296 | pherophorin (DUF3707) | 17.1 | 750.4 | 43.9 | ECM compound |
| 94 | Vocar.0001s0450 | class I glutamine amidotransferase-like protein | 3.3 | 142.0 | 43.1 | other functions |
| 95 | Vocar.0009s0333 | unknown function | 1.9 | 81.8 | 43.0 | *[unknown]* |
| 96 | Vocar.0001s0294 | pherophorin | 13.8 | 593.8 | 42.9 | ECM compound |
| 97 | Vocar.0001s1158 | DegP protease 10, HtrA2 peptidase / serine proteinase | 1.5 | 64.5 | 42.6 | other functions |
| 98 | Vocar.0011s0135 | pyruvate dehydrogenase kinase | 16.9 | 716.5 | 42.3 | other functions |
| 99 | Vocar.0003s0489 | unknown function | 19.4 | 822.1 | 42.3 | *[unknown]* |
| 100 | Vocar.0005s0002 | unknown function | 37.6 | 1584.4 | 42.2 | *[unknown]* |
