# Supplementary material for: Whole transcriptome RNA-Seq analysis reveals extensive cell type-specific compartmentalization in Volvox carteri
Source: BMC Biol. 2017 Nov 28;15:111. doi: 10.1186/s12915-017-0450-y (PMC5704591; doi:10.1186/s12915-017-0450-y)
Supplement: Supplementary file 7 — Examples of typical expression profiles of genes with low, average, and high expression. (PDF 236 kb) [file 12915_2017_450_MOESM7_ESM.pdf]

Additional file 7: Figure S2. Examples of typical expression profiles of genes with low, average, and high expression.

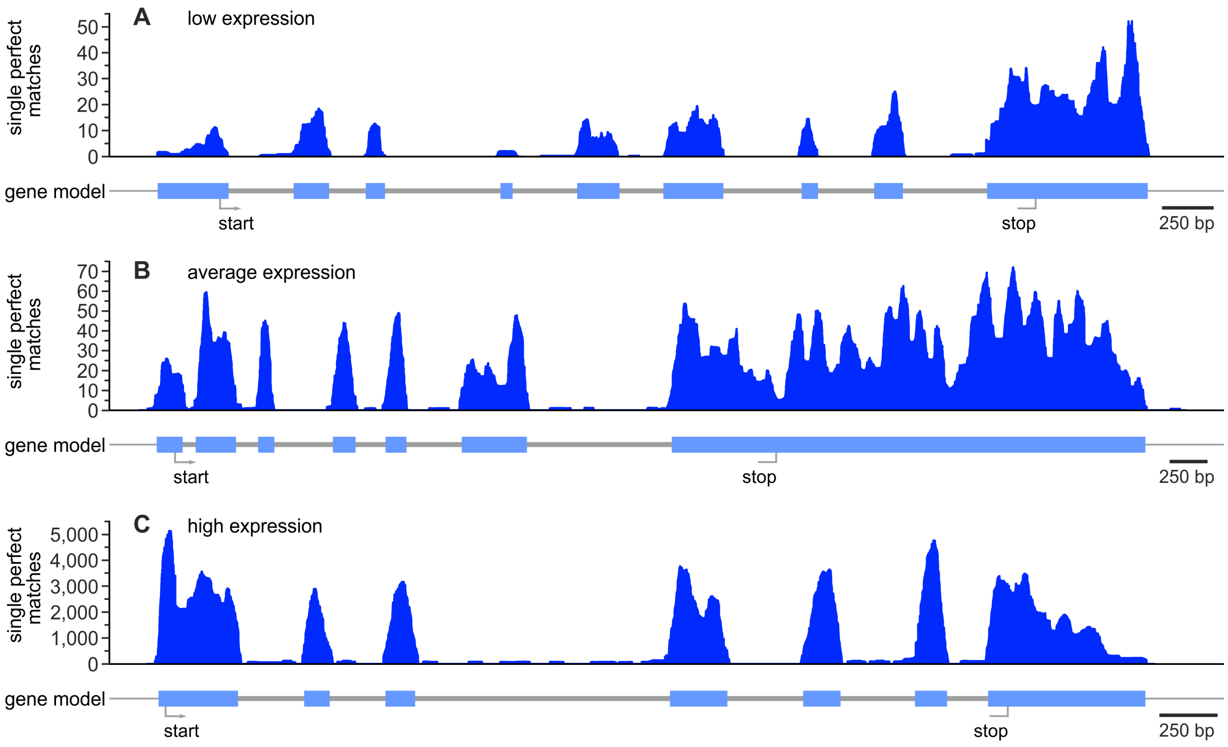

(**A**) Example of a typical expression profile of a gene with low expression: gene Vocar.0009s0331 with a baseMean expression value of 451 in reproductive cells. In our data analysis, a baseMean expression value of 450 was still sufficient to allow for quality control of gene predictions. (**B**) Example of a typical expression profile of a gene with average expression: gene Vocar.0070s0008 with a baseMean expression value of 2740 in somatic cells. The average of all baseMean expression values in our dataset is 2822. (**C**) Example of a typical expression profile of a gene with high expression: gene Vocar.0009s0214 with a baseMean expression value of 83650 in reproductive cells. The expression of this gene is ranked 50th among all genes or, to put it another way, it is ranked among the top 0.5% of all genes in terms of expression intensity. (**A-C**) The exon-intron structure and the position of start and stop codons is illustrated below each expression profile. Exons are shown as blue bars and introns as thick gray lines. The corresponding data come from gene annotation version 2.1 of the *Volvox carteri* genome available on the *Volvox* pages of the Phytozome V12 platform. The length of these example genes and the number of their introns is quite average in the *V. carteri* genome (Prochnik et al. 2010).

Reference

Prochnik SE, Umen J, Nedelcu AM, Hallmann A, Miller SM, Nishii I, Ferris P, Kuo A, Mitros T, Fritz-Laylin LK, Hellsten U, Chapman J, Simakov O, Rensing SA, Terry A, Pangilinan J, Kapitonov V, Jurka J, Salamov A, Shapiro H, Schmutz J, Grimwood J, Lindquist E, Lucas S, Grigoriev IV, Schmitt R, Kirk D, Rokhsar DS (2010) Genomic analysis of organismal complexity in the multicellular green alga *Volvox carteri*. Science 329: 223-226
